# Computable early *Caenorhabditis elegans* embryo with a phase field model

**DOI:** 10.1371/journal.pcbi.1009755

**Published:** 2022-01-14

**Authors:** Xiangyu Kuang, Guoye Guan, Ming-Kin Wong, Lu-Yan Chan, Zhongying Zhao, Chao Tang, Lei Zhang

**Affiliations:** 1 Center for Quantitative Biology, Peking University, Beijing, China; 2 Department of Biology, Hong Kong Baptist University, Hong Kong, China; 3 State Key Laboratory of Environmental and Biological Analysis, Hong Kong Baptist University, Hong Kong, China; 4 Peking-Tsinghua Center for Life Sciences, Peking University, Beijing, China; 5 School of Physics, Peking University, Beijing, China; 6 Beijing International Center for Mathematical Research, Peking University, Beijing, China; Duke University, UNITED STATES

## Abstract

Morphogenesis is a precise and robust dynamic process during metazoan embryogenesis, consisting of both cell proliferation and cell migration. Despite the fact that much is known about specific regulations at molecular level, how cell proliferation and migration together drive the morphogenesis at cellular and organismic levels is not well understood. Using *Caenorhabditis elegans* as the model animal, we present a phase field model to compute early embryonic morphogenesis within a confined eggshell. With physical information about cell division obtained from three-dimensional time-lapse cellular imaging experiments, the model can precisely reproduce the early morphogenesis process as seen *in vivo*, including time evolution of location and morphology of each cell. Furthermore, the model can be used to reveal key cell-cell attractions critical to the development of *C*. *elegans* embryo. Our work demonstrates how genetic programming and physical forces collaborate to drive morphogenesis and provides a predictive model to decipher the underlying mechanism.

## Author summary

Embryonic development is a precise process involving cell division, cell-cell interaction, and cell migration. During the process, how each cell reaches its supposed location and be in contact with the right neighbors, and what roles genetic factors and physical forces play are important and fascinating questions. Using the worm *Caenorhabditis elegans* as a model system, we build a phase field model to simulate early morphogenesis. With a few physical inputs, the model can precisely reproduce the early morphological development of the worm. Such an accurate simulator can not only teach us how physical forces work together with genetic factors to shape up the complex process of development, but also make predictions, such as key cell-cell attractions critical in the process.

## Introduction

The development of metazoan embryos consists of cell proliferation, cell migration, and cell differentiation, which is robust against perturbations and reproducible among individuals [1–3]. The system evolves from a fertilized zygote to a multicellular structure with hundreds to thousands of cells and forms stereotypic three-dimensional spatial patterns [4–6]. These morphogenetic dynamics are achieved by the precise control on cell divisions [7,8], mechanical interactions between cells [9,10], and other molecular-level regulations like cortical myosin flow, inhomogeneous cytomembrane adhesion, and active actomyosin contractility [11–13]. A fundamental question in developmental biology is “Will the egg be computable?” [14].

The eutelic nematode *Caenorhabditis elegans* has an invariant developmental procedure at the cellular level, i.e., each cell can be identified based on its lineage history and has highly reproducible division timing, division orientation, migration trajectory, and cell fate among individual embryos [2,4]. Morphogenesis in *C*. *elegans* starts as early as fertilization and the cell specification happens intensively afterward. For example, there are 4 cell types at 4-cell stage and 6 cell types at 8-cell stage—the cell types are diversified by the consecutive asymmetric divisions of the germline stem cell (i.e., P0, P1, P2, and P3) and the contact-based cell-cell signaling transductions (e.g., Wnt and Notch) [15–17]. These cells of different sizes, shapes, and fates migrate, communicate and interact with each other, making the correctness of their positions and contacts extremely momentous.

Extensive theoretical studies have been carried out using *C*. *elegans* embryo as a model organism for developmental biology and great efforts have been made to computationally rebuild the morphogenetic behaviors *in silico*, with an ultimate goal of permitting virtual experiments to facilitate in-depth interpretation and understanding of embryonic morphogenesis. To list a few, a multi-particle model was designed to analyze the structural evolution up to the 4-cell stage using groups of interactive particles to represent cell membranes, which however contained dozens of system parameters that were difficult to measure or fit [18,19]. Later, coarse-grained models were proposed to reproduce cell positions up to ~50-cell stage, which simplified the cells into mass particles and ignored most of the cellular morphological features [9,10,20–22]. These models revealed several physical factors that could affect the cell-arrangement patterns, such as cell division timing, cell division orientation, cell adhesion, and eggshell shape. Recently, deep-learning methods were applied to extract information from 4-dimensional cell motion data, which by itself did not address the physical and biophysical mechanistic questions [23,24]. On the other hand, phase-field methods have been widely applied to simulate single-cell dynamics including cell morphology and motility [25–29], and to mimic the evolution of multicellular systems on the phenomenological level, such as the early development in sea cucumber and nematode embryos [30–33]. Till now, few studies carefully compared the morphological dynamics between experiment and simulation, partially due to the lack of high-quality quantitative *in vivo* data. For example, the cell shape and cell-cell contact have never been fully reproduced *in silico* even for an 8-cell *C*. *elegans* embryo. Thus, it remains largely elusive whether a model’s mathematical expression fits reality or not, and how precise it is to describe a specific biophysical process or property, such as cell-cell interactions, cell deformation, and cell motion.

In this paper, we present a phase field model combined with *in vivo* cell morphology data, to reconstruct the morphogenetic dynamics in early *C*. *elegans* embryogenesis and investigate the strategies and principles accounting for the stereotypic patterns. We first collect cell-resolved morphological data from our previous work [34], including cell location, cell shape, cell volume, cell surface area, and cell-cell contact relationship and area, and eggshell shape, which were originally acquired from three-dimensional (3D) time-lapse imaging on 4 wild-type embryos. Next, we develop a phase field model to reproduce the morphogenetic transformation from 1- to 4-cell stages by considering minimal mechanical constraints and fitting the parameters according to experimental data. We predict the asymmetry of adhesion among the 5 contacted cell pairs in the diamond-shaped 4-cell structure, in consistency with the previous knowledge [10]. We further simulate the cell division, deformation, and motion from 6- to 8-cell stages by introducing self-determined mechanisms on cell division timing and cell-cell attraction matrix to guide the spatial development automatically. Our model predicts a defective phenotype called “structural planarization” in the compressed embryo when developmental programs are disturbed, which is verified by a laser-ablation experiment. Lastly, using the phase field model in which cell morphology can be accurately simulated, we investigate the effect of three physical factors, i.e., cell division timing, cell division orientation, and cell-cell attraction matrix, on the cell-arrangement progression by systematic simulation and analysis, and unravel their particular functions on the precise and robust morphological evolution at 6-, 7- and 8-cell stages. To sum up, we establish a phase field model that can accurately capture cell morphology, infer biophysical properties, and predict morphological phenotype under different conditions for the *C*. *elegans* embryogenesis *in vivo*.

## Results

### *In vivo* data collection

**Fig 1:**
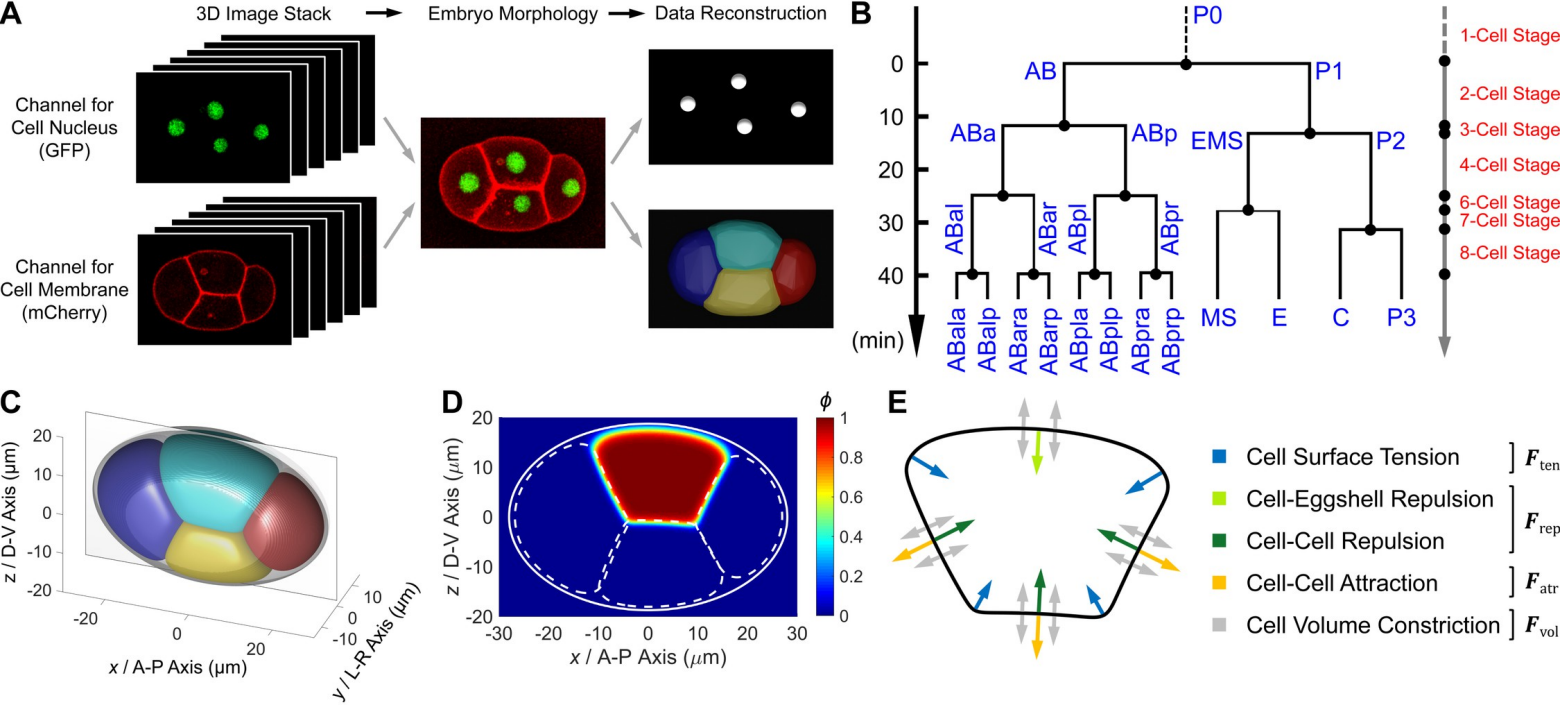
Reconstruction of cellular developmental properties and establishment of phase field model. (A). *In vivo* 3D time-lapse imaging experiment and quantification of cell location (nucleus; GFP, green) and cell morphology (membrane; mCherry, red), illustrated with strain ZZY0535 [40]. The images are obtained from a previous dataset for schematics [2]. (B). Cell lineage tree from 1- to 8-cell stages, including 6 conservatively-ordered cell division groups (i.e., P0 → AB → P1 → ABa and ABp → EMS → P2) and consequently 7 stages with the cell number increasing from 1 to 8 (noted on right). The 8-cell stage ends with the synchronous divisions of ABal, ABar, ABpl, and ABpr. The tree is plotted on an average of 222 wild-type embryos [2]. (C). The eggshell and cell in the phase field model. An ideal eggshell under compression is rebuilt as a boundary based on size measurements on 4 wild-type embryos [34]. The cells which interact via designated forces are constrained within the reconstructed eggshell and illustrated with the 4-cell stage. The *x*-*z* plane is highlighted with a rectangular frame and used to visualize the distribution of phase field in (D). The distribution of phase field across the *x*-*z* plane, illustrated with a heat map using the ABp cell as an example. The boundary of the eggshell is labeled by a solid white line and the boundaries of the other 3 cells (i.e., ABa, EMS, and P2) are labeled by dashed white lines. (E). A sketch map of the forces imposed on a cell in the phase field model. The relationship between force type and color is listed on right.

A total of 18 wild-type embryos imaged by 3D time-lapse confocal microscopy are collected from the datasets produced previously, along with their outputs of membrane segmentation, nucleus tracking, and cell lineaging (S1 Table) [2,34], providing multi-dimensional cell-level developmental properties from 1- to 8-cell stages (Fig 1A; see Materials and methods). The *C*. *elegans* embryonic cell lineage tree containing cell identity and cell division timing is shown in Fig 1B. Briefly, the germline stem cell is named with a prefix “P” and the following number indicating its generation; each somatic founder cell has a specific prefix such as AB, while its descendants acquire a suffix determined by its initial location relative to its sister (i.e., “a” for anterior, “p” for posterior, “l” for left and “r” for right). The cell division orientation, cell volume, and eggshell shape are quantified and directly inputted into the simulation as predetermined parameters (Fig 1C and S2 Table), while the cell location and cell morphology (i.e., cell shape, cell surface area, and cell-cell contact relationship and area) are used to verify our model by comparison to the simulation results (S3–S6 Tables). Note that all the embryos collected in this work are compressed to some extent for a narrower view field and clearer fluorescence signal in imaging, according to a widely used experimental protocol [35–39]. The *C*. *elegans* early development is divided into separate stages with exact cell numbers for step-by-step simulation, according to its invariant cell division sequence *in vivo* (Fig 1B) [2].

### *In silico* model construction

To simulate the morphological and morphogenetic dynamics of a multicellular system, we develop a phase field model which uses a 3D phase field *ϕ*_*i*_ (***r***, *t*) to describe each cell *i* (*i* = 1,…,*N*) (Fig 1D), where *N* is the cell number. The phase field of a cell is subjected to multiple forces including cell surface tension ***F***_ten_, cell-eggshell and cell-cell repulsion ***F***_rep_, cell-cell attraction ***F***_atr_ and cell volume constriction ***F***_vol_ (Fig 1E), whose expressing formulas are selected from previous studies [31,41,42], i.e.,

$$F_{ten}=-\gamma (\Delta \phi_{i}-cW^{\prime }(\phi_{i}))\frac{\nabla \phi_{i}}{{\left|\nabla \phi_{i}\right|}^{2}},$$

$$F_{rep}=\left(g_{e}\phi_{i}\phi_{e}^{2}+g\phi_{i}\sum_{j\neq i}^{N}\phi_{j}^{2}\right)\frac{\nabla \phi_{i}}{{\left|\nabla \phi_{i}\right|}^{2}},$$

$$F_{atr}=\sum_{j\neq i}^{N}\sigma_{i,j}\nabla \phi_{j},$$

$$F_{vol}=M\left(V_{i}(t)-\int_{\Omega }\phi_{i}dr\right)\hat{n},$$

where *γ* is the cell surface tension and *c* is a positive coefficient related to the thickness of boundary between interior and exterior of a cell, namely, cell cortex; *W*(*ϕ*) = *ϕ*^2^(*ϕ*−1)^2^ is a double-well potential with minima at *ϕ* = 0 and *ϕ* = 1; *g*_e_ and *g* are positive coefficients, denoting the strength of cell-eggshell and cell-cell repulsive energy respectively; *σ*_*i*,*j*_ is a non-negative coefficient and positively associated with the attraction intensity between the *i*-th and *j*-th cells; *M* is a positive coefficient which denotes the volume constraint strength and $\hat{n}$ is the unit normal vector at the interface which orients inward; *V*_*i*_(*t*) denotes the prescribed volume for the *i*-th cell and *Ω* is the whole computational domain.

Cell division is implemented as instantaneous bisection of phase field *ϕ*_*i*_ by a splitting plane, whose direction and location are determined by cell volume segregation direction and ratio obtained from experimental measurements (see Materials and Methods). The whole system evolves over developmental time *t* inside an overdamped environment as follows:

$$\frac{\partial \phi_{i}}{\partial t}=-\frac{1}{\tau }(F_{ten}+F_{rep}+F_{atr}+F_{vol})∙\nabla \phi_{i}.$$

where *τ* is the viscosity coefficient of the embryo’s internal environment. To build up a minimal model that has the least physical constraints but outlines the most significant characteristics of a developing embryo, we will modify the system progressively according to the *in vivo* cell morphology data. We initially set the intercellular attraction *σ*_*i*,*j*_ = 0 for all the contacted cell pairs, which would be investigated in depth later as a high-dimensional factor to diversify the path of morphogenesis (hereafter referred to as “developmental path”). Additionally, the independent physical coefficients *γ*, *c*, and *g*_e_ are optimized and fixed by fitting the structural features observed in the experiment from 1- to 4-cell stages (see Materials and Methods). For all simulations in this work, the computational domain is set to be a 256×256×128 cuboid grid with a grid size d*l* = 0.2508 μm and the time step is always set as *h* = 0.1, and all parameters are set once for all except *σ*_*i*,*j*_, to avoid parameter overfitting. The symbols and parameters of phase-field functions above are listed in Table 1 along with their biological and computational meaning. Coupled with the state-of-the-art dataset of *C*. *elegans* embryonic morphology, next we build the phase field model step by step to validate the selected mathematical formulation and achieve accurate and predictive simulation for the real multicellular system.

**Table 1:** The symbols and parameters of phase-field functions.

Parameter	Biological / Computational Meaning	Value Assignment/Function Expression
*ϕ*	cell body / morphology	0 ~ 1
*N*	cell number	1 ~ 8
*i*, *j*	cell identity	1 ~ *N*
*t*	developmental time	/
*V*	cell volume	/
***F***_ten_	force field of cell surface tension	$-\gamma (\Delta \phi_{i}-cW^{\prime }(\phi_{i}))\frac{\nabla \phi_{i}}{{\left|\nabla \phi_{i}\right|}^{2}}$
***F***_rep_	force field of cell-eggshell and cell-cell repulsion	$\left(g_{e}\phi_{i}\phi_{e}^{2}+g\phi_{i}\sum_{j\neq i}^{N}\phi_{j}^{2}\right)\frac{\nabla \phi_{i}}{{\left|\nabla \phi_{i}\right|}^{2}}$
***F***_atr_	force field of cell-cell attraction	$\sum_{j\neq i}^{N}\sigma_{i,j}\nabla \phi_{j}$
***F***_vol_	force field of cell volume constriction	$M\left(V_{i}(t)-\int_{\Omega }\phi_{i}dr\right)\hat{n}$
*c*	thickness of cell cortex / cell boundary	1
*γ*	strength of cell surface tension	0.25
*g*_e_	strength of eggshell stiffness	16
*g*	strength of cell stiffness	1.6
*σ*	strength of cell-cell attraction	/
*σ*’_S_	strength of especially strong cell-cell attraction	1.6
*σ*_S_	strength of relatively strong cell-cell attraction	0.9
*σ*_W_	strength of relatively weak cell-cell attraction	0.2
*τ*	viscosity coefficient of embryo’s internal environment	2.62
*M*	strength of cell volume constriction	0.0012
*W*	double-well function that separates the cell into two phases	*ϕ*^2^(*ϕ*−1)^2^
$\hat{n}$	unit normal vector at the interface which orients inward	/
*Ω*	cuboid domain for computation	256×256×128

### The cell morphology from 1- to 4-cell stages can be reconstructed by phase field model using only cell surface tension, cell-eggshell and cell-cell repulsion, and cell volume constriction

Although the cell position and motion during 1- to 4-cell stages have been reproduced by a coarse-grained model before [10], it remains unknown if the cell morphology can be accurately rebuilt with a simple model as well. Here, we start with reconstructing the cell morphology from 1- to 4-cell stages, by inputting the cell division orientation (i.e., cell volume segregation direction and ratio) measured experimentally (S2 Table). To set up a minimal model in the beginning, cell-cell attraction is ignored in all cell pairs (i.e., *σ*_*i*,*j*_ = 0), resulting in a multicellular system controlled by only cell surface tension, cell-eggshell and cell-cell repulsion, and cell volume constriction. Notably, the simulated embryo morphologies are highly similar to the ones *in vivo* (S1 Fig and S1 Movie). For example, P1 is elongated along the posterior-ventral direction at 3-cell stage and P2 is squeezed along the anterior-posterior direction at 4-cell stage.

**Fig 2:**
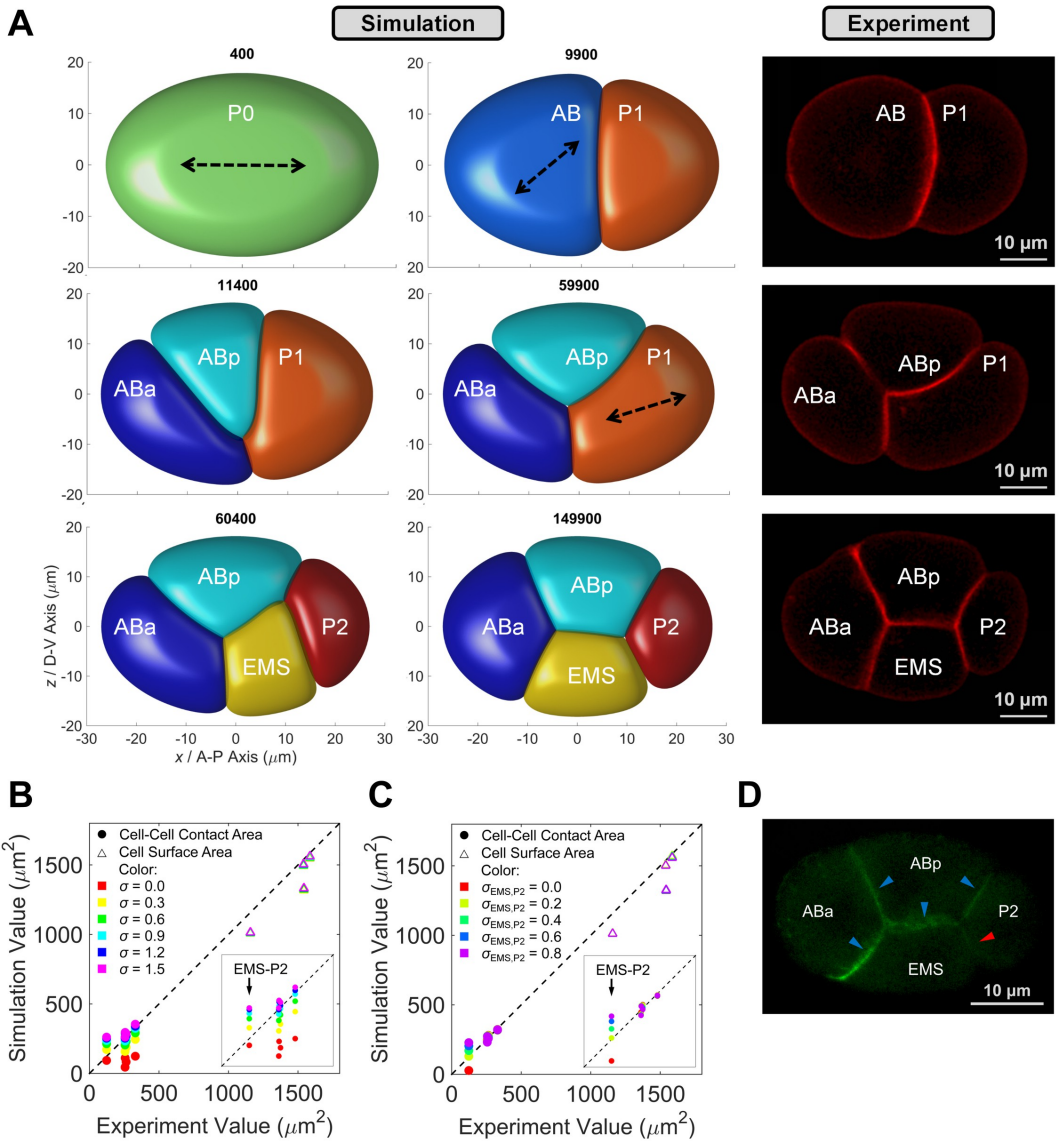
Embryo morphology reconstruction from 1- to 4-cell stages. (A). Comparison of embryo morphology between simulation with cell-cell attraction and experiment from 1- to 4-cell stages (view from *y* / left-right axis). The 1^st^ and 2^nd^ columns, cell-arrangement progression in phase-field simulation; the time point of each embryonic structure is illustrated on its top; dashed arrows, cell division orientation measured by experiment and inputted into simulation; the 3^rd^ column, a live embryo with mCherry fluorescence on cell membrane (strain ZZY0535 [40]); scale bar, 10 μm. (B). Comparison of cell surface area and cell-cell contact area between simulation and experiment at 4-cell stage, with globally symmetric attraction applied on all the cell-cell contacts (*σ* = 0.0, 0.3, 0.6, 0.9, 1.2 and 1.5). Inset, range from 0 to 500 μm^2^ in both coordinates. The quantitative experimental data is obtained at the last moment (time point) of 4-cell stage. (C). Comparison of cell surface area and cell-cell contact area between simulation and experiment at 4-cell stage, with globally symmetric attraction applied on the cell-cell contacts of ABa-ABp, ABa-EMS, ABp-EMS, and ABp-P2 (*σ* = 0.9), and weaker attraction on the contact of EMS-P2 (*σ*_EMS, P2_ = 0.0, 0.2, 0.4, 0.6 and 0.8). Inset, range from 0 to 500 μm^2^ in both coordinates. The average *δ* reaches minimal (≈ 0.06) when *σ*_EMS, P2_ = 0.2. The quantitative experimental data is obtained at the last moment (time point) of 4-cell stage. (D). Distribution of membrane-attached E-cadherin HMR-1 at 4-cell stage, with substantially higher accumulation in the cell-cell contacts of ABa-ABp, ABa-EMS, ABp-EMS, and ABp-P2 (indicated by blue arrows) than that in EMS-P2 contact (indicated by red arrow) (strain LP172 [48]); scale bar, 10 μm.

There are two notable differences between simulation and experiment. First, the interface between AB and P1 at 2-cell stage is slightly protruding toward the larger cell AB in simulation but is in opposite direction in experiment (S1 Fig). This distinction is raised by neglecting the asymmetry of surface tension between cells (i.e., *γ*_AB_ and *γ*_P1_), in other words, the surface tension of AB is plausibly stronger than that of P1 in real embryo so that AB can maintain more spherical *in vivo*, but we treat all the cells with the same level of surface tension (*γ*_AB_ = *γ*_P1_ = 0.25) for simplicity. The increment in *γ* can strengthen a cell’s surface tension and ability to maintain spherical (S2 Fig), and whether AB has stronger surface tension than P1 needs further experimental verification, such as fluorescent imaging on cytoskeleton density or direct measurement on surface tension [43,44]. The second difference is the distinguishable hollows at junction points among cells found from 3- to 4-cell stages. This morphological defect is caused by the cell surface tension, which promotes the sphericity of a cell and then creates space among cells. Nevertheless, those hollows can be eliminated when the cell-cell attraction is introduced, allowing more reliable computation on morphological properties like cell-cell contact area (Figs 2A and S3 and S2 Movie).

### The distribution of adhesive protein in real embryo can be inferred by quantitative comparison of cell surface area and cell-cell contact area between *in silico* and *in vivo*

Given that the intercellular attractive force is essential for modeling the cell morphology accurately (S3 Fig), we next consider this interaction and compare both cell surface area and cell-cell contact area between simulation and experiment. We first set up the global attraction coefficient *σ* = 0.0, 0.3, 0.6, 0.9, 1.2, and 1.5 between all cell pairs and simulate their resultant 4-cell structures respectively, which are permitted to relax until reaching mechanical equilibrium. Here, we use the absolute value of relative error $\delta =\left|\frac{s_{0}-s}{s}\right|$ to estimate the difference between the inferred value *s*_0_ from simulation and the measured value from experiment, and its fluctuation range Δ*δ* during parameter scanning represents the parameter sensitivity [45]. Regarding the scanning range *σ* = 0.0 ~ 1.5, the cell surface area depends little on global attraction because it’s dominantly determined by a cell’s prescribed volume (Δ*δ* < 0.02 for all cells), while the cell-cell contact area substantially rises with the increase of global attraction (Δ*δ* > 0.87 for all contacts) (Fig 2B and S7 Table). When *σ* = 0.9, the average *δ* reaches minimal and all the surfaces and interfaces acquire an area that moderately fits the experimentally measured value (*δ* < 0.14), except the contact area between EMS and P2 (*δ* > 0.91). The simulated area of EMS-P2 contact is near twice the actual value, indicating a weaker attraction between them. Surprisingly, a recent study reported that the accumulation of E-cadherin HMR-1 is significantly lower in EMS-P2 contact than the others at 4-cell stage, validating the model prediction (Fig 2D) [10].

Considering the oversized area of EMS-P2 contact, we further screen a smaller attraction coefficient *σ*_EMS, P2_ = 0.0, 0.2, 0.4, 0.6, and 0.8 and eventually obtain the optimal combination *σ*_EMS, P2_ = 0.2 and *σ* = 0.9 for others, achieving an area of EMS-P2 contact close to its actual value (*δ* < 0.14 for all contacts) (Fig 2C and S8 Table). The cell-cell attraction pattern is directly derived by quantitative comparison of cell surface area and cell-cell contact area between *in silico* and *in vivo*, showing the model’s power in capturing cell morphology and inferring biophysical properties without the extra help of experiment. Moreover, compared to the coarse-grained model used before, the phase field model can facilitate the biological research involved with the cell interface. For example, our computational system can be utilized to study the physical contact area between cells, which is usually coupled with signaling transduction for precise fate specification and division regulation [17,40,46,47].

### A self-determined binarized cell-cell attraction matrix is used for later developmental stages

Using the phase field model established with *in vivo* data from 1- to 4-cell stages, we next attempt to compute the morphogenetic procedures at 6-, 7- and 8-cell stages, to further test the model performance and explore the underlying strategies and principles for embryonic morphogenesis. Specifically, the cell-cell attraction has been proved to influence the cell-arrangement pattern at 4-cell stage substantially [10]. However, its function at later stages is unclear. Since this mechanical parameter is hard to fully measure by experiment, especially when the cell number is large, we introduce two simple assumptions to set the cell-cell attraction matrix automatically and make the multi-stage simulation self-driven.

First, the cell-cell attraction matrix is binarized. As this force exhibits relatively strong or weak intensity at 4-cell stage (Fig 2B–2D), for simplicity, we use the *σ* values fitted to represent those two states, i.e., *σ*_S_ = 0.9 and *σ*_W_ = 0.2. This approximation reduces the dimension of cell-cell attraction matrix and limits the possibility of a developmental path, to achieve an affordable computational cost. Second, the attraction between non-sister cells and between sister cells is regarded as relatively strong and weak respectively. It was previously found that some sister cells (e.g., ABpl and ABpr) can be separated during collective motion, suggesting that their attraction is not enough to keep them connected continuously, even though they are initially adjacent due to their sisterhood [34]. This weak interaction is likely caused by the postponed recovery of membrane-attached proteins (e.g., HMR-1) when the membrane grows and ingresses during cytokinesis (S4 Fig). Besides, the membrane shared by a dividing cell and its neighbors is hardly influenced so the protein accumulation seems to be intact.

Apart from the autogenic cell-cell attraction matrix default above, another value assignment on *σ* is also permitted (hereafter referred to as “attraction motif”) based on experimental measurement or known regulation, such as the low accumulation of HMR-1 in EMS-P2 contact (Fig 2D).

### All the conserved cell-cell contacts and non-contacts at 6-, 7- and 8-cell stages can be established under proper cell-cell attraction matrix

**Fig 3:**
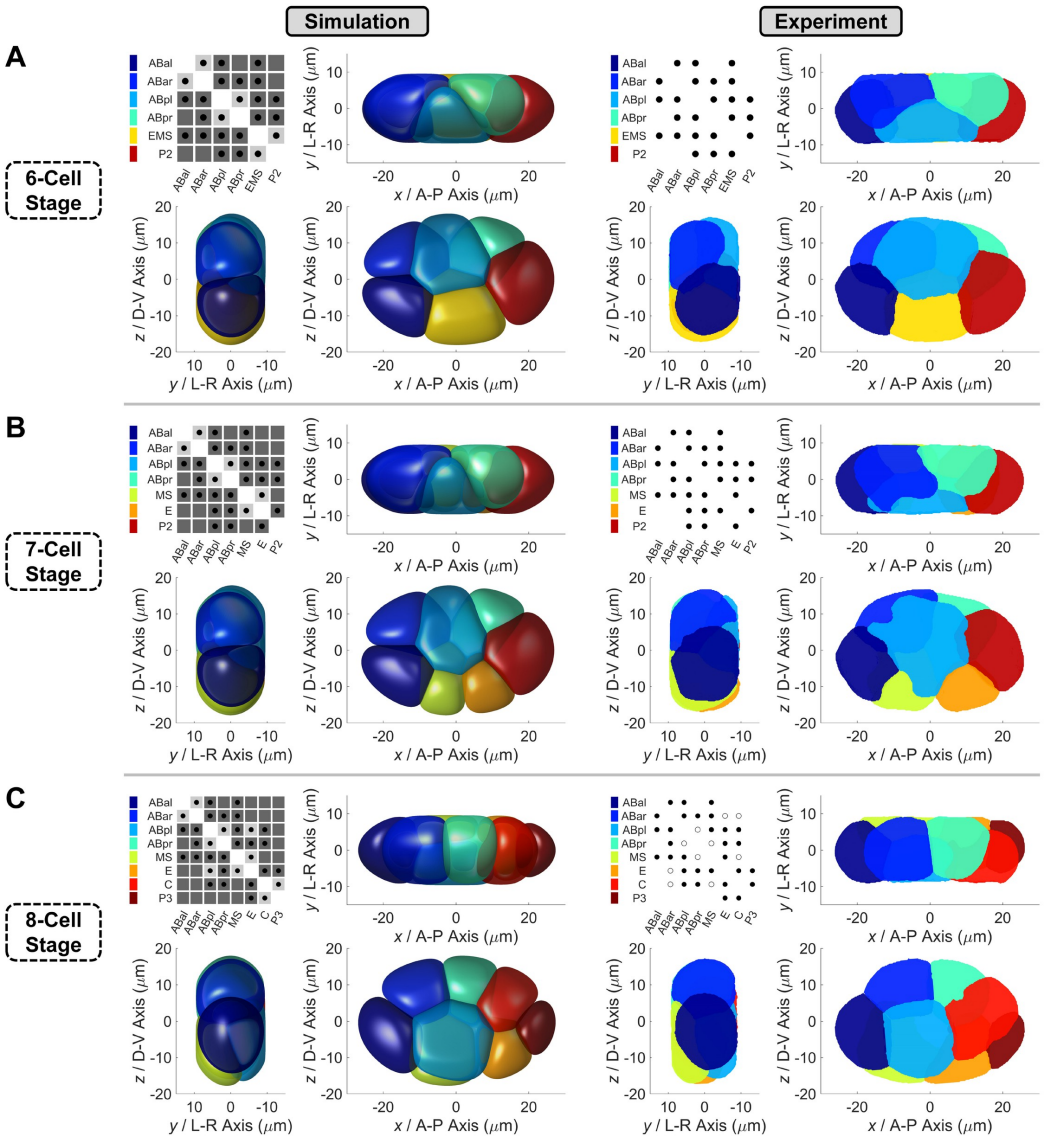
Comparison of embryo morphology between simulation and experiment from 6- to 8-cell stages. (A). The upper panel, embryo morphology at the last moment of 6-cell stage. (B). The middle panel, embryo morphology at the last moment of 7-cell stage. (C). The lower panel, embryo morphology at the last moment of 8-cell stage (with attraction motif on ABpl-E contact, i.e., *σ*_ABpl, E_ = *σ*_W_). In each panel, embryo morphology in simulation and experiment are respectively illustrated on the left and right in three orthogonal observation directions, while a cell-cell contact map is placed in their top left corners. About the map in simulation, dark and light gray shades denote relatively strong (*σ* = *σ*_S_) and weak (*σ* = *σ*_W_) attraction respectively, while black dots represent the contacted cell pairs. About the map in experiment, black dots represent the conserved contacted cell pairs, while empty circles represent the unconserved contacted cell pairs [34]. The relationship between cell identity and color is listed next to the contact maps. The quantitative experimental data is obtained at the last moment (time point) of each stage.

**Fig 4:**
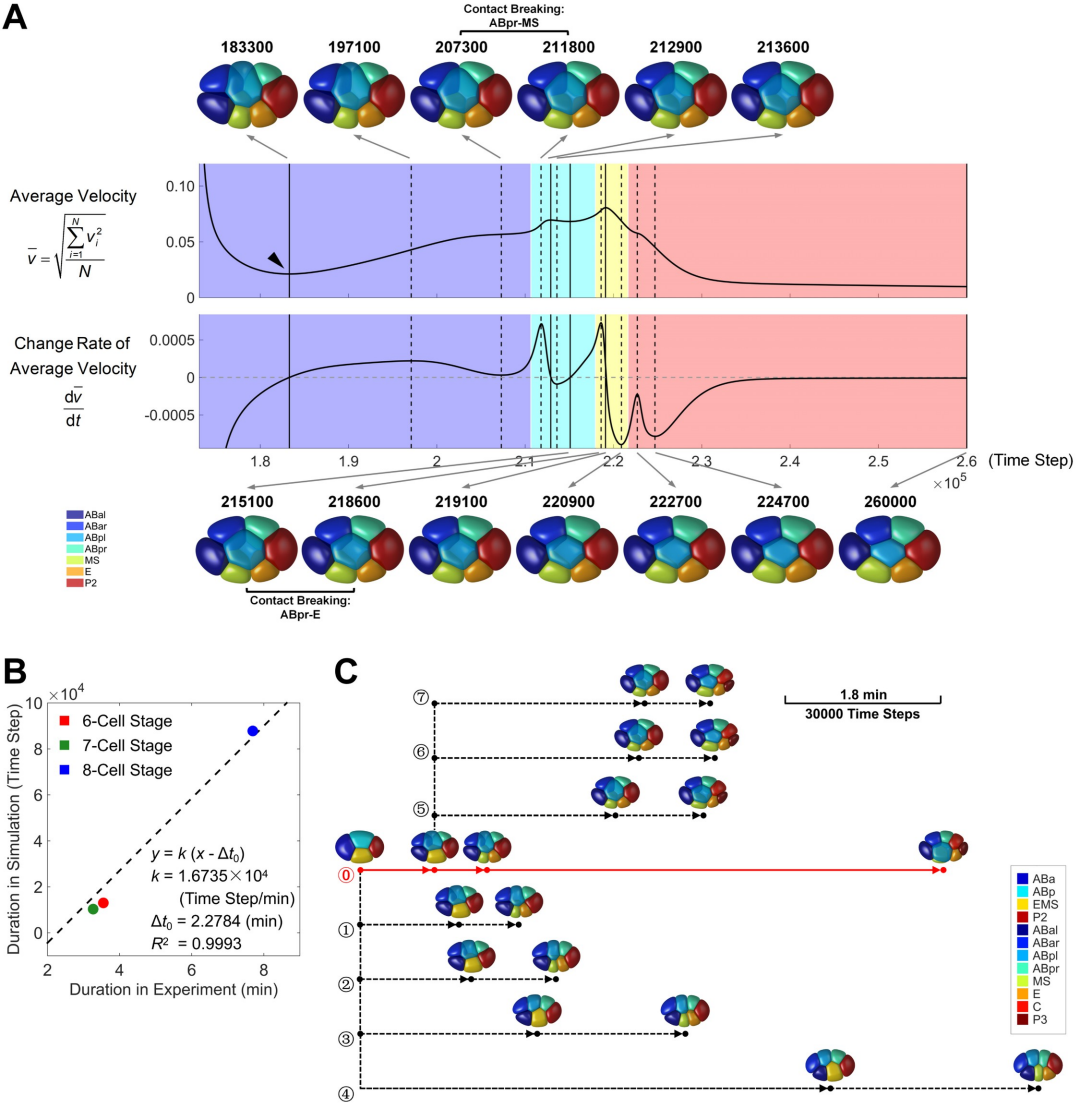
The role of timely cell division to protect the 3D embryo structure and cell-cell contact map. (A). Morphological evolution during 7-cell stage, with simulation time long enough for the whole system to reach mechanical equilibrium. The curves of average velocity (upper) and its change rate (lower) are illustrated side by side. The solid and dashed vertical black lines denote the extreme points in the two curves respectively, while the 3D structures at those time points are illustrated on top and bottom, pointed by gray arrows originating from their corresponding lines. The last structure in the bottom right is the system’s terminal state approaching mechanical equilibrium. The change of cell-cell contact map is illustrated by different colors in the background, while the detail is written between two consecutive structures. The time point of the first quasi-steady state is indicated by a black triangle. The relationship between cell identity and color is listed in the bottom left corner. (B). Linear fitting of time scale between simulation and experiment systems. The durations in simulation are obtained from the quasi-steady states (Figs 4A, S6, and S7B), while the ones in experiment are obtained from 222 wild-type embryos in a previous dataset [2]. The intercept is predetermined as −Δ*t*_0_ = −2.2784 min, obtained from 4 wild-type embryos in another dataset [34]. The time step in computation is consistently set as *h* = 0.1 for all stages. (C). An evolutionary tree composed of 8 developmental paths diversified by different cell division timing. The branch of the normal developmental path is plotted with a solid red line while the ones with disturbed cell division timing are plotted with dashed black lines. The cell division timing is denoted with a solid point; the perturbed cell division timing is set at the critical time points (i.e., extreme points) in the curves of average velocity and its change rate (Figs 4A and S6), and only the ones with developmental path differentiated from the others are plotted. The final state is determined by the first and second quasi-steady states for 7- and 8-cell stages respectively, and the 3D structures at the time points with cell divisions activated or in the end are illustrated near the corresponding nodes. A scale bar representing the *in silico* and *in vivo* time scales is placed in the top right corner. The terminal embryo morphology and cell-cell contact map of branches ⓪ ~ ⑦ can be found in S9 Fig. The relationship between cell identity and color is listed in the bottom right corner.

Using the phase field model with cell-cell attraction matrix, the morphogenetic dynamics from 6- to 8-cell stages are computed according to the division order and division orientation measured experimentally (Figs 3A–3C, 4A, S5, S6, S7A and S7B and S2 Table and S3–S6 Movies). To inspect the motion state of the whole embryo, we calculate the root-mean-square velocity of all cells inside the eggshell $\bar{v}=\sqrt{\frac{\sum_{i=1}^{N}v_{i}^{2}}{N}}$ as the system’s average velocity, where *N* is the cell number and *v*_*i*_ is the velocity of cell *i*’s mass center, which is derived by its phase field through $r_{c}=\frac{\int_{\Omega }r\phi_{i}^{t}dr}{\int_{\Omega }\phi_{i}^{t}dr}$. Apart, we define a quasi-steady state in the $\bar{v}-t$ curve as the time point *t*_*q*_ where its first derivative is zero (i.e., ${\frac{d\bar{v}}{dt}|}_{t=t_{q}}=0$) and its second derivative is positive (i.e., ${\frac{d^{2}\bar{v}}{dt^{2}}|}_{t=t_{q}}>0$), which means the average velocity reaches a local minimum over time. Given that a full relaxation after cell division is observed at all the 6-, 7- and 8-cell stages *in vivo* (S8 Fig), we activate the cell division at the time point when the embryo reaches a quasi-steady state with temporally minimal kinetic energy, i.e. when the embryo moves the slowest. It should be pointed out that in simulation, both the 6- and 7-cell stages end at their first quasi-steady state and the 8-cell stage ends at its second quasi-steady state, because the duration of 8-cell stage is over twice of those of 6- and 7-cell stages in a real embryo (Figs 4A, S6, S7A and S7B) [2]. Hereafter, we use the cell-cell contact relationships reproducible among individuals as the ground truth to verify the simulation results. For a specific stage, a cell pair is defined as “conserved contact” if they contact in all embryo samples (*N*_contact_ = 4); if they do not contact in any embryo sample, it is defined as “conserved non-contact” (*N*_contact_ = 0); the other cases are “unconserved contact” (1 ≤ *N*_contact_ ≤ 3) (S4–S6 Tables). This ternary cell-cell contact map captures the basic morphological features of a cell and has potential information on which contact/non-contact is significant and which one matters little for *C*. *elegans* embryogenesis. For simplicity, we only compare the conserved contacts and non-contacts between *in silico* and *in vivo*, but not the contact area, for that fitting this quantitative property requires massive work in both simulation and experiment.

For both 6- and 7-cell stages, all the conserved cell-cell contacts and non-contacts are reproduced using the cell-cell attraction matrix default. Besides, the cell location and cell morphology are highly similar to the ones *in vivo*, indicating that the model successfully recaptures the physical state of each cell as well as the mechanical interactions between them (Fig 3A and 3B and S3 and S4 Movies). The ring-like structure at 7-cell stage, in which ABpl is surrounded by the other 6 cells, was reported to play a pivot role in polarity redistribution and axis establishment (Fig 3B) [49]. Our simulation shows that this geometric pattern is set up by cooperative cell division orientations of ABa, ABp, and EMS, which together make ABpl the embryo’s center. This might be associated with ABpl’s unique behavior, including the most severe deformation (defined as a cell’s dimensionless surface-to-volume ratio) and the longest travel distance (defined as the length of the road a cell passes) [2,50,51].

For 8-cell stage, the model fails to reproduce the morphogenetic dynamics observed *in vivo* when the default cell-cell attraction matrix is applied. Although the embryo morphology resembles the real one initially, ABpl ingresses inward along the L-R axis instead of migrating in the anterior-ventral direction, leading to a flattened structure in the AP-DV plane with the conserved ABpr-MS contact broken (S5 and S7A Figs and S5 Movie). A recent experimental study reported the significantly higher E-cadherin HMR-1 accumulation in ABpl’s anterior contacts than those in the posterior [49], however, the relatively strong attraction in ABpl-E and ABpl-C contacts (i.e., *σ*_ABpl, E_ = *σ*_ABpl, C_ = *σ*_S_) disobeys this observation (S5 Fig). Thus, we respectively add the attraction motif onto these contacts to mimic the weak cell-cell adhesion *in vivo*, namely, *σ*_ABpl, E_ = *σ*_W_ and *σ*_ABpl, C_ = *σ*_W_. Intriguingly, the remarkable movement of ABpl and a persistent 3D embryo structure are rescued by the ABpl-E motif but not by the ABpl-C motif (Figs 3C and S7B and S6 Movie), indicating that a weak attraction in ABpl’s posterior is essential for its migration in anteroventral direction. Besides, the cell-cell contact map is in agreement with the one conserved in real embryos, which however failed to be fully recaptured in a previous study using optimized coarse-grained models [22]. After weakening the attractive force between ABpl and E, the positions and contacts of the 8 cells exhibit mirror symmetry about the AP-DV plane, concerning both the geometric and mechanical topologies. In brief, ABal, ABar, ABpr, C, P3, and E cells are sequentially located near the AP-DV plane, while ABpl and MS are surrounded by them and evenly distributed on both sides of the AP-DV plane (Fig 3C). This spatial symmetry theoretically explains how the regulated low accumulation of E-cadherin HMR-1 or weak attraction in ABpl’s posterior, especially in the ABpl-E contact, promotes the structural stability at 8-cell stage.

### The cell division timing determined by quasi-steady state is in line with the experimental measurements

In simulations for 6- to 8-cell stages, we design an autonomic time-selecting rule that the cell division takes place when the embryo reaches a quasi-steady state with temporally minimal kinetic energy (Figs 4A, S6, S7A and S7B). The rule is hypothesized based on the relaxation process observed empirically and independent of the cell division timing *in vivo*, which however successfully generates the cell morphologies in favorable agreement with the experimental ones (Figs 3A–3C and S8). To validate whether our phase field model can characterize the real time scale and explore the biophysical meaning of this rule, we first align the time scale of the model (time step) to the one in reality (min). A proportional linear fitting is performed on the duration of all stages between simulation and experiment (least square method), revealing a conversion ratio *k* = 1.6735×10^4^ time step/min and considerable goodness of fit *R*^2^ = 0.9993 (Fig 4B). An intercept is predetermined as −Δ*t*_0_ = −2.2784 min, which is the experimental duration between cell nucleus separation and cell membrane segregation, considering that the detailed process during cytokinesis is ignored in simulation. The relation between the lengths of the three stages, namely, Δ*t*_7−Cell_<Δ*t*_6−Cell_<Δ*t*_8−Cell_, which is determined by the cell cycle or division timing of EMS, P2, and AB4 cells and is delicately regulated by multiple genes [2], is also recaptured by the time-selecting rule. Sufficient time is essential for the 8-cell stage, which allows the long-range migration of ABpl as well as the global rotation of embryo (S7B Fig and S6 Movie). It is noteworthy that the accelerated motion at the late 8-cell stage observed *in vivo* is not reproduced in our simulation, implying that other detailed mechanisms need to be added to describe the real system for higher accuracy, such as the lamellipodia, protrusion, and filopodia that have been reported to drive ABpl’s anterior-ventral migration [50].

The time-selecting rule based on quasi-steady state has biophysical significance as follows. First, activating cell division at the least motional state can minimize the structural variation raised by intrinsic variation in cell division timing. Second, according to simulations for 6- and 7-cell stages, the embryo under compression collapses into the defective flattened structure if the next cell division is postponed, revealing less robustness (Figs 4A and S6 and S3 and S4 Movies). This explains why the cell divisions before gastrulation onset are programmed in distinct orders with intervals always larger than 3 min [2,52]. Given that the phase field model could describe the morphogenetic dynamics *in vivo* regarding both space and time, next we systematically investigate the effect of three physical factors, i.e., cell division timing, cell division orientation, cell-cell attraction matrix, on the embryonic morphogenesis, and reveal their roles in depth from a morphological perspective.

### A timely cell division can protect the established 3D embryo structure and cell-cell contact map

The cell division timing in *C*. *elegans* early embryogenesis is coordinated by a lot of mechanisms, such as DNA replication checkpoint [53,54], cell volume redistribution [55,56], and cell-cell signaling [57,58], and was proposed to affect the cell migration in theoretical studies using coarse-grained model [20,21]. In the phase-field simulations for both 6- and 7-cell stages, a novel phenomenon termed “structural planarization” is found when cell division timing is postponed, in which some cell-cell contacts are broken and all the cells are located within the AP-DV plane eventually (Figs 4A and S6).

To systematically investigate how cell division timing influences the morphogenetic dynamics, we disturb the division timing of EMS and P2 and construct the evolutionary tree from 6- to 8-cell stages (Fig 4C). We independently initiate the cell division at the critical time points (i.e., extreme points) in the curves of average velocity and its change rate at 6- and 7-cell stages (Figs 4A and S6), and then push forward the simulation with the same cell division orientations and cell-cell attraction matrices. A total of 8 developmental paths are identified based on their different cell-cell contact maps in the end (Figs 4C and S9). Interestingly, if EMS divides a bit later (≥ 5500 time steps) than the time point of the first quasi-steady state, the conserved ABar-ABpr contact would be broken immediately; for the delay in P2 division (≥ 24000 time steps), the terminal embryo structure fails to maintain three-dimensional with the conserved ABpr-MS contact broken, although the ABpl-E motif is added. We notice that for all the 6-, 7- and 8-cell stages, a conserved cell-cell contact or non-contact between two cells is initially established by the last cell division and persistently remained until their divisions, no matter in simulation (with correct cell division timing) or experiment. Provided that a precise and robust cell-cell contact map serves the cellular interaction, such as biochemical and mechanical signaling transduction [11,16,17,46], a timely cell division protects such important physical contacts/non-contacts while maintaining the 3D embryo structure.

### Both volume segregation direction and ratio during cytokinesis have selective impact on the developmental path

**Fig 5:**
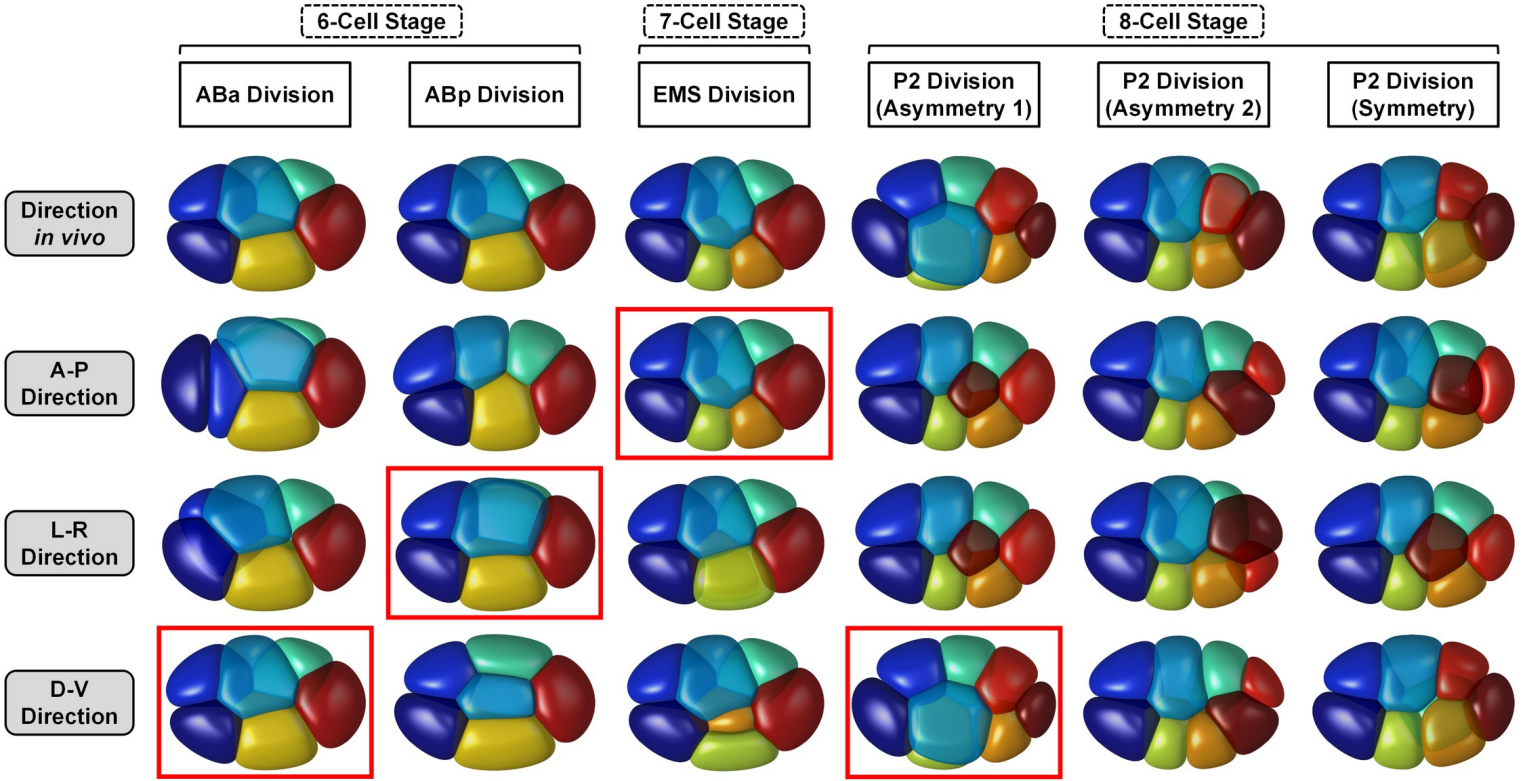
The selective impact of cell division orientation on the developmental path. Simulation for 6- to 8-cell stages with cell division orientations aligned to the three orthogonal body axes. All the division orientations of ABa (1^st^ column), ABp (2^nd^ column), EMS (3^rd^ column), and P2 (4^th^, 5^th^, 6^th^ columns with different volume segregation ratio) are set along the experimental (1^st^ row), anterior-posterior (2^nd^ row; *x* / A-P), left-right (3^rd^ row; *y* / L-R), and dorsal-ventral (4^th^ row; *z* / D-V) directions, respectively. The 3D structures whose cell-cell contact map is the same as the one simulated with experimental orientation, are highlighted with red rectangles.

Here, we define “cell division orientation” as a combination of cell volume segregation direction and ratio during cytokinesis, which is mainly regulated by cell polarization [15,59], redirected myosin flow [11,60], and intercellular signaling transduction [16,46] in *C*. *elegans* early embryogenesis. A previous coarse-grained study has reported the selective impact of cell division orientation on an embryo’s structural evolution, by simply placing two interactive points along a predetermined axis to model cytokinesis [21]. As the phase field model can simulate the whole cell body, we further study in detail how the two components, volume segregation direction and ratio, impact the morphogenesis. To construct a simplified scenario, we align the division orientations of ABa, ABp, EMS, and P2 onto the three orthogonal body axes, which are identified to be parallel to the D-V, L-R, A-P, and D-V axes, respectively (Fig 5). In simulation, all the divisions of ABa, ABp, and EMS are nearly symmetric (S2 Table), and only the trials with their dominant directions can rebuild the embryo morphology and cell-cell contact map that resemble the real ones. Moreover, for the two daughters of P2, C acquires a volume around twice that of P3 (S2 Table), thus, there are a total of 3×3 = 9 parameter combinations (i.e., direction along A-P, L-R or D-V axis, *V*_C_:*V*_P3_ ≈ 2:1, 1:1 or 1:2). Interestingly, the volume segregation direction of P3 must be along the D-V axis and the larger cell (C) must be placed on top, otherwise, an allosteric 3D structure will appear (Fig 5). These results suggest that not only the division direction but also the volume segregation ratio is significant for the stereotypic morphogenesis in *C*. *elegans* embryo.

Provided that the P2 division serves as a structural regulator for the 8-cell stage regarding both its volume segregation direction and ratio, we try to search the possible mechanism that specifically controls it *in vivo*. We screen 758 genes (≥ 2 replicates for each; RNA-interference) with a wild-type reference formed by 222 embryos, using the nucleus-based data and method provided in [2]. Notably, we identify a gene, *pad-1* (abbreviation of patterning defective 1), whose corresponding RNAi-treated embryos have normal structure at 7-cell stage but significantly deviated division orientation in P2, and following global misarrangement in cell positions at 8-cell stage (S10 Fig). This morphogenetic phenotype occurs much earlier than the time proposed before (i.e., around 26- to 28-cell stages) [61] and matches the regulatory impact of P2 division predicted in theory, suggesting *pad-1* as a possible genetic factor that influences the developmental path by controlling P2 division. Given that the detailed function of *pad-1* is elusive, more *in vivo* experiments are needed to further explore how it functions in P2 division and whether the morphogenetic chaos in the RNAi experiment is attributed to the erroneous P2 division.

### The cell-cell attraction matrix provides high-dimensional diversity and regulatory potential for the developmental paths

**Fig 6:**
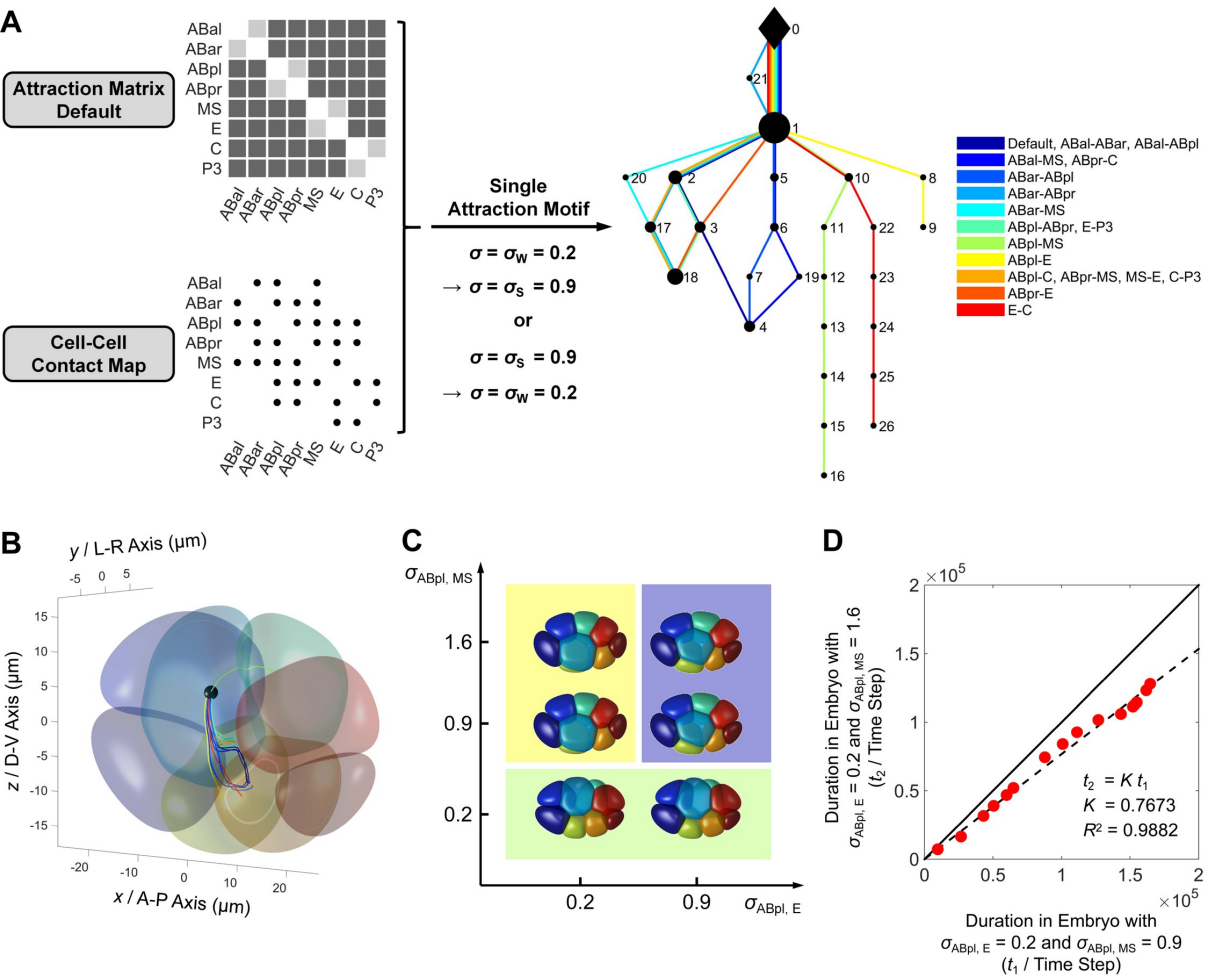
High-dimensional diversity and regulatory potential of developmental paths provided by cell-cell attraction matrix and extra attraction motifs. (A). A total of 17 single attraction motifs (*σ* = *σ*_W_ = 0.2 → *σ* = *σ*_S_ = 0.9 or *σ* = *σ*_S_ = 0.9 → *σ* = *σ*_W_ = 0.2) based on the cell-cell contact map at 8-cell stage (bottom left corner) are applied on the cell-cell attraction matrix default (top left corner) independently for simulation. The evolutionary sequence of cell-cell contact map is used to classify the developmental paths, illustrated on the right. Each color represents a unique developmental path originating from top and ending in bottom, with its corresponding attraction motif(s) indicated in the legend; the black diamond denotes the cell-cell contact map at the last moment of 7-cell stage (Topology 0 in S12 Fig); the black points denote the different cell-cell contact maps at 8-cell stage (Topologies 1 ~ 26 in S12 Fig), with a size positively correlated to the number of developmental paths passing through. (B). The migration trajectory of ABpl’s mass center when the 17 attraction motifs are added. The initial 8-cell structure is illustrated semi-transparently for visual comparison. The colors representing different cell identities are the same as those used in Fig 3C. The colors representing trajectories with different attraction motifs are the same as those used in Fig 6A. (C). The three types of developmental paths differentiated when different combinations of *σ*_ABpl, E_ and *σ*_ABpl, MS_ are applied (*σ*_ABpl, E_ = 0.2, 0.9 and *σ*_ABpl, MS_ = 0.2, 0.9, 1.6), highlighted with light yellow, purple, and green backgrounds. The 3D structures at their second quasi-steady states are illustrated. (D). The match of migration rate between simulated embryos with *σ*_ABpl, E_ = *σ*_W_ = 0.2, *σ*_ABpl, MS_ = *σ*_S_ = 0.9 and *σ*_ABpl, E_ = *σ*_W_ = 0.2, *σ*_ABpl, MS_ = *σ*’_S_ = 1.6. A total of 16 critical time points selected according to the extreme points in the curves of average velocity and its change rate are used to compare the migration rate in the two simulations (S13 Fig and S10 Table).

The cell-cell attraction has been proposed to influence the cell-arrangement pattern at 4-cell stage [10], nevertheless, its specific role and regulation at the later stages remain unclear. Considering that the structural evolution of 6- and 7-cell stages can be reproduced by the cell-cell attraction matrix default while the 8-cell stage requires an attraction motif on ABpl-E contact (Figs 3A–3C and S5), we choose the 8-cell stage to systematically explore the effect of cell-cell attraction matrix on the developmental path. For simplicity, a single attraction motif is added onto the 17 contacted cell pairs independently, from weak to strong (*σ* = *σ*_W_ → *σ* = *σ*_S_) or on the contrary (*σ* = *σ*_S_ → *σ* = *σ*_W_). All the simulations are run until time point 350000 (S11A Fig), then the ones with 3D structures (i.e., ABpl-MS, ABpl-E, and C-E motifs) are extended to time point 450000 (S11B Fig). The evolutionary sequence of cell-cell contact map is used to classify the developmental paths. The simulations reveal 11 types of developmental paths, 5 types of terminal structures, and 26 types of cell-cell contact maps (Figs 6A and 6B and S12). Interestingly, some paths can merge with or separate from each other, for instance, the terminal structure of cell-cell attraction matrix default can be reached by the ones with attraction motif on ABal-ABar, ABal-ABpl, ABal-MS, ABar-ABpl, or ABpr-C contact (Topology 4 in Figs 6A and S11A), through different evolutionary sequences. The decentralized pattern in Fig 6A indicates that the developmental path can be highly diversified by different cell-cell attraction matrices, which could be achieved by regulations like the weakening of adhesive protein accumulation in ABpl’s posterior [49].

### The weak attraction in ABpl-E contact and strong attraction in ABpl-MS contact serve as stabilizer and accelerator respectively

About the 5 types of terminal structures in the scanning of attraction motifs, two fail to reach three-dimensional (Topologies 4 and 18 in Figs 6A and S11A), and three succeed (Topologies 9, 16, and 26 in Figs 6A and S11A). Among them, the ABpl-E motif (i.e., the low accumulation of E-cadherin HMR-1 measured *in vivo*) protects the 3D embryo structure most persistently, which has the longest interval with an unchanged cell-cell contact map (duration = 150500 time steps *in silico* ≈ 8.99 min *in vivo*) (S9 Table), making it an excellent stabilizer for *C*. *elegans* embryo morphology at 8-cell stage.

A previous study discovered the dense protrusions in the anteroventral surface of ABpl, which establish a strong bonding and dragging to the MS cell below [50]. To explore the potential function of such an actively strengthened attraction, we introduce *σ*’_S_ = 1.6 to represent an even stronger state than *σ*_S_ = 0.9, and conduct simulations under *σ*_ABpl, E_ = *σ*_W_, *σ*_S_ (0.2, 0.9) and *σ*_ABpl, MS_ = *σ*_W_, *σ*_S_, *σ*’_S_ (0.2, 0.9, 1.6). There are three distinct types of developmental paths regarding the 8-cell structures at their second quasi-steady states (Fig 6C). The developmental path *in vivo* can be reproduced only when the ABpl-E attraction is weak (*σ*_ABpl, E_ = *σ*_W_ = 0.2) and the ABpl-MS attraction is over a threshold (*σ*_ABpl, MS_ ≥ *σ*_S_ = 0.9). Compared to the attraction default at ABpl-MS contact (i.e., *σ*_ABpl, MS_ = *σ*_S_ = 0.9), increasing its attraction intensity can almost linearly shorten the time scale of the morphogenetic process (Fig 6D). By tracking the extreme points in the curves of average velocity and its change rate and comparing the durations cost to reach them, we find that the embryo with *σ*_ABpl, MS_ = *σ*’_S_ = 1.6 grows around a quarter faster than the one with *σ*_ABpl, MS_ = *σ*_S_ = 0.9. In other words, the protrusions observed in the ABpl’s anteroventral surface *in vivo* could accelerate the morphogenesis at 8-cell stage.

### The structural planarization is a common defective phenotype in compressed embryo, which could be induced by incorrect cell division timing, cell division orientation, and cell-cell attraction matrix

A characteristic tendency of structural planarization into the AP-DV plane exists at all 6-, 7- and 8-cell stages if the system is allowed to relax with enough time (Figs 4A, S6, and S7A). Apart, it occurs frequently when we input incorrect cell division timing (Fig 4C), cell division orientation (Fig 5), and cell-cell attraction matrix (S11A Fig). One possibility for this phenotype is that the embryos imaged by confocal microscopy are compressed along the L-R axis for better imaging quality, where the compression ratio is roughly 0.5 (Fig 1C) [34–36]. To verify this hypothesis, we generate an uncompressed eggshell with a major axis and total volume as same as the ones of the compressed eggshell. Simulations output sustainable 3D structures with a correct cell-cell contact map at all stages, no more dependent on the timely cell divisions or the ABpl-E motif (S14A and S14B, S15, S16, S17A and S17B Figs and S7–S10 Movies).

Next, we attempt to check if the “structural planarization” phenotype takes place *in vivo* when the developmental programs are disturbed. Firstly, 222 wild-type embryos from our previous work are used to estimate the normal embryo width at the last time points of 6-, 7- and 8-cell stages, by calculating the maximum distance in *y* / L-R direction among all the cell nuclei (S18A–S18C Fig) [2]. The nucleus-based dataset is utilized for its large sample size, which permits reliable statistical comparison. Secondly, 3 additional wild-type embryos were cultured under the same experimental protocol [38], and their P2 cells are ablated by laser for 90 seconds during 4-cell stage ([40]; see Materials and Methods), to mimic the delayed cell division in simulation (Fig 4A). Consequently, the cell cycle of P2 arrests and the duration of 7-cell stage is lengthened from 3.27 ± 0.87 min to 10.33 ± 0.81 min, while the duration of 6-cell stage remains still. Provided the laser ablation on P2, the embryo width at the extended 7-cell stage, but not the 6-cell stage, is significantly narrower than the normal value (*p* ≈ 0.00887, one-tailed Wilcoxon rank-sum test) (S18A and S18B Fig), suggesting that the “structural planarization” phenotype do exist *in vivo* (Fig 4A and 4C). It is noteworthy that, we cannot exclude the possibility that the other biological process beyond the P2 division is also affected by laser ablation and contributes to the phenotype, such as the mechanical properties of P2 and the Wnt signaling from P2 to EMS [16].

To sum up, a tendency of structural planarization with contacts broken is caused by external compression and is easy to appear under perturbation. It was previously proposed that lateral compression exists *in utero*, in particular to the starved or old individuals [1,60]. Here we propose the cell division timing, cell division orientation, and cell-cell attraction matrix as three novel physical factors that provide robustness against mechanical perturbation in *C*. *elegans* early embryogenesis.

## Discussion

How metazoan develops from a single zygote into a stereotypic morphological pattern has been a fascinating problem for decades. Little is known about the fundamental strategies and principles. Reliable computational models capable of simulating real systems and permitting large-scale virtual experiments are needed. In this work, we built a phase field model with the help of *in vivo* imaging data of *C*. *elegans* embryos, to simulate the mechanical interactions and constrictions at the cellular level, including cell surface tension, cell-eggshell and cell-cell repulsion, cell-cell attraction, and cell volume constriction. The model successfully reproduced the structural evolution from 1- to 8-cell stages (S11 Movie), and demonstrated the utility in inferring key biophysical factors in morphogenesis like cell adhesion. Simulations with variable parameters indicated that a delicate program of cell division timing, cell division orientation, and cell-cell attraction matrix is crucial for precise and robust morphogenesis, in particular for establishing the correct cell-cell contact map, which serves as the physical basis for many biological regulations.

We predicted and experimentally verified that a phenotype termed “structural planarization” occurs easily when the embryo is compressed and subject to perturbation. This spatially defective phenotype is caused by external compression on the embryo which has been a widely used condition for imaging experiments. Therefore, cautions should be taken in drawing general conclusions from compressed embryos. The morphological phenotype prediction on perturbed embryos, together with the step-by-step reconstruction of embryonic morphology and inference of cell-cell attraction intensity, not only demonstrates the phase-field method’s capability in simulating cell shape and cell-cell mechanical interaction *in vivo* but also serves as an example of how to build reliable and predictive simulators for other multicellular organisms.

Despite the initial success of the model, problems remain and further improvements are possible in the future. First, as the cell number increases, it becomes computationally expensive, especially when more parameters are being explored. This could possibly be resolved by constructing a solution landscape to identify all possibilities [62]. Another option is to reduce the model complexity, for example, to reach a multi-particle or coarse-grained model properly [9,10,18,19], which might also be able to produce some biological findings in this work. Second, a more precise value assignment on all physical parameters, instead of a uniform or binarized value used in this study, can fit the experiment even better, in particular to the fine morphological details like the curvature and area of a contact interface between cells (Figs 2B and 2C and S2). This might be achieved by quantitative comparison between *in silico* and *in vivo*, or by the known regulations or with new measurements [44,49]. Of course, this is in the direction of increasing model complexity. Third, the later developmental stage may involve more subtle and complicated regulations such as contraction and polarization [13,29]. Thus, additional biochemical or biophysical mechanisms would be required for the model. Last but not least, a previous comparative study revealed that the outputs of simulation on the same multicellular system would vary from model to model [63]. For example, the coarse-grained model and phase field model treat a cell as a single particle and a 3D field respectively, leading to different levels of simulation accuracy and computational cost. How to choose a model for a specific biophysical scene with enough accuracy and low cost becomes a practical problem and merits further study [64].

The phase-field approach in this paper can be applied to reconstruct other multicellular systems with cell morphology data, such as *Arabidopsis thaliana* and *Drosophila melanogaster* [65,66]. Besides, many artificial multicellular systems were constructed recently, with practical functions such as self-organization, self-repairing, and autonomic motion [67,68]. A solid model that is capable of describing cell-cell interactions, cell deformation, and cell motion accurately could facilitate the design and study of those synthetic systems. Thus, our computational framework could shed light on the simulations of both natural and artificial multicellular systems.

## Materials and methods

### Data collection of cell-resolved developmental properties from previous *in vivo* imaging experiments

Here, we collect the imaging data as well as its processed results (i.e., cell segmentation, cell tracking, and cell lineaging) of three groups of *C*. *elegans* wild-type embryos from previous datasets, for different purposes of usage (S1 Table) [2,34]. The first group is an embryo with a GFP marker ubiquitously expressed and localized in cell nuclei, which was imaged since 1-cell stage. The second group is formed by 13 embryos with only a nucleus marker (GFP) imaged since 4-cell stage. The third group consists of 4 embryos with both membrane (mCherry) and nucleus (GFP) markers imaged since 4-cell stage, providing 3D time-lapse cell morphology information. All the embryos used in this work were cultured at room temperature within 20 ~ 22°C and imaged using a confocal microscope with a temporal resolution of ~1.5 minutes/frame. Then the location of the cell nucleus is measured as the center of the histone-labeled GFP fluorescence, which is used for following tracking and lineaging with software StarryNite and AceTree (Fig 1A) [35]. The detailed usages of the 18 embryos are listed in S1 Table.

Using the embryo data obtained above, cell division orientation, cell volume, and eggshell shape are quantified and inputted into the simulation as predetermined parameter values (Fig 1B and S2 Table). Note that as cell morphology is obtained since the last co-existence moments of ABa, ABp, EMS, and P2 (i.e., 4-cell stage), the volume of AB is calculated as the sum of volumes of its daughters ABa and ABp; the volume of P1 is calculated as the sum of volumes of its daughters EMS and P2; the volume of zygote P0 is the sum of the inferred volumes of its daughters AB and P1. Cell morphology (i.e., cell shape, cell surface area, and cell-cell contact relationship and area) and cell location from 1- to 8-cell stages will be used to test and verify our phase field model (S3–S6 Tables). In total, there are 5, 12, 15, 15 conserved contacts and 1, 4, 7, 10 conserved non-contacts (i.e., reproducibly found in all embryos) identified between specific cells at 4-, 6-, 7- and 8-cell stages, respectively. It should be pointed out that the stages with exact cell numbers are achieved by an invariant division sequence in embryos of *C*. *elegans* as well as its closely-related species *C*. *briggsae* (i.e., P0 → AB → P1 → ABa & ABp → EMS → P2 → ABal & ABar & ABpl & ABpr…), which has been tested in over 200 wild-type embryos [2,52].

### Construction of the phase field model

#### Mechanical interactions and constrictions

The phase-field approach, a numerical technique based on a diffuse-interface description, tracks the evolution of a phase or species concentration by a set of phase fields instead of explicit and direct tracking of the sharp interfaces. Here, we develop a phase-field approach to model the morphological and morphogenetic dynamics of a multicellular system. The boundary of the *i*-th cell is represented and tracked by a phase field *ϕ*_*i*_(***r***,*t*), *i* = 1,⋯,*N* with *ϕ*_*i*_∈[0,1], defined on a computational domain *Ω*, where *N* denotes the total amount of cells. The interior of cell *i* is *ω*_*i*_∈*Ω* where *ϕ*_*i*_ = 1, while the complementary domain *ω*_*i*_′∈*Ω* with *ϕ*_*i*_ = 0 represents the exterior of the cell. Thus, the cell membrane is defined as the narrow transition layer in between them. Apart from the cells, an additional phase field *ϕ*_e_ is employed to track the boundary of eggshell and restrict the range of cell movement, where *ϕ*_e_ = 1 represents outside of eggshell and *ϕ*_e_ = 0 represents inside.

The distribution of phase field, namely the deformation and motion of a cell, is determined by a couple of forces imposed including cell surface tension, cell-eggshell and cell-cell repulsion, cell-cell attraction, and the homogeneous pressure that constrains cell volume to be a constant. These interactions and constrictions can be expressed in both forms of free energy *E* and acting force ***F***.

Firstly, the surface energy of the *i*-th cell is defined with Ginzburg-Landau free energy [41].

$$E_{ten}=\gamma \int_{\Omega }\left(\frac{1}{2}{\left|\nabla \phi_{\dot{\iota }}\right|}^{2}+cW(\phi_{\dot{\iota }})\right)dr$$ (1)

where *γ* is the cell surface tension and *c* is a positive coefficient that determines the thickness of transition layer between interior and exterior of cells; *W*(*ϕ*) = *ϕ*^2^(*ϕ*−1)^2^ is a double-well potential with minima at *ϕ* = 0 and *ϕ* = 1 which describes the tendency of the phase field to approach 0 or 1. Minimizing the surface energy *E*_ten_ leads to the minimal surface area of a cell, in other words, the cell shape tends to be spherical. A cell with larger *γ* acquires a stronger capability to maintain its shape as a sphere. Force field of the *i*-th cell’s surface tension ***F***_ten_ is derived by taking variational derivative on surface energy *E*_ten_ in the form of line density.


$$F_{ten}=-\gamma \left(\Delta \phi_{i}-cW^{\prime }(\phi_{i})\right)\frac{\nabla \phi_{i}}{{\left|\nabla \phi_{i}\right|}^{2}}$$ (2)

Secondly, cell movements are limited in the interior of the eggshell and affected by other cells. Cell-eggshell and cell-cell repulsive potential *E*_rep_ of the *i*-th cell is taken into account and determined as below [42].

$$E_{rep}=\frac{1}{2}\int_{\Omega }\left(g_{e}\phi_{e}^{2}+g\phi_{\dot{\iota }}^{2}\sum_{j\neq \dot{\iota }}^{N}\phi_{j}^{2}\right)dr$$ (3)

where *g*_e_ and *g* are positive coefficients, denoting the strength of cell-eggshell and cell-cell repulsive energy, respectively. Minimizing the repulsive energy *E*_rep_ can reduce the overlap between phase fields and separate the cells apart. Repulsive force ***F***_rep_ is derived by taking the variational derivative for *ϕ*_*i*_ in the form of line density.


$$F_{rep}=(g_{e}\phi_{\dot{\iota }}\phi_{e}^{2}+g\phi_{\dot{\iota }}\sum_{j\neq \dot{\iota }}^{N}\phi_{j}^{2})\frac{\nabla \phi_{\dot{\iota }}}{{\left|\nabla \phi_{\dot{\iota }}\right|}^{2}}$$ (4)

Thirdly, for the possible attraction between cells such as effects of adhesive protein on membrane and gap junction between cells [10,69–71], we model it by introducing an advective item ***F***_atr_ to represent attractive interaction along the normal directions of the interface [31].

$$F_{atr}=\sum_{j\neq \dot{\iota }}^{N}\sigma_{\dot{\iota },j}\nabla \phi_{j}$$ (5)

where *σ*_*i*,*j*_ is a non-negative coefficient and positively associated with the attraction intensity between the *i*-th and *j*-th cells.

Fourthly, in an ideal situation, cell volume is constant during cell deformation and motion. Hence, a volume constraint is needed and introduced.

$$F_{vol}=M(V_{\dot{\iota }}(t)-\int_{\Omega }\phi_{\dot{\iota }}dr)\hat{n}=M(\int_{\Omega }\phi_{\dot{\iota }}dr-V_{\dot{\iota }}(t))\frac{\nabla \phi_{\dot{\iota }}}{\left|\nabla \phi_{\dot{\iota }}\right|}$$ (6)

where *M* is a positive coefficient which denotes the volume constraint strength and $\hat{n}$ is the unit normal vector at the interface which orients inward; *V*_*i*_(*t*) denotes the prescribed volume for the *i*-th cell, which remains approximately unchanged during cell cycles and updates after cell division since the cell volume is substantially reduced after mitosis.

Inside the overdamped viscous environment of an embryo, the resultant of cell surface tension (Eq 2), cell-eggshell and cell-cell repulsion (Eq 4), cell-cell attraction (Eq 5), and cell volume constriction (Eq 6) is always balanced with the viscosity ***f***, which is dominantly determined by a cell’s velocity ***u*** [9].

f=−τu (7)

$$F_{ten}+F_{rep}+F_{vol}+F_{atr}+f=0$$ (8)

where *τ* is the viscosity coefficient of the embryo’s inner environment. Finally, the evolution of all the phase fields *ϕ*_*i*_ follows $\frac{\partial \phi_{i}}{\partial t}+u∙\nabla \phi_{i}=0$, which can be consequently transformed into Eq 9.


$$\frac{\partial \phi_{i}}{\partial t}=-\frac{1}{\tau }(F_{ten}+F_{rep}+F_{vol}+F_{atr})∙\nabla \phi_{i}$$ (9)

#### Cell division

We choose a simplified mathematical description for cell division which is implemented as instantaneous bisection of phase field *ϕ*_*i*_ by a plane ***n***∙(***r***−***r***_*c*_)−*b* = 0. Location and direction of the splitting plane are determined by the cell volume segregation direction and ratio obtained from *in vivo* measurements. Division of the *i*-th cell is processed following Eqs 10 and 11 [32].

$$\phi_{N+1}^{t+1}=\phi_{i}^{t}\left(\frac{1}{2}\tanh\frac{n∙(r-r_{c})-b}{\epsilon }+\frac{1}{2}\right)$$ (10)

$$\phi_{i}^{t+1}=\phi_{i}^{t}\left(\frac{1}{2}\tanh\frac{b-n∙(r-r_{c})}{\epsilon }+\frac{1}{2}\right)$$ (11)

where $\phi_{i}^{t+1}$ and $\phi_{N+1}^{t+1}$ are the phase fields of two daughter cells at their first appearance moment; ***n*** is the unit vector along the division orientation. $r_{c}=\frac{\int_{\Omega }r\phi_{i}^{t}dr}{\int_{\Omega }\phi_{i}^{t}dr}$ is the mass center of mother cell; *b* is a constant obtained by minimizing $L(b)=\left|\frac{\int_{\Omega }\phi_{i}^{t+1}dr}{\int_{\Omega }\phi_{N+1}^{t+1}dr}-\frac{V_{i}(t)}{V_{N+1}(t)}\right|$, which controls the volume segregation ratio between two daughter cells; *ϵ* controls the width of the interface between two daughter cells. It’s worth noting that both the cell division order and cell division orientation are kept in line with the experimental observations when simulating the real scenes (S2–S6 Tables), and then we disturb them to discover their roles in embryonic morphogenesis.

#### Parameter setting

We use a 256×256×128 cuboid grid as a computational domain with grid size d*l* = 0.2508 μm and set the time step as *h* = 0.1 throughout all simulations. For comparison, the major and minor semi-axes of observed eggshells are 27.5837 μm (*x*) and 18.3477 μm (*z*) respectively. As the eggshell in imaging experiments was compressed about 16.7942 μm narrower in total along the left-right axis (*y*) by slide mounting, we set a cutoff on the left-right axis (*y*) based on the experimental values (half width = 9.9506 μm) and then construct a compressed eggshell as boundary (Fig 1C) [34].

To build up a minimal model that has the least physical constraints but outlines the most significant characteristics of a developing embryo, we complicate the system step by step. We first neglect the intercellular attraction (i.e., *σ*_*i*,*j*_ = 0), then only three independent physical coefficients are left: cell surface tension (*γ*), transition layer thickness (*c*), and cell-eggshell repulsive energy (*g*_e_). For the other system parameters, we assign constant values as *g* = 1.6 (mechanical unit), *M* = 0.0012, *τ* = 2.62 and *ϵ* = 2^−52^ throughout all simulations. Sensitivity and comparison analysis is carried out on the physical coefficients *γ*, *c* and *g*_e_ to determine their optimal values, by evaluating methods as the following.

Maximizing the deformation of a cell compared with a standard sphere (*α*), according to Eqs 12 and 13.

$$\bar{r}={\left(\frac{3}{4\pi }\int_{\Omega }\phi dr^{3}\right)}^{\frac{1}{3}}$$ (12)

$$\alpha =\int_{\partial \omega }\frac{{\left(R-\bar{r}\right)}^{2}}{{\bar{r}}^{2}R^{2}}ds$$ (13)

where $\bar{r}$ is the average radius of a cell obtained from its equivalent sphere with the same volume; ∂*ω* is the cell surface determined by *ϕ* = 0.5; *R* is the distance between panel element d*s* and the cell’s mass center $r_{c}=\frac{\int_{\Omega }r\phi_{i}^{t}dr}{\int_{\Omega }\phi_{i}^{t}dr}$.

1. Maximizing the phase-field gradient at interfaces (|∇*ϕ*|_max_). This requirement sharpens the cell boundary and draws a clear distinction between cells.
2. Minimizing the average positional variation between simulation and experiment, according to Eq 14.

$$\eta =\sqrt{\frac{1}{N}\sum_{i=1}^{N}{\left(r_{sim,i}-r_{exp,i}\right)}^{2}}$$ (14)

where ***r***_sim,*i*_ and ***r***_exp,*i*_ represent the 3D position of cell *i* in simulation and experiment respectively; *η* is the average positional variation.

These optimization operations result in a parameter combination of *γ* = 0.25, *c* = 1.0 and *g*_e_ = 16, which are used in all simulations in this work (S19A and S19B and S20 Figs). Note that the embryo structure is insensitive to the exact value of *g*_e_, thus we assume that the stiffness of the eggshell is one magnitude larger than that of a cell, namely *g*_e_ = 10*g* = 16 (S20 Fig) [22]. Importantly, all the attraction coefficients between cells (*σ*_*i*,*j*_) are set as zero at the start and assigned with different values in later simulations for in-depth investigation, as a high-dimensional factor that may diversify the developmental paths.

### Fluorescence microscopy for *C*. *elegans* early embryogenesis

#### Shooting with high temporal resolution

The experimental operations of fast shooting are as described before [51], except for several parameter modifications. The 2-cell embryo (strain ZZY0535 [40]), which ubiquitously expresses GFP marker labeling cell nucleus and mCherry marker labeling cell membrane, was dissected from the adult worm. It was mounted for imaging using 1% methylcellulose in Boyd buffer with 20 μm microspheres. Imaging was performed with a confocal microscope equipped with two hybrid detectors at a constant room temperature of 21°C. Images were consecutively collected for both GFP and mCherry channels using a water immersion objective. By using a resonance scanner, both channels were imaged with a scanning speed of 8000 Hz and a frame size of 712 × 512 pixels (0.09 μm/pixel) per channel, while the *Z*-resolution (along the shooting direction) is set to be 0.59 μm/layer. The excitation laser beams used for GFP and mCherry are 488 nm and 594 nm, respectively. Images were continuously collected for 260 time points at a 10-sec interval, covering the 2- to 15-cell stages. *Z*-axis compensation was 0.4–4% for the 488 nm laser and 19–95% for the 594 nm laser. The pinhole size was 2.3 AU (airy unit).

#### Labeling on adhesive protein HMR-1

Micrographs of HMR-1::GFP expressing embryo (strain LP172 [48]) were acquired with a confocal microscope with an objective of 63× magnification. The 1- to 2-cell embryo was dissected from a young adult worm and mounted with Boyd buffer/methylcellulose containing microspheres (20 μm) [35]. For 3D time-lapse imaging, GFP illuminated with a 488 nm laser beam as well as the micrographs of its expression were collected with a hybrid detector through a water immersion objective. The imaging setting was like what was used previously, namely, a frame size of 712×512 pixels (0.09 μm/pixel) with 8000 hertz (Hz) scanning speed using a resonance scanner [51]. Laser compensation was applied during the stack acquisition to ensure the comparable brightness of the images acquired between the lower stack and upper stack. Micrographs from 68 focal planes were collected consecutively from top to bottom of the embryo at an interval of about 1.41 minutes and with a *Z*-axis resolution of 0.71 μm. Images were continuously collected for 53 time points (from 2- to ~15-cell stages) using a 2.5 AU (airy unit) pinhole size. *Z*-axis compensation was 0.5–8% for 488 nm laser. Finally, the 3D projection was deconvoluted and generated.

#### Laser ablation on P2 cell

The experimental operations of cell ablation are the same as the one described previously [40], except that the bleaching time is readjusted for the new target cell P2. The 4-cell embryos with lineaging marker labeling all the cell nuclei were selected for 4D imaging [37]. The imaging lasts for 40 time points from 2- to ~23-cell stages, and has a spatial resolution of 0.09 μm/pixel in the focal plane and 1.41 μm/pixel perpendicular to the focal plane. Immediately after the target cell P2 and its sister EMS completed mitosis (i.e., complete division of their mother P1), imaging was terminated and the following procedures were performed within 1.5 min: (1) switching of the imaging mode from live data mode to normal mode; (2) focusing on the middle plane of the target cell nucleus; (3) selecting the bleaching point from the panel and creating a region of interest in the middle of the target cell nucleus in a preview panel; (4) turning off all the fluorescence detectors except the one for the DIC and switching the filter to “substrate”; (5) setting the bleaching time (90 seconds); (6) temporally closing the shutters for all the wavelengths except the pulsed diode laser, which emits a 405-nm laser beam; tuning it to 100% intensity and starting the bleaching; (7) once completed, switching back to the live data mode and resuming the 4D imaging as usual.

## Supporting information

S1 Fig Comparison of embryo morphology between simulation without cell-cell attraction and experiment from 1- to 4-cell stages (view from *y* / left-right axis). The 1^st^ and 2^nd^ columns, cell-arrangement progression in phase-field simulation; the time point of each embryonic structure is illustrated on its top; dashed arrows, cell division orientation measured by experiment and inputted into simulation; the 3^rd^ column, a live embryo with mCherry fluorescence on cell membrane (strain ZZY0535 [40]); scale bar, 10 μm.
(TIF)

S2 Fig Relationship between cell surface tension (*γ*) and the curvature of interface between cells. In the 5 simulations from left to right, the ratio between *γ*_AB_ and *γ*_P1_ is changed from 1.00:0.25 to 0.25:1.00; blue cell, AB; orange cell, P1.
(TIF)

S3 Fig Relationship between symmetric global attraction (*σ*) and the hollows at junction points among cells. In the 6 simulations from top left corner to bottom right corner, *σ* is changed from 0.0 to 1.5, equally applied onto all the 5 cell-cell contacts; blue cell, ABa; cyan cell, ABp; yellow cell, EMS; red cell, P2.
(TIF)

S4 Fig Distribution of membrane-attached E-cadherin HMR-1 from 2- to 6-cell stages. The interfaces with distinguishably high and low accumulation are indicated by blue and red arrows respectively. The imaging time point of each subfigure (1 ~ 12) is denoted in its top left corner; subfigures 1 ~ 4, formation of 2- to 4-cell structures; subfigures 10 ~ 11, formation of 6-cell structure; some interfaces near the focal planes are not identified or illustrated due to their blurry fluorescence, such as the interfaces of ABpl-ABpr, ABpl-P2, and ABpr-P2 contacts.
(TIF)

S5 Fig Comparison of embryo morphology between simulation and experiment at the last moment of 8-cell stage, without attraction motif on ABpl-E contact, i.e., *σ*_ABpl, E_ = *σ*_S_. Embryo morphology in simulation and experiment are respectively illustrated on the left and right in three orthogonal observation directions, while a cell-cell contact map is placed in their top left corners. About the map in simulation, dark and light gray shades denote relatively strong (*σ* = *σ*_S_) and weak (*σ* = *σ*_W_) attraction respectively, while black dots represent the contacted cell pairs. About the map in experiment, black dots represent the conserved contacted cell pairs, while empty circles represent the unconserved contacted cell pairs [34]. The relationship between cell identity and color is listed next to the contact maps. The quantitative experimental data is obtained at the last moment (time point) of each stage.
(TIF)

S6 Fig Morphological evolution during 6-cell stage, with simulation time long enough for the whole system to reach mechanical equilibrium. The curves of average velocity (upper) and its change rate (lower) are illustrated side by side. The solid and dashed vertical black lines denote the extreme points in the two curves respectively, while the 3D structures at those time points are illustrated on top and bottom, pointed by gray arrows originating from their corresponding lines. The last structure in the bottom right is the system’s terminal state approaching mechanical equilibrium. The change of cell-cell contact map is illustrated by different colors in the background, while the detail is written between two consecutive structures. The time point of the first quasi-steady state is indicated by a black triangle. The relationship between cell identity and color is listed in the bottom left corner.
(TIF)

S7 Fig Morphological evolution during 8-cell stage, with simulation time long enough for the whole system to reach mechanical equilibrium. (A). The upper panel, without attraction motif on ABpl-E contact, i.e., *σ*_ABpl, E_ = *σ*_S_. (B). The lower panel, with attraction motif on ABpl-E contact, i.e., *σ*_ABpl, E_ = *σ*_W_. For each panel, the curves of average velocity (upper) and its change rate (lower) are illustrated side by side. The solid and dashed vertical black lines denote the extreme points in the two curves respectively, while the 3D structures at those time points are illustrated on top and bottom, pointed by gray arrows originating from their corresponding lines. The last structure in the bottom right is the system’s terminal state approaching mechanical equilibrium. The change of cell-cell contact map is illustrated by different colors in the background, while the detail is written between two consecutive structures. The time point of the second quasi-steady state is indicated by a black triangle. The relationship between cell identity and color is listed in the bottom left corner.
(TIF)

S8 Fig Average velocity of all cells in a live embryo from 6- to 8-cell stages. The *C*. *elegans* wild-type embryo is imaged at an interval of 10 seconds, and each cell’s nucleus is traced and used for the calculation of its motion velocity. The average velocity (defined by the root-mean-square velocity of all cells) reveals severe perturbation during cell division (highlighted with red line) and subsequently a full relaxation at all stages (noted with negative correlation coefficient *r* < -0.5), i.e., the cell arrangement approaches to the quasi-steady state with slow motion (average velocity < 0.05 μm/s). The time range of cell division is labeled with a light red column and the exceptional accelerated motion (noted with positive correlation coefficient *r* = 0.75) that takes place in the second half of 8-cell stage is separated with a dashed gray line and highlighted with a dashed gray arrow.
(TIF)

S9 Fig Eight different developmental paths under perturbation on cell division timing. The structures at quasi-steady states are labeled by ⓪, ①, ②, ③, ④, ⑤, ⑥, ⑦ from the top left corner to the bottom right corner, corresponding to Fig 4C. In each panel, embryo morphology in simulation is illustrated in three orthogonal observation directions, while a cell-cell contact map is placed in the top left corner. About the map in simulation, dark gray and light gray shades denote relatively strong attraction (*σ* = *σ*_S_) and weak attraction (*σ* = *σ*_W_) respectively, while black dots represent the contacted cell pairs. The relationship between cell identity and color is listed next to the contact maps.
(TIF)

S10 Fig Cell-arrangement progression of two *pad-1* RNAi-treated embryos from 7- to 8-cell stages, revealing reproducible defective P2 division orientation and subsequent abnormal cell positions. Each color represents one specific cell identity, denoted in the legend; the misarranged cells in RNAi-treated embryos are illustrated with black points, while the normal cells are illustrated with their original colors according to the legend. For each cell, a region formed by nuclei positions from 222×0.95 ≈ 210 independent wild-type embryos is illustrated for visual comparison. Data of both wild-type and RNAi-treated embryos are obtained from a previously established dataset [2].
(TIF)

S11 Fig Simulation for 8-cell stage with single attraction motif added on the cell-cell attraction matrix default. (A). 3D structures at time point 350000. The ones with ABpl-MS, ABpl-E, and C-E motifs are three-dimensional and highlighted with red rectangles. (B). 3D structures at time point 450000. The ones with ABpl-E and C-E motifs are three-dimensional and highlighted with red rectangles. The relationship between cell identity and color is listed in the bottom right corner.
(TIF)

S12 Fig Independent cell-cell contact map identified in the 17 simulations with single attraction motif added on the cell-cell attraction matrix default. The Topologies 0 ~ 26 corresponding to Fig 6A are listed from the top left corner to the bottom right corner. For each map, black dots represent the contacted cell pairs.
(TIF)

S13 Fig Curves of average velocity and its change rate in simulations with *σ*_ABpl, E_ = *σ*_W_ = 0.2, *σ*_ABpl, MS_ = *σ*_S_ = 0.9 (upper) and *σ*_ABpl, E_ = *σ*_W_ = 0.2, *σ*_ABpl, MS_ = *σ*’_S_ = 1.6 (lower) at 8-cell stage. A total of 16 pairs of critical time points in the curves of average velocity and its change rate are aligned and connected between the two panels (embryos), using solid and dashed red lines respectively.
(TIF)

S14 Fig Comparison between simulations on uncompressed and compressed embryos. (A). Embryo morphology of uncompressed (1^st^ row) and compressed (2^nd^ row) embryos from 1- to 7-cell stages, without any attraction motif added. (B). Embryo morphology in uncompressed (1^st^ and 2^nd^ columns) and compressed (3^rd^ and 4^th^ columns) embryos at 8-cell stage, with or without the ABpl-E motif, revealing that the ABpl-E motif is essential for the compressed embryo to reach the correct structure as seen *in vivo*, but not for the uncompressed one.
(TIF)

S15 Fig Morphological evolution of an uncompressed embryo during 6-cell stage, with simulation time long enough for the whole system to reach mechanical equilibrium. The curves of average velocity (upper) and its change rate (lower) are illustrated side by side. The solid and dashed vertical black lines denote the extreme points in the two curves respectively, while the 3D structures at those time points are illustrated on bottom, pointed by gray arrows originating from their corresponding lines. The last structure in the bottom right is the system’s terminal state approaching mechanical equilibrium. The relationship between cell identity and color is listed in the bottom left corner.
(TIF)

S16 Fig Morphological evolution of an uncompressed embryo during 7-cell stage, with simulation time long enough for the whole system to reach mechanical equilibrium. The curves of average velocity (upper) and its change rate (lower) are illustrated side by side. The solid and dashed vertical black lines denote the extreme points in the two curves respectively, while the 3D structures at those time points are illustrated on bottom, pointed by gray arrows originating from their corresponding lines. The last structure in the bottom right is the system’s terminal state approaching mechanical equilibrium. The change of cell-cell contact map is illustrated by different colors in the background, while the detail is written between two consecutive structures. The relationship between cell identity and color is listed in the bottom left corner.
(TIF)

S17 Fig Morphological evolution of an uncompressed embryo during 8-cell stage, with simulation time long enough for the whole system to reach mechanical equilibrium. (A). The upper panel, without attraction motif on ABpl-E contact, i.e., *σ*_ABpl, E_ = *σ*_S_. (B). The lower panel, with attraction motif on ABpl-E contact, i.e., *σ*_ABpl, E_ = *σ*_W_. For each panel, the curves of average velocity (upper) and its change rate (lower) are illustrated side by side. The solid and dashed vertical black lines denote the extreme points in the two curves respectively, while the 3D structures at those time points are illustrated on top and bottom, pointed by gray arrows originating from their corresponding lines. The last structure in the bottom right is the system’s terminal state approaching mechanical equilibrium. The change of cell-cell contact map is illustrated by different colors in the background, while the detail is written between two consecutive structures. The relationship between cell identity and color is listed in the bottom left corner.
(TIF)

S18 Fig Embryo width (estimated with the maximum nucleus distance in *y* direction) of wild-type and P2-ablated embryos. The significance level is obtained by one-tailed Wilcoxon rank-sum test. (A). Distribution at the last time point of 6-cell stage. (B). Distribution at the last time point of 7-cell stage. (C). Distribution at the last time point of 8-cell stage.
(TIF)

S19 Fig Sensitivity and comparison analysis on composite parameters *γ* (0.05, 0.25, 0.50, 1.00, 2.00) and *γc* (0.25, 0.50, 1.00, 2.00, 4.00) with *g*_e_ = 16.0. The optimum fitting result (*γ* = 0.25, *c* = 1.0) is highlighted by red rectangles. (A). Phase-field distribution at 4-cell stage, with interface transition quantified by maximum gradient |∇*ϕ*|_max_. (B). Embryo morphology at 4-cell stage, with cell deformation quantified by coefficient *α* (Eqs 12 and 13).
(TIF)

S20 Fig Sensitivity and comparison analysis on parameter *g*_e_ (1.6, 5.2, 8.8, 12.4, 16.0) with *γ* = 0.25 and *c* = 1.0. The average positional variation *η* is used to evaluate the deviation between structures in simulation and experiment (Eq 14).
(TIF)

S1 Table Information of the embryos collected from datasets produced previously. (DOCX)

S2 Table Volume and division orientation of the cells up to 8-cell stage. (DOCX)

S3 Table Cell surface area, cell-cell contact relationship and area at 4-cell stage. (DOCX)

S4 Table Cell surface area, cell-cell contact relationship and area at 6-cell stage. (DOCX)

S5 Table Cell surface area, cell-cell contact relationship and area at 7-cell stage. (DOCX)

S6 Table Cell surface area, cell-cell contact relationship and area at 8-cell stage. (DOCX)

S7 Table Comparison between simulation (*σ* = 0.0, 0.3, 0.6, 0.9, 1.2, 1.5 for all contacts) and experiment at 4-cell stage. (DOCX)

S8 Table Comparison between simulation (*σ* = 0.9 for ABa-ABp, ABa-EMS, ABp-EMS, ABp-P2 contacts, *σ*_EMS, P2_ = 0.0, 0.2, 0.4, 0.6, 0.8) and experiment at 4-cell stage. (DOCX)

S9 Table Preservation time of cell-cell contact map in simulation with a single attraction motif added at 8-cell stage. (DOCX)

S10 Table Corresponding timing between simulations with *σ*_ABpl, E_ = 0.2, *σ*_ABpl, MS_ = 0.9 and *σ*_ABpl, E_ = 0.2, *σ*_ABpl, MS_ = 1.6 at 8-cell stage. (DOCX)

S1 Movie Phase-field simulation on the structural evolution of a compressed embryo from 1- to 4-cell stages, without cell-cell attraction. (MP4)

S2 Movie Phase-field simulation on the structural evolution of a compressed embryo from 1- to 4-cell stages, with cell-cell attraction. (MP4)

S3 Movie Phase-field simulation on the structural evolution of a compressed embryo at 6-cell stage. (MP4)

S4 Movie Phase-field simulation on the structural evolution of a compressed embryo at 7-cell stage. (MP4)

S5 Movie Phase-field simulation on the structural evolution of a compressed embryo at 8-cell stage, without attraction motif on ABpl-E contact. (MP4)

S6 Movie Phase-field simulation on the structural evolution of a compressed embryo at 8-cell stage, with attraction motif on ABpl-E contact. (MP4)

S7 Movie Phase-field simulation on the structural evolution of an uncompressed embryo at 6-cell stage. (MP4)

S8 Movie Phase-field simulation on the structural evolution of an uncompressed embryo at 7-cell stage. (MP4)

S9 Movie Phase-field simulation on the structural evolution of an uncompressed embryo at 8-cell stage, without attraction motif on ABpl-E contact. (MP4)

S10 Movie Phase-field simulation on the structural evolution of an uncompressed embryo at 8-cell stage, with attraction motif on ABpl-E contact. (MP4)

S11 Movie Phase-field simulation on the structural evolution of a compressed embryo from 1 to 8-cell stages, with attraction motif on ABpl-E contact at 8-cell stage. (MP4)
