# Supplementary figures and images for: Computable early Caenorhabditis elegans embryo with a phase field model
Source: PLoS Comput Biol. 2022 Jan 14;18(1):e1009755. doi: 10.1371/journal.pcbi.1009755 (PMC8794267; doi:10.1371/journal.pcbi.1009755)

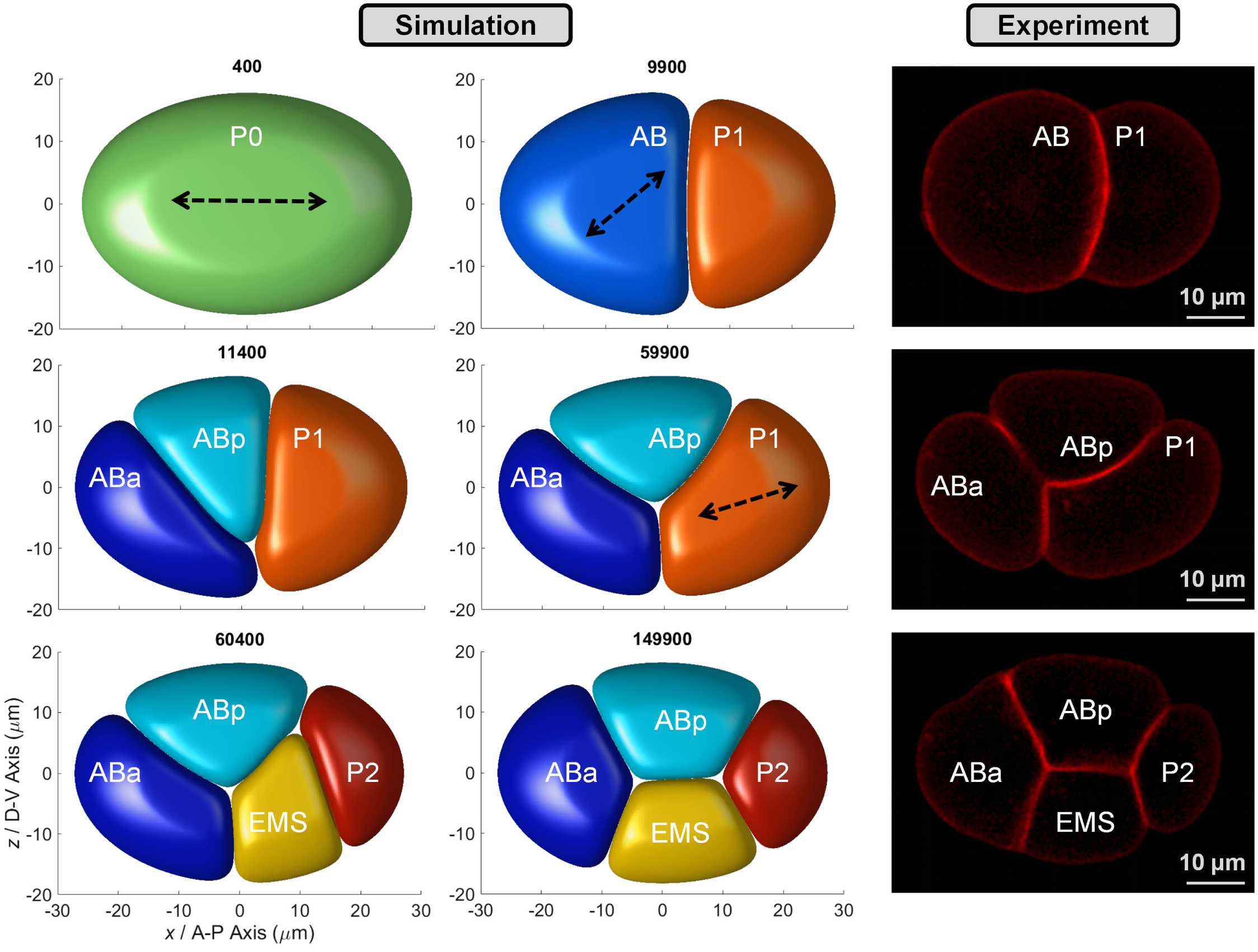

Supplement: S1 Fig — The 1st and 2nd columns, cell-arrangement progression in phase-field simulation; the time point of each embryonic structure is illustrated on its top; dashed arrows, cell division orientation measured by experiment and inputted into simulation; the 3rd column, a live embryo with mCherry fluorescence on cell membrane (strain ZZY0535 [40]); scale bar, 10 μm. (TIF) [file pcbi.1009755.s001.tif]

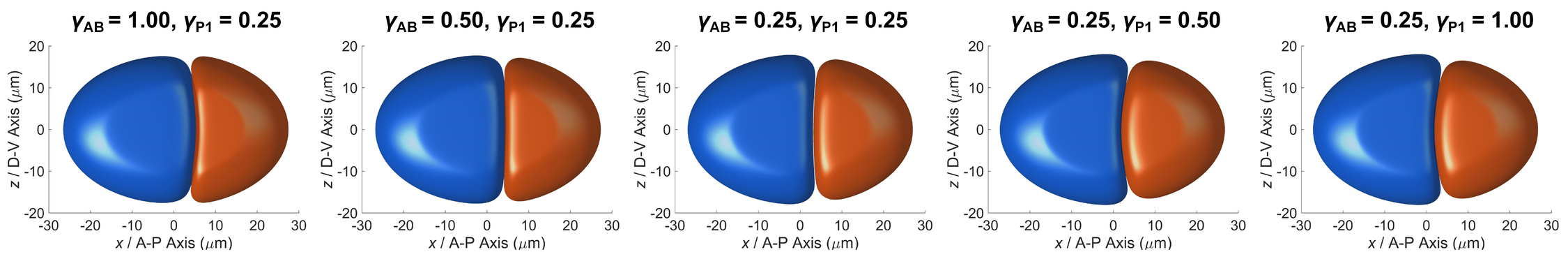

Supplement: S2 Fig — In the 5 simulations from left to right, the ratio between γAB and γP1 is changed from 1.00:0.25 to 0.25:1.00; blue cell, AB; orange cell, P1. (TIF) [file pcbi.1009755.s002.tif]

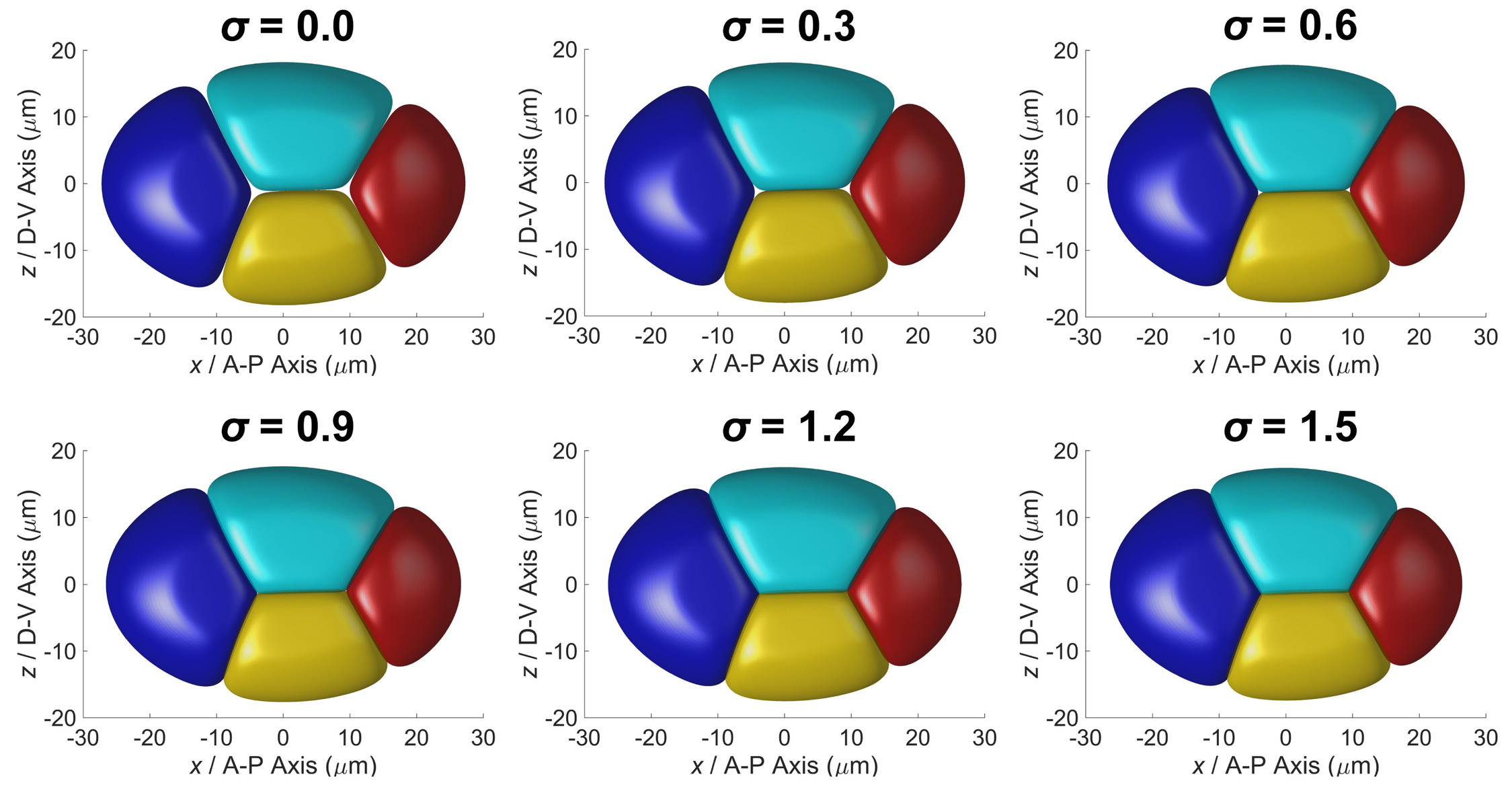

Supplement: S3 Fig — In the 6 simulations from top left corner to bottom right corner, σ is changed from 0.0 to 1.5, equally applied onto all the 5 cell-cell contacts; blue cell, ABa; cyan cell, ABp; yellow cell, EMS; red cell, P2. (TIF) [file pcbi.1009755.s003.tif]

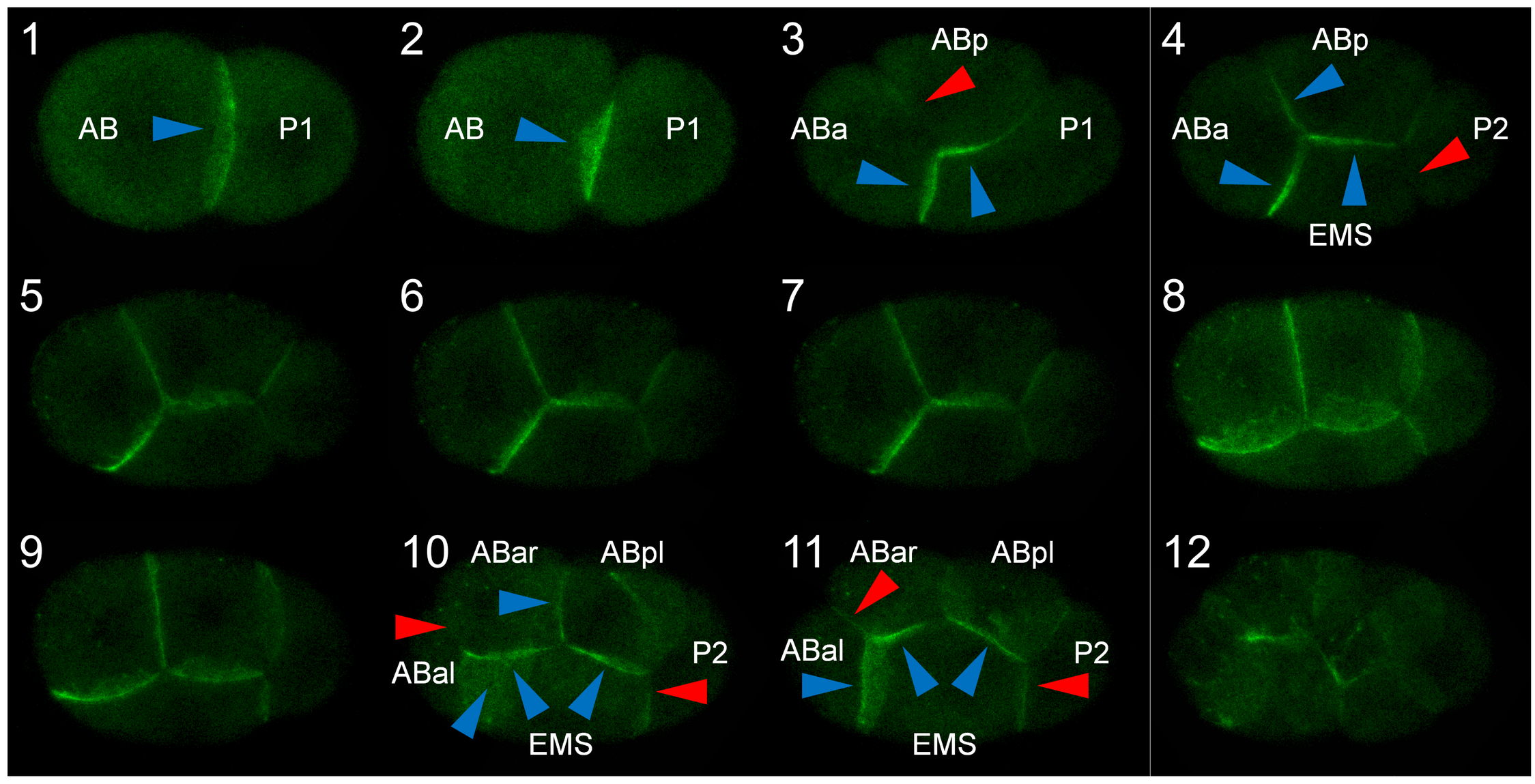

Supplement: S4 Fig — The interfaces with distinguishably high and low accumulation are indicated by blue and red arrows respectively. The imaging time point of each subfigure (1 ~ 12) is denoted in its top left corner; subfigures 1 ~ 4, formation of 2- to 4-cell structures; subfigures 10 ~ 11, formation of 6-cell structure; some interfaces near the focal planes are not identified or illustrated due to their blurry fluorescence, such as the interfaces of ABpl-ABpr, ABpl-P2, and ABpr-P2 contacts. (TIF) [file pcbi.1009755.s004.tif]

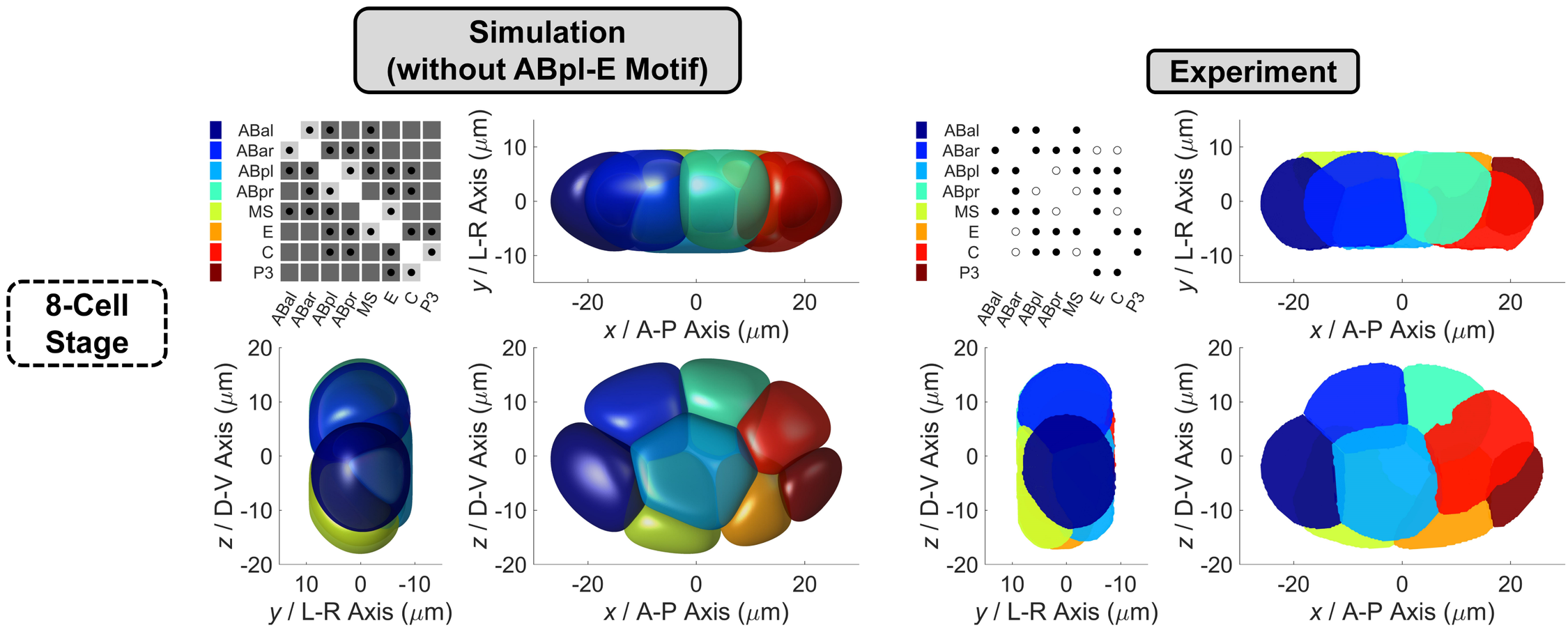

Supplement: S5 Fig — Embryo morphology in simulation and experiment are respectively illustrated on the left and right in three orthogonal observation directions, while a cell-cell contact map is placed in their top left corners. About the map in simulation, dark and light gray shades denote relatively strong (σ = σS) and weak (σ = σW) attraction respectively, while black dots represent the contacted cell pairs. About the map in experiment, black dots represent the conserved contacted cell pairs, while empty circles represent the unconserved contacted cell pairs [34]. The relationship between cell identity and color is listed next to the contact maps. The quantitative experimental data is obtained at the last moment (time point) of each stage. (TIF) [file pcbi.1009755.s005.tif]

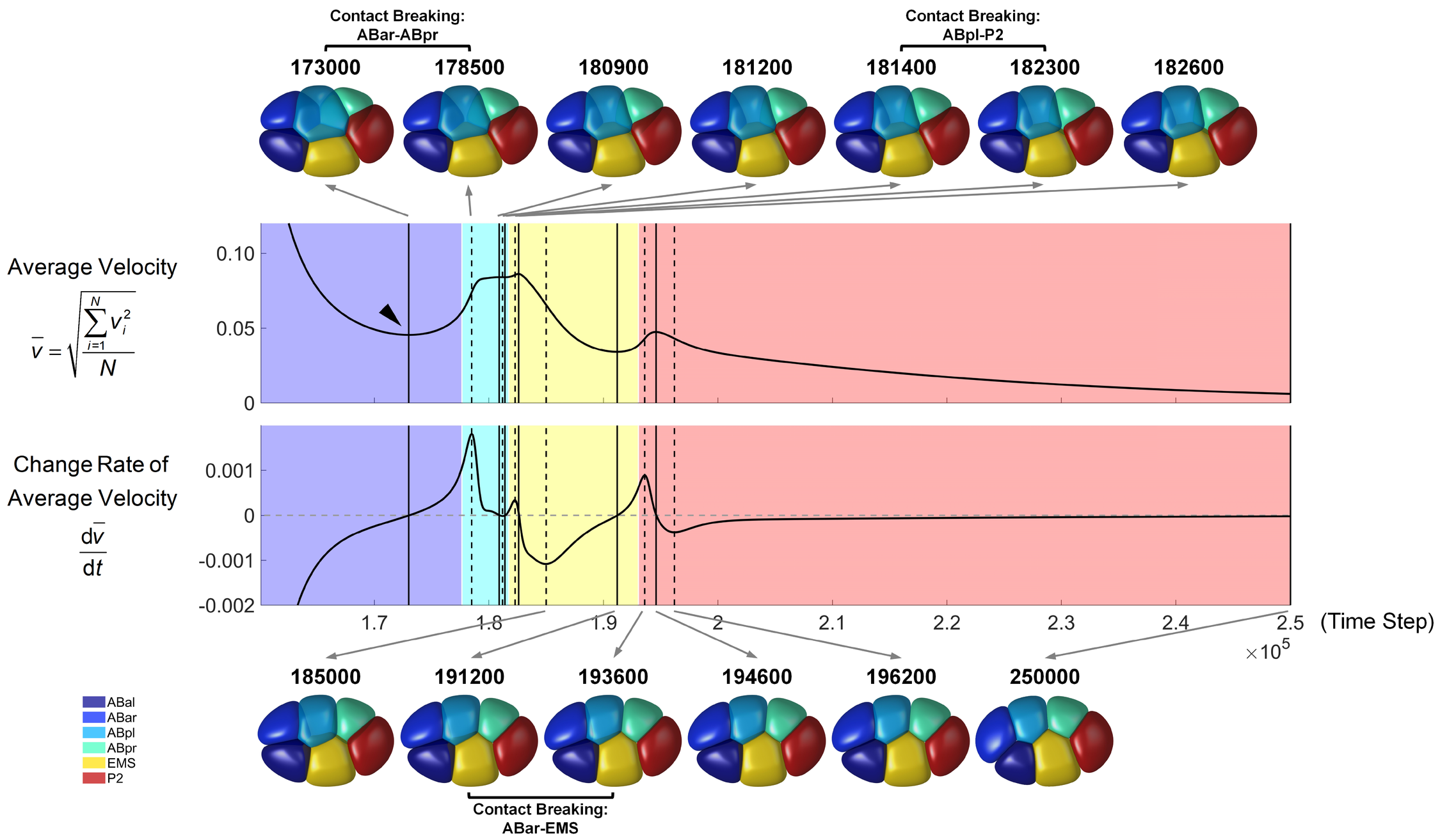

Supplement: S6 Fig — The curves of average velocity (upper) and its change rate (lower) are illustrated side by side. The solid and dashed vertical black lines denote the extreme points in the two curves respectively, while the 3D structures at those time points are illustrated on top and bottom, pointed by gray arrows originating from their corresponding lines. The last structure in the bottom right is the system’s terminal state approaching mechanical equilibrium. The change of cell-cell contact map is illustrated by different colors in the background, while the detail is written between two consecutive structures. The time point of the first quasi-steady state is indicated by a black triangle. The relationship between cell identity and color is listed in the bottom left corner. (TIF) [file pcbi.1009755.s006.tif]

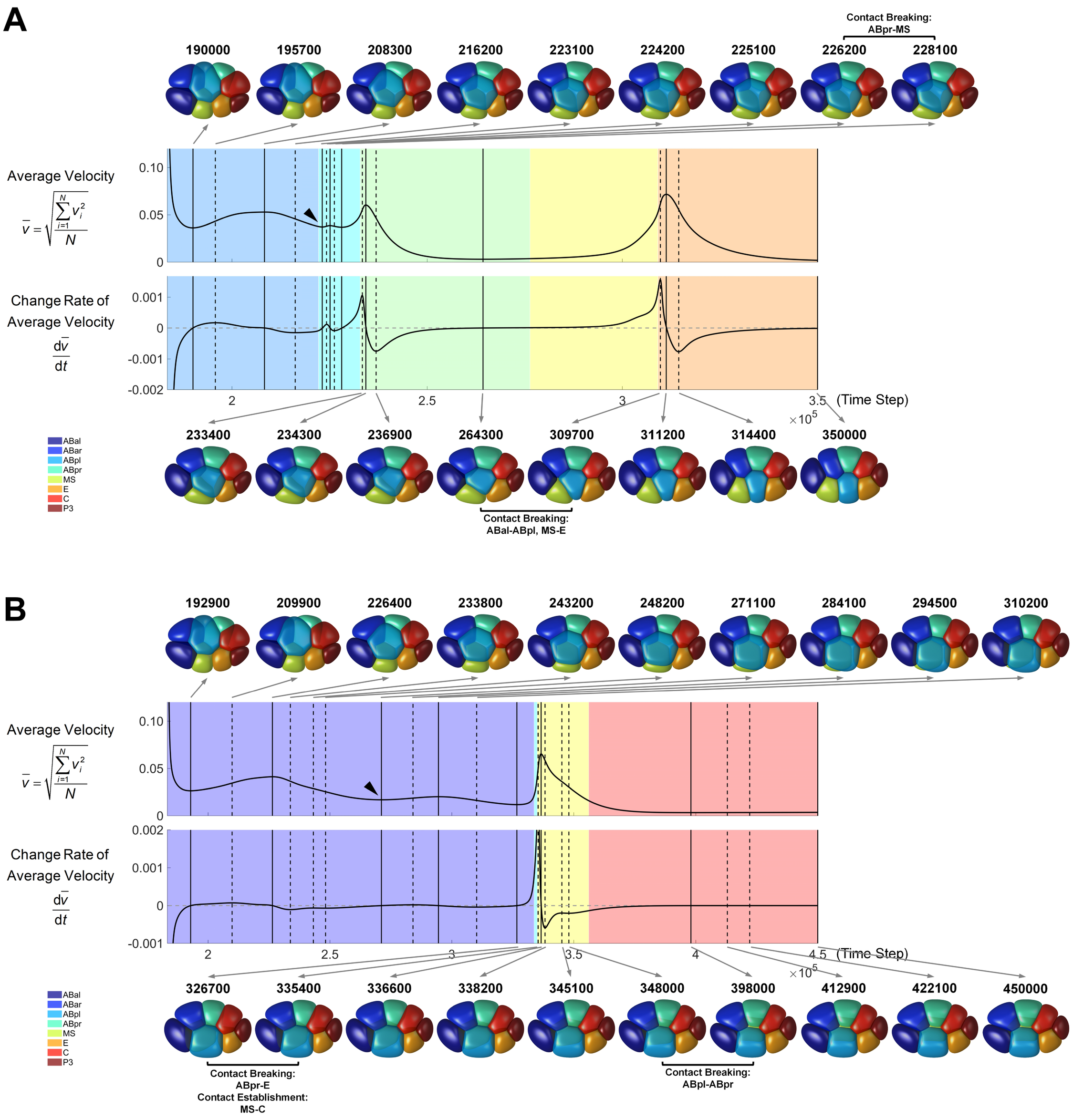

Supplement: S7 Fig — (A). The upper panel, without attraction motif on ABpl-E contact, i.e., σABpl, E = σS. (B). The lower panel, with attraction motif on ABpl-E contact, i.e., σABpl, E = σW. For each panel, the curves of average velocity (upper) and its change rate (lower) are illustrated side by side. The solid and dashed vertical black lines denote the extreme points in the two curves respectively, while the 3D structures at those time points are illustrated on top and bottom, pointed by gray arrows originating from their corresponding lines. The last structure in the bottom right is the system’s terminal state approaching mechanical equilibrium. The change of cell-cell contact map is illustrated by different colors in the background, while the detail is written between two consecutive structures. The time point of the second quasi-steady state is indicated by a black triangle. The relationship between cell identity and color is listed in the bottom left corner. (TIF) [file pcbi.1009755.s007.tif]

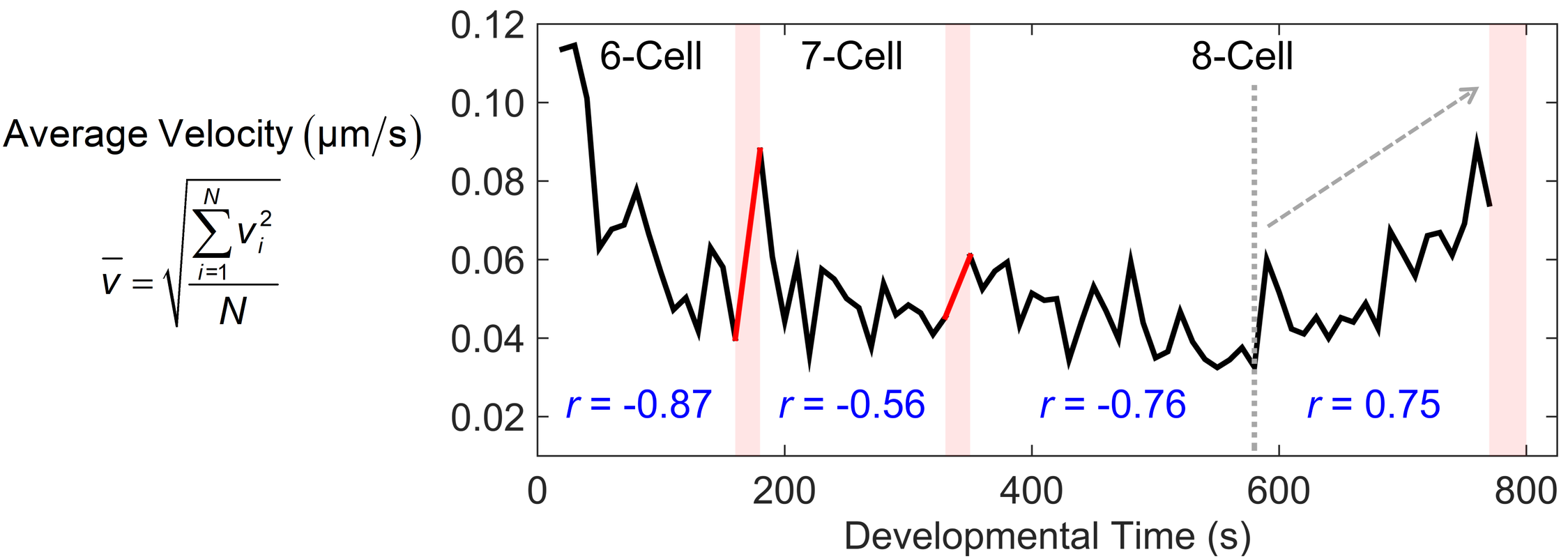

Supplement: S8 Fig — The C. elegans wild-type embryo is imaged at an interval of 10 seconds, and each cell’s nucleus is traced and used for the calculation of its motion velocity. The average velocity (defined by the root-mean-square velocity of all cells) reveals severe perturbation during cell division (highlighted with red line) and subsequently a full relaxation at all stages (noted with negative correlation coefficient r < -0.5), i.e., the cell arrangement approaches to the quasi-steady state with slow motion (average velocity < 0.05 μm/s). The time range of cell division is labeled with a light red column and the exceptional accelerated motion (noted with positive correlation coefficient r = 0.75) that takes place in the second half of 8-cell stage is separated with a dashed gray line and highlighted with a dashed gray arrow. (TIF) [file pcbi.1009755.s008.tif]

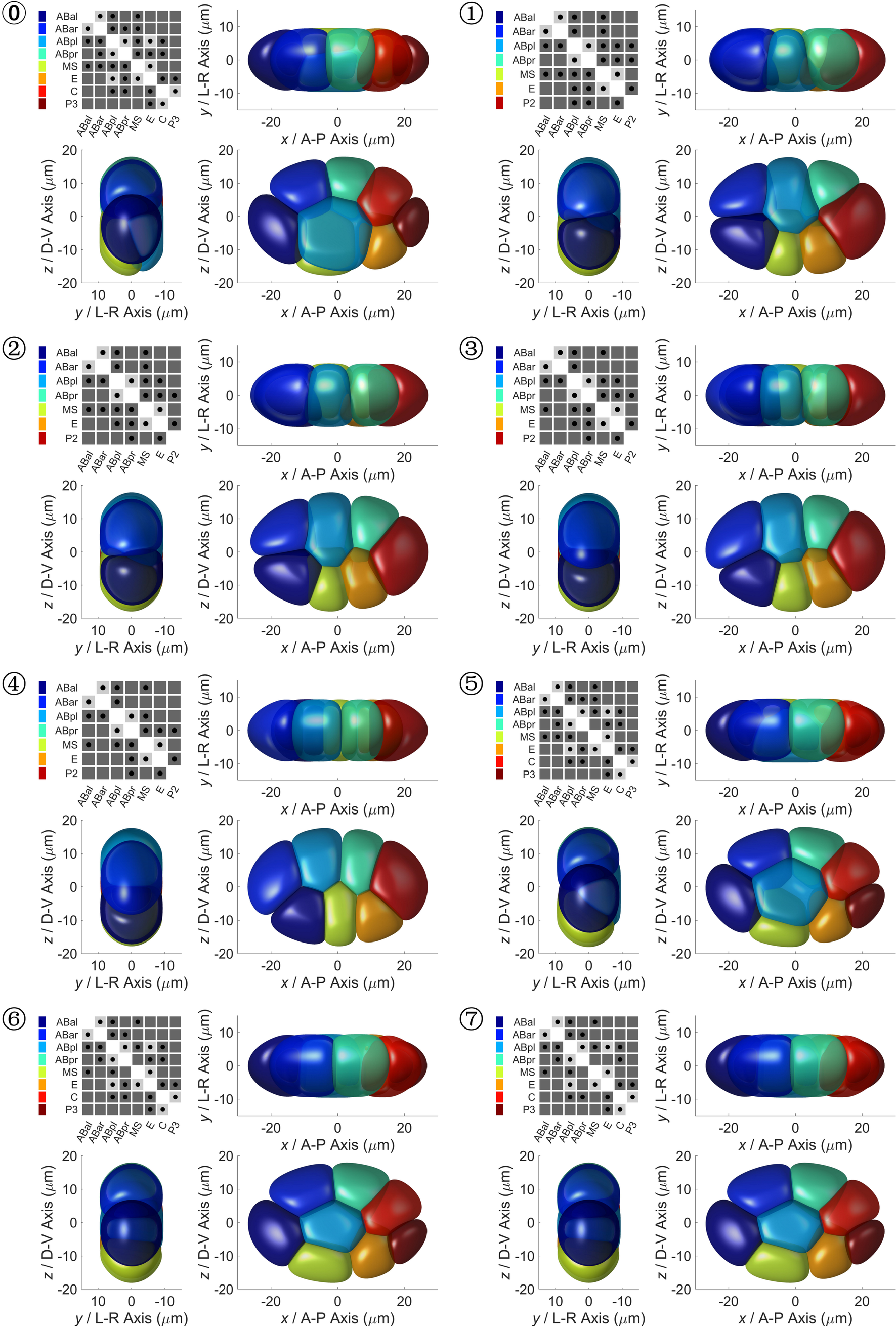

Supplement: S9 Fig — The structures at quasi-steady states are labeled by ⓪, ①, ②, ③, ④, ⑤, ⑥, ⑦ from the top left corner to the bottom right corner, corresponding to Fig 4C. In each panel, embryo morphology in simulation is illustrated in three orthogonal observation directions, while a cell-cell contact map is placed in the top left corner. About the map in simulation, dark gray and light gray shades denote relatively strong attraction (σ = σS) and weak attraction (σ = σW) respectively, while black dots represent the contacted cell pairs. The relationship between cell identity and color is listed next to the contact maps. (TIF) [file pcbi.1009755.s009.tif]

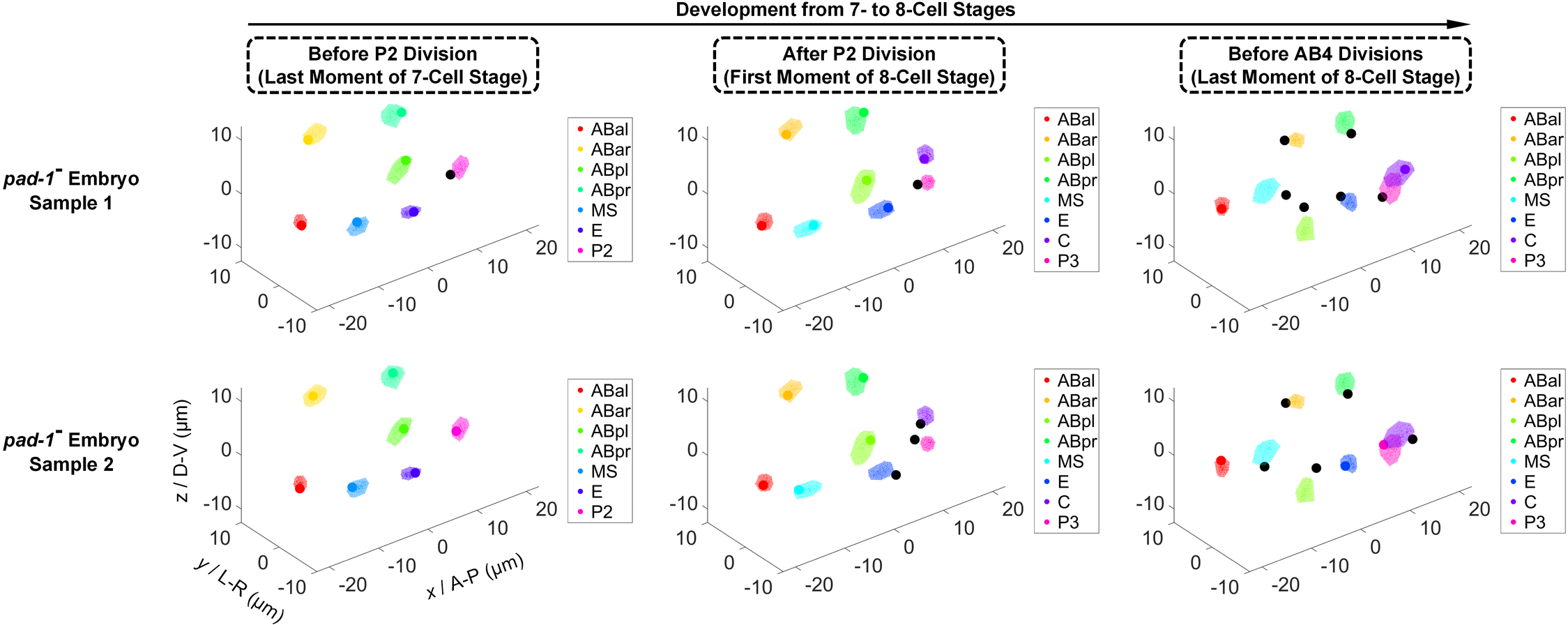

Supplement: S10 Fig — Each color represents one specific cell identity, denoted in the legend; the misarranged cells in RNAi-treated embryos are illustrated with black points, while the normal cells are illustrated with their original colors according to the legend. For each cell, a region formed by nuclei positions from 222×0.95 ≈ 210 independent wild-type embryos is illustrated for visual comparison. Data of both wild-type and RNAi-treated embryos are obtained from a previously established dataset [2]. (TIF) [file pcbi.1009755.s010.tif]

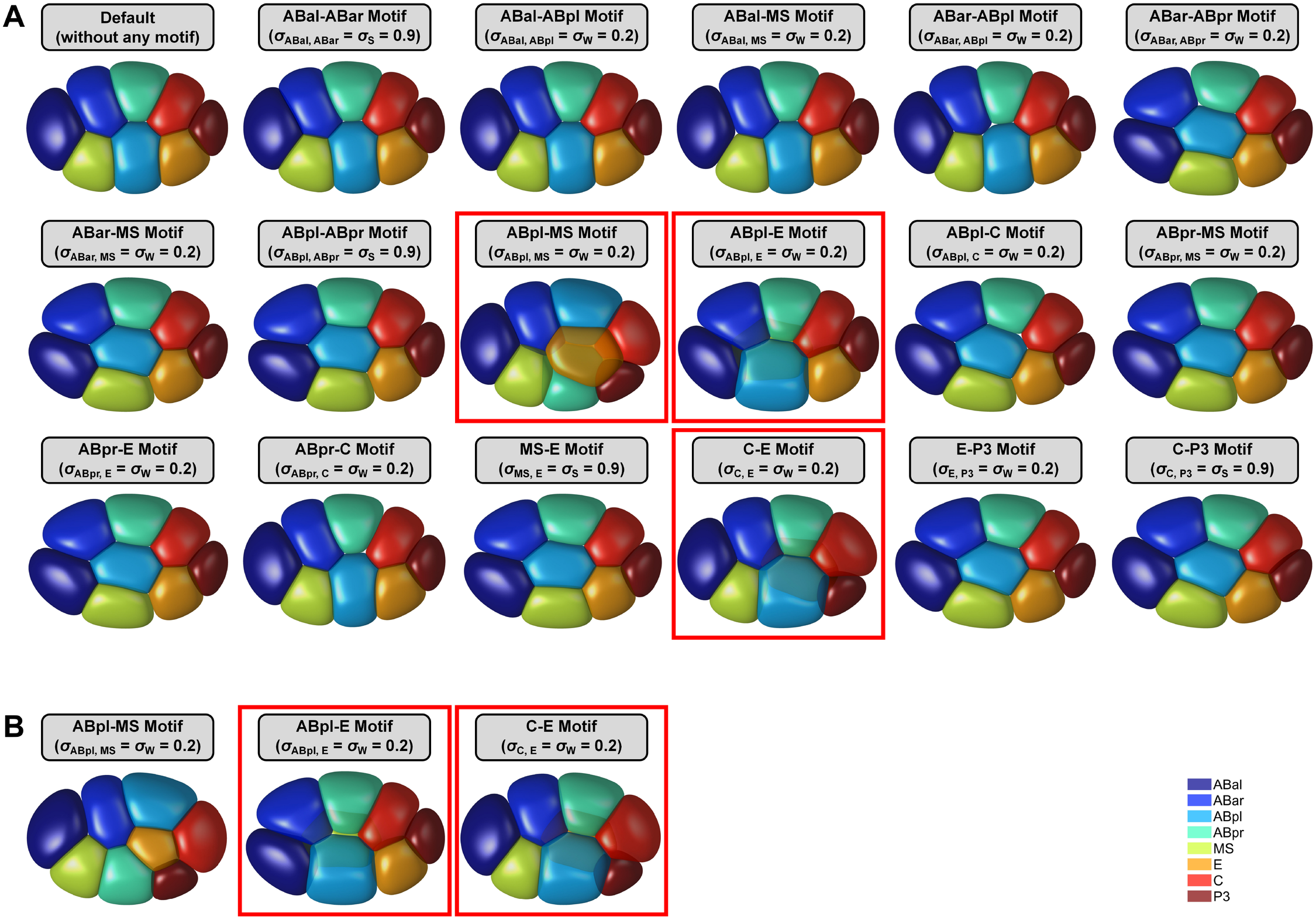

Supplement: S11 Fig — (A). 3D structures at time point 350000. The ones with ABpl-MS, ABpl-E, and C-E motifs are three-dimensional and highlighted with red rectangles. (B). 3D structures at time point 450000. The ones with ABpl-E and C-E motifs are three-dimensional and highlighted with red rectangles. The relationship between cell identity and color is listed in the bottom right corner. (TIF) [file pcbi.1009755.s011.tif]

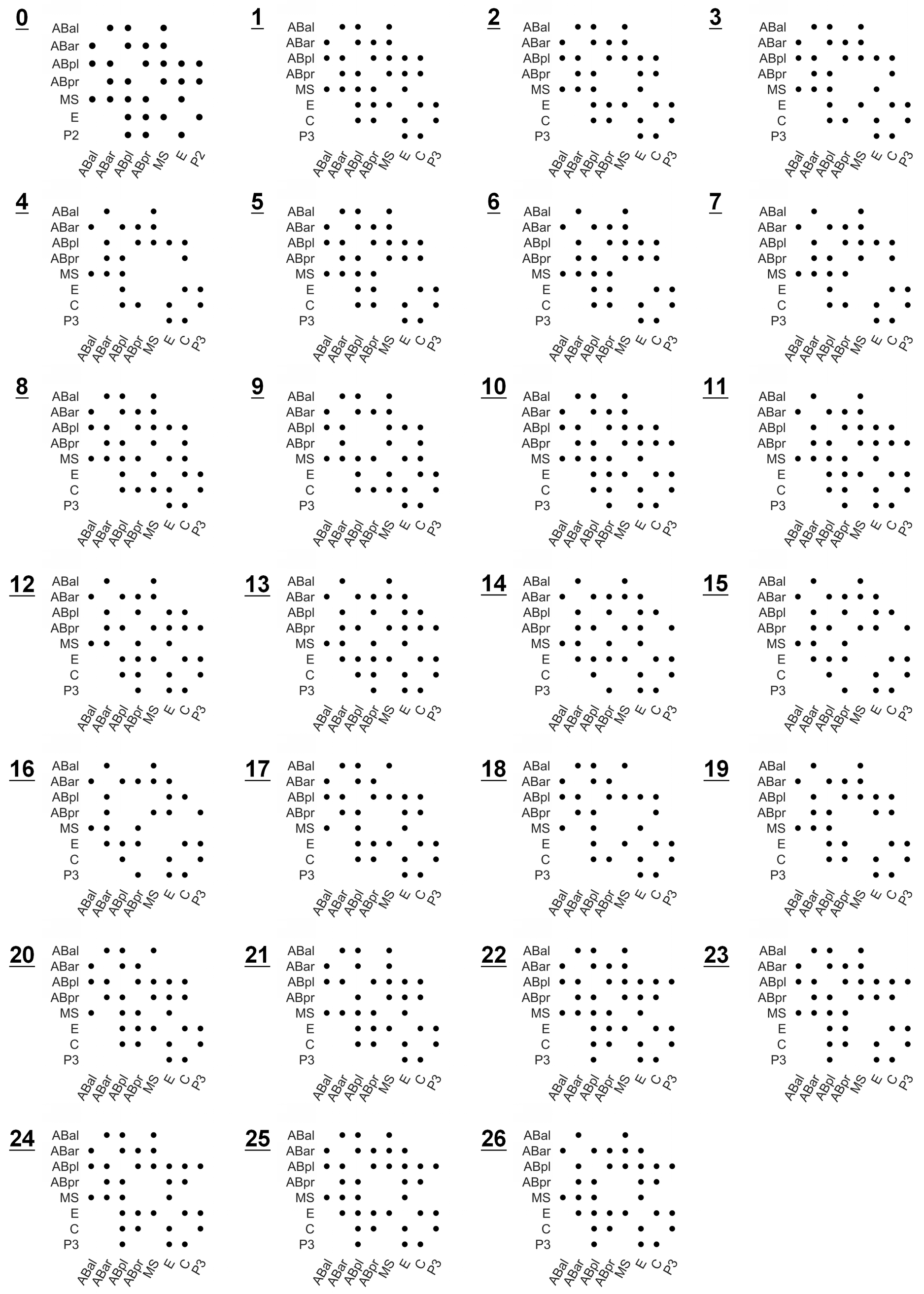

Supplement: S12 Fig — The Topologies 0 ~ 26 corresponding to Fig 6A are listed from the top left corner to the bottom right corner. For each map, black dots represent the contacted cell pairs. (TIF) [file pcbi.1009755.s012.tif]

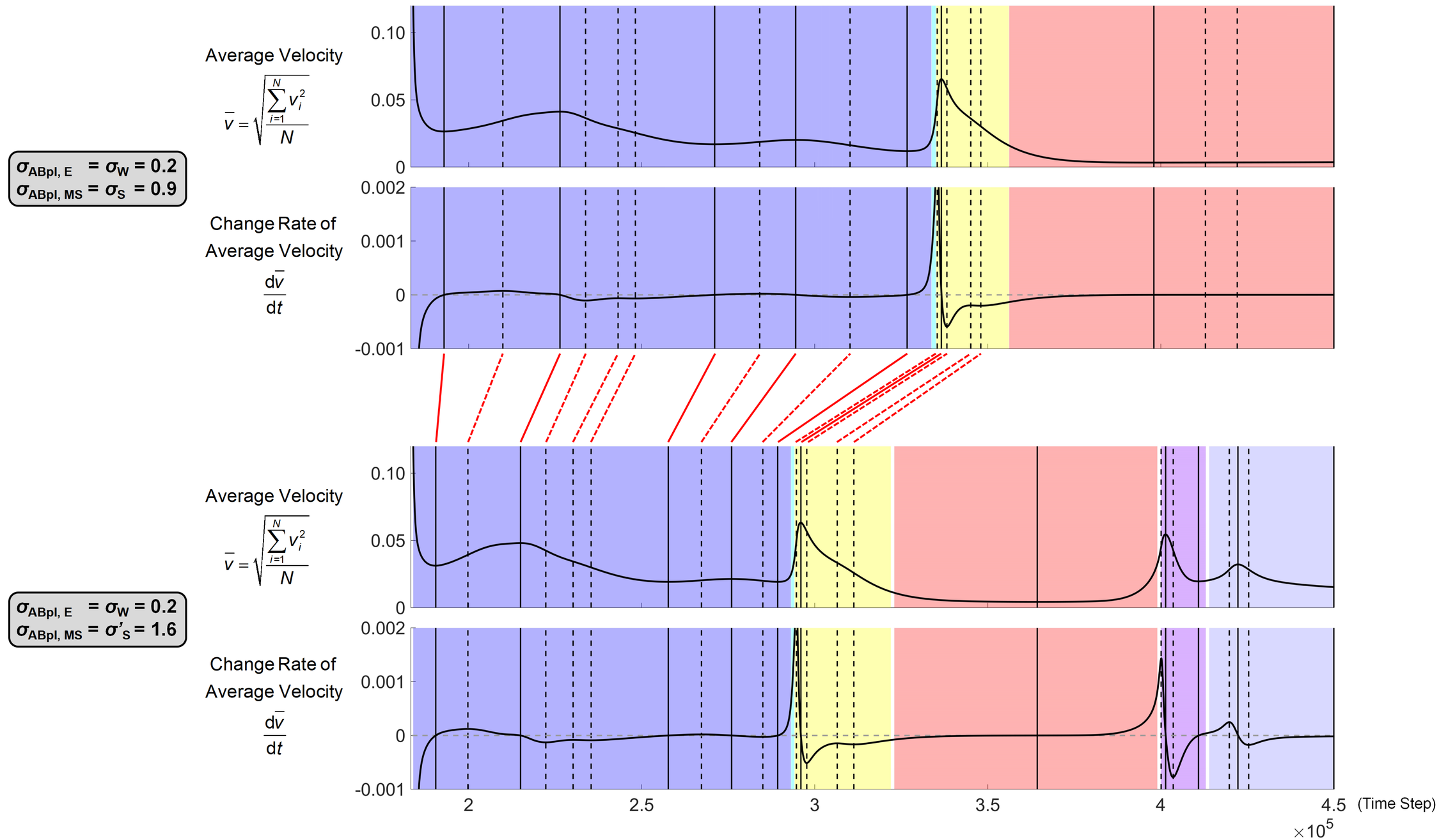

Supplement: S13 Fig — A total of 16 pairs of critical time points in the curves of average velocity and its change rate are aligned and connected between the two panels (embryos), using solid and dashed red lines respectively. (TIF) [file pcbi.1009755.s013.tif]

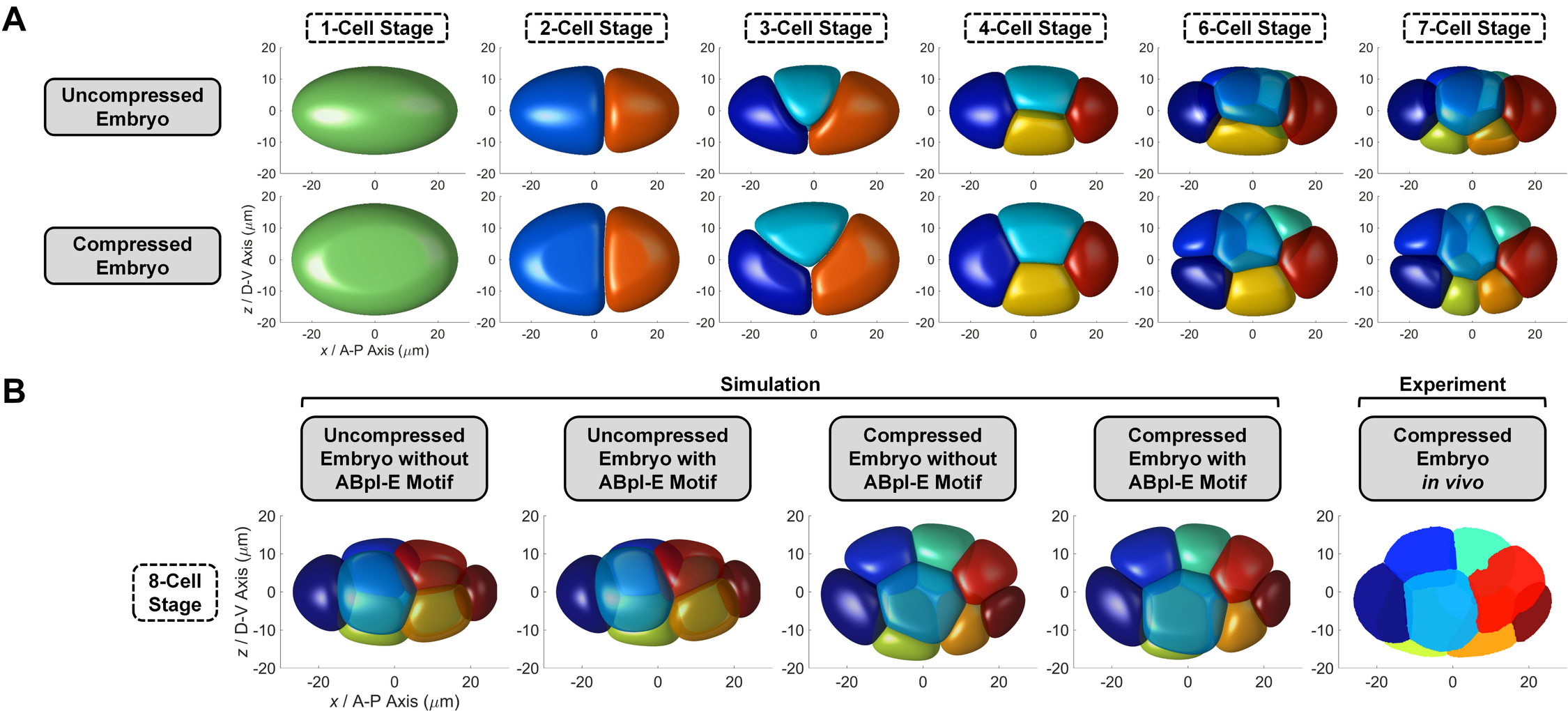

Supplement: S14 Fig — (A). Embryo morphology of uncompressed (1st row) and compressed (2nd row) embryos from 1- to 7-cell stages, without any attraction motif added. (B). Embryo morphology in uncompressed (1st and 2nd columns) and compressed (3rd and 4th columns) embryos at 8-cell stage, with or without the ABpl-E motif, revealing that the ABpl-E motif is essential for the compressed embryo to reach the correct structure as seen in vivo, but not for the uncompressed one. (TIF) [file pcbi.1009755.s014.tif]

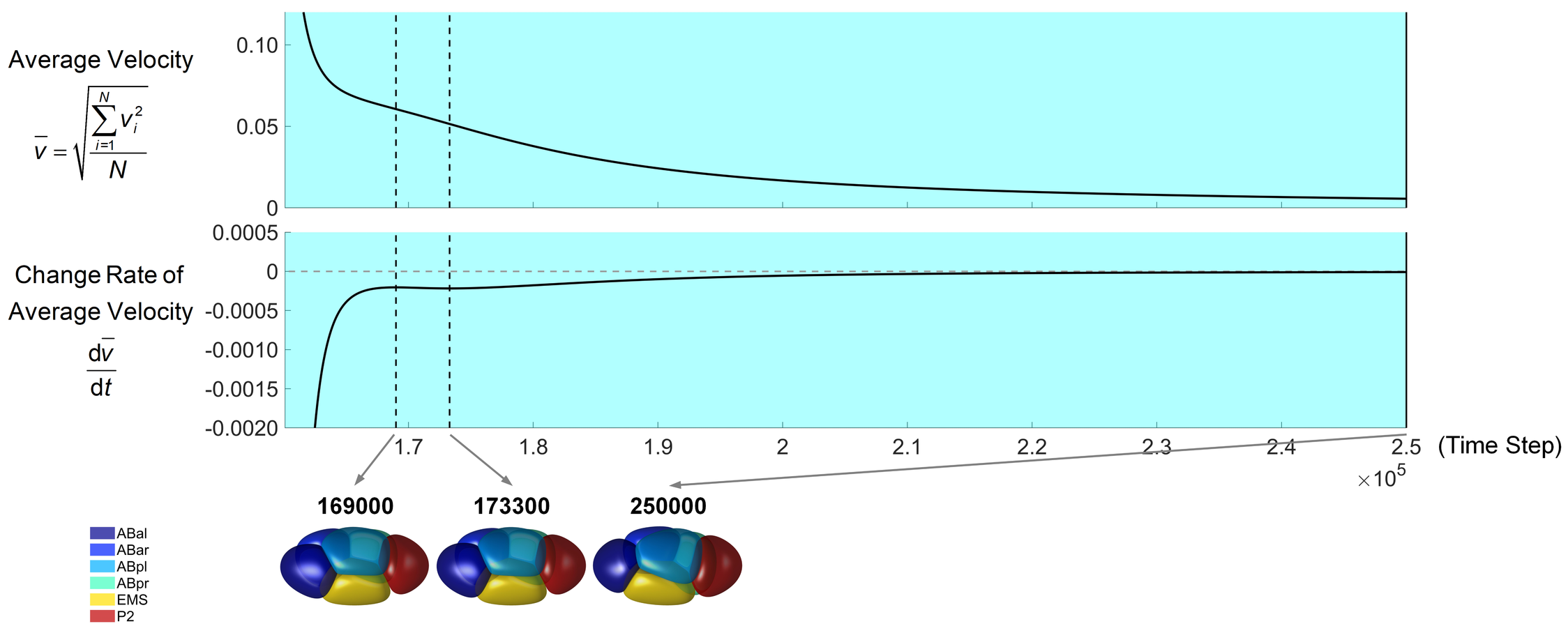

Supplement: S15 Fig — The curves of average velocity (upper) and its change rate (lower) are illustrated side by side. The solid and dashed vertical black lines denote the extreme points in the two curves respectively, while the 3D structures at those time points are illustrated on bottom, pointed by gray arrows originating from their corresponding lines. The last structure in the bottom right is the system’s terminal state approaching mechanical equilibrium. The relationship between cell identity and color is listed in the bottom left corner. (TIF) [file pcbi.1009755.s015.tif]

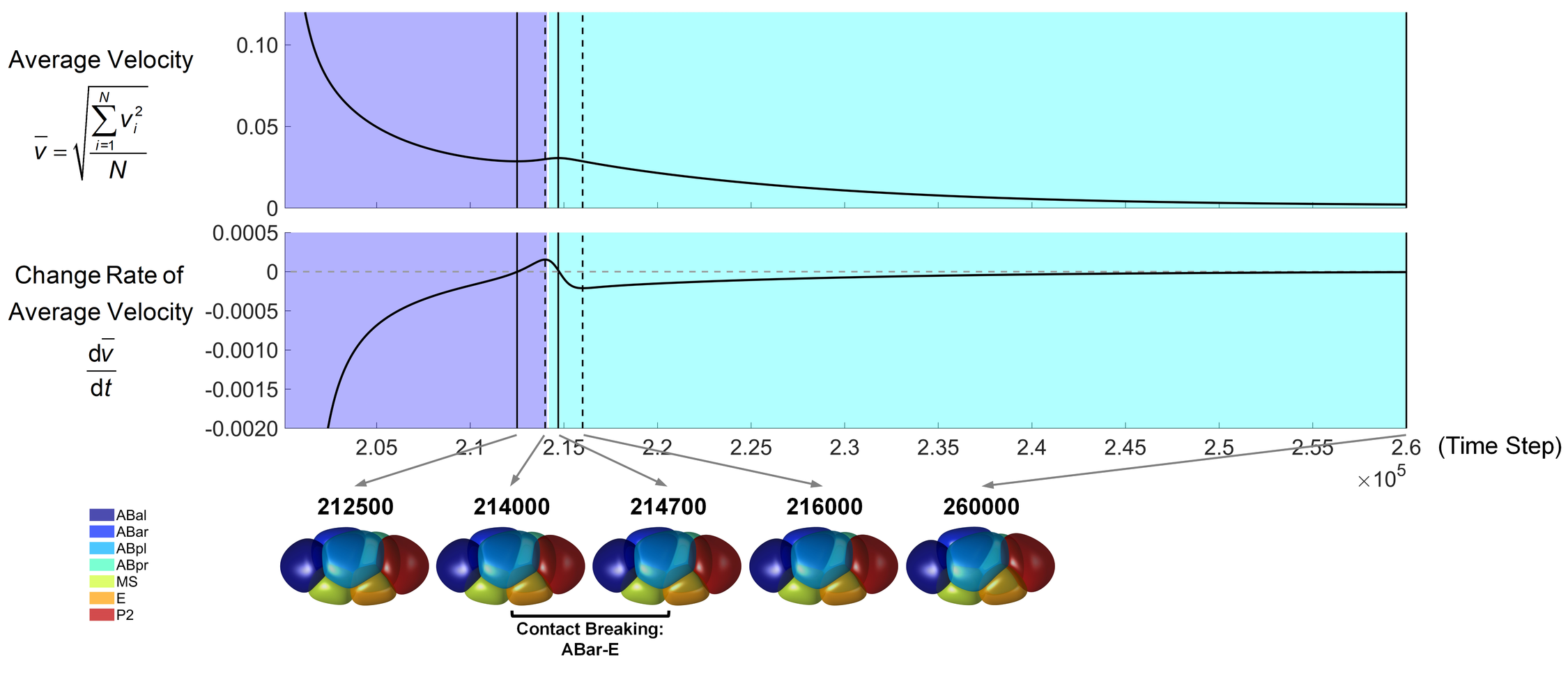

Supplement: S16 Fig — The curves of average velocity (upper) and its change rate (lower) are illustrated side by side. The solid and dashed vertical black lines denote the extreme points in the two curves respectively, while the 3D structures at those time points are illustrated on bottom, pointed by gray arrows originating from their corresponding lines. The last structure in the bottom right is the system’s terminal state approaching mechanical equilibrium. The change of cell-cell contact map is illustrated by different colors in the background, while the detail is written between two consecutive structures. The relationship between cell identity and color is listed in the bottom left corner. (TIF) [file pcbi.1009755.s016.tif]

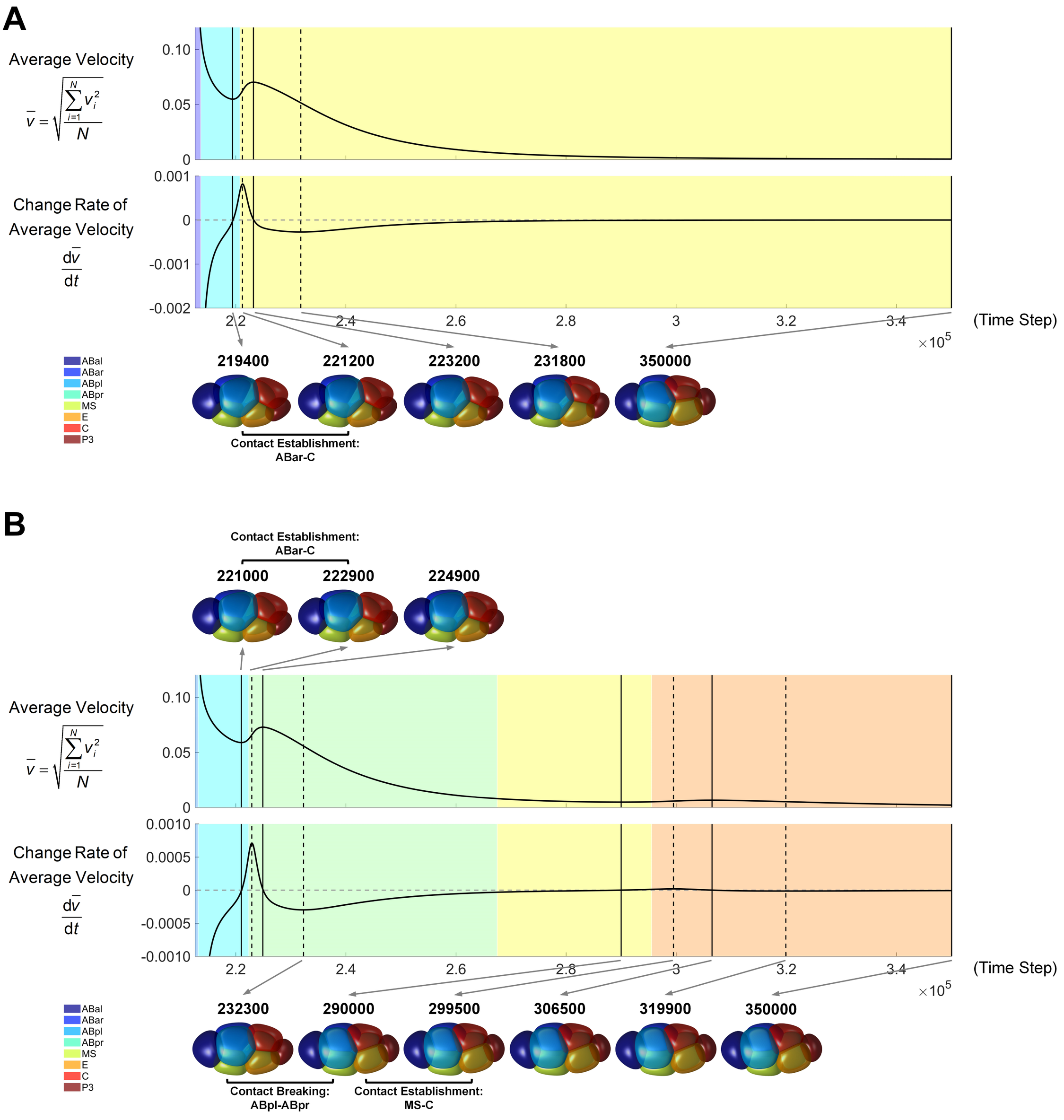

Supplement: S17 Fig — (A). The upper panel, without attraction motif on ABpl-E contact, i.e., σABpl, E = σS. (B). The lower panel, with attraction motif on ABpl-E contact, i.e., σABpl, E = σW. For each panel, the curves of average velocity (upper) and its change rate (lower) are illustrated side by side. The solid and dashed vertical black lines denote the extreme points in the two curves respectively, while the 3D structures at those time points are illustrated on top and bottom, pointed by gray arrows originating from their corresponding lines. The last structure in the bottom right is the system’s terminal state approaching mechanical equilibrium. The change of cell-cell contact map is illustrated by different colors in the background, while the detail is written between two consecutive structures. The relationship between cell identity and color is listed in the bottom left corner. (TIF) [file pcbi.1009755.s017.tif]

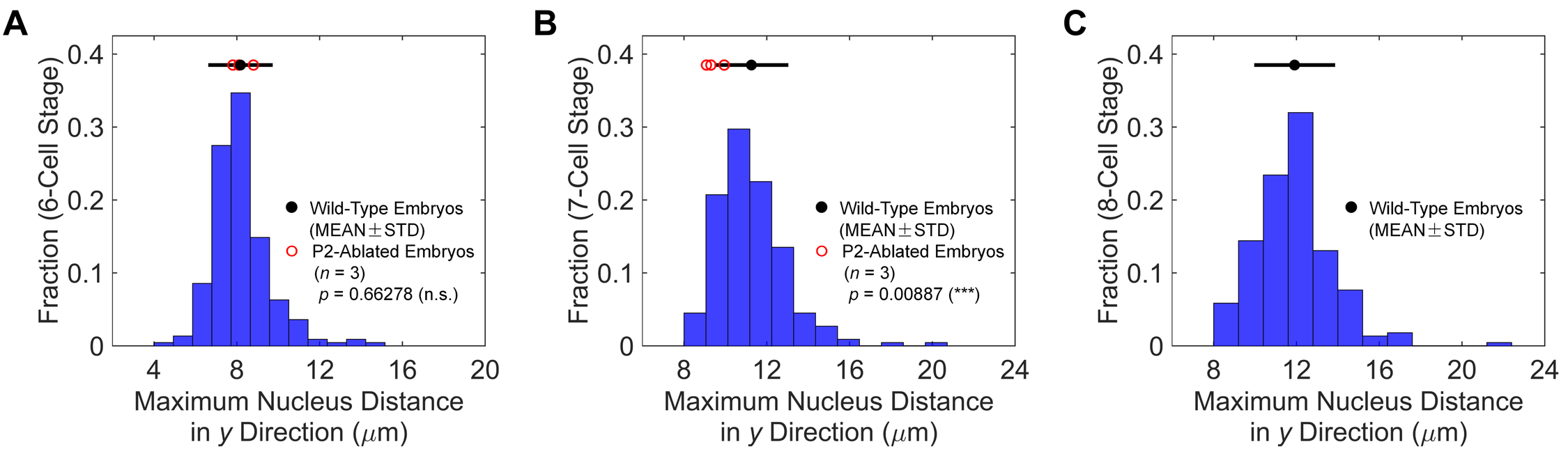

Supplement: S18 Fig — The significance level is obtained by one-tailed Wilcoxon rank-sum test. (A). Distribution at the last time point of 6-cell stage. (B). Distribution at the last time point of 7-cell stage. (C). Distribution at the last time point of 8-cell stage. (TIF) [file pcbi.1009755.s018.tif]

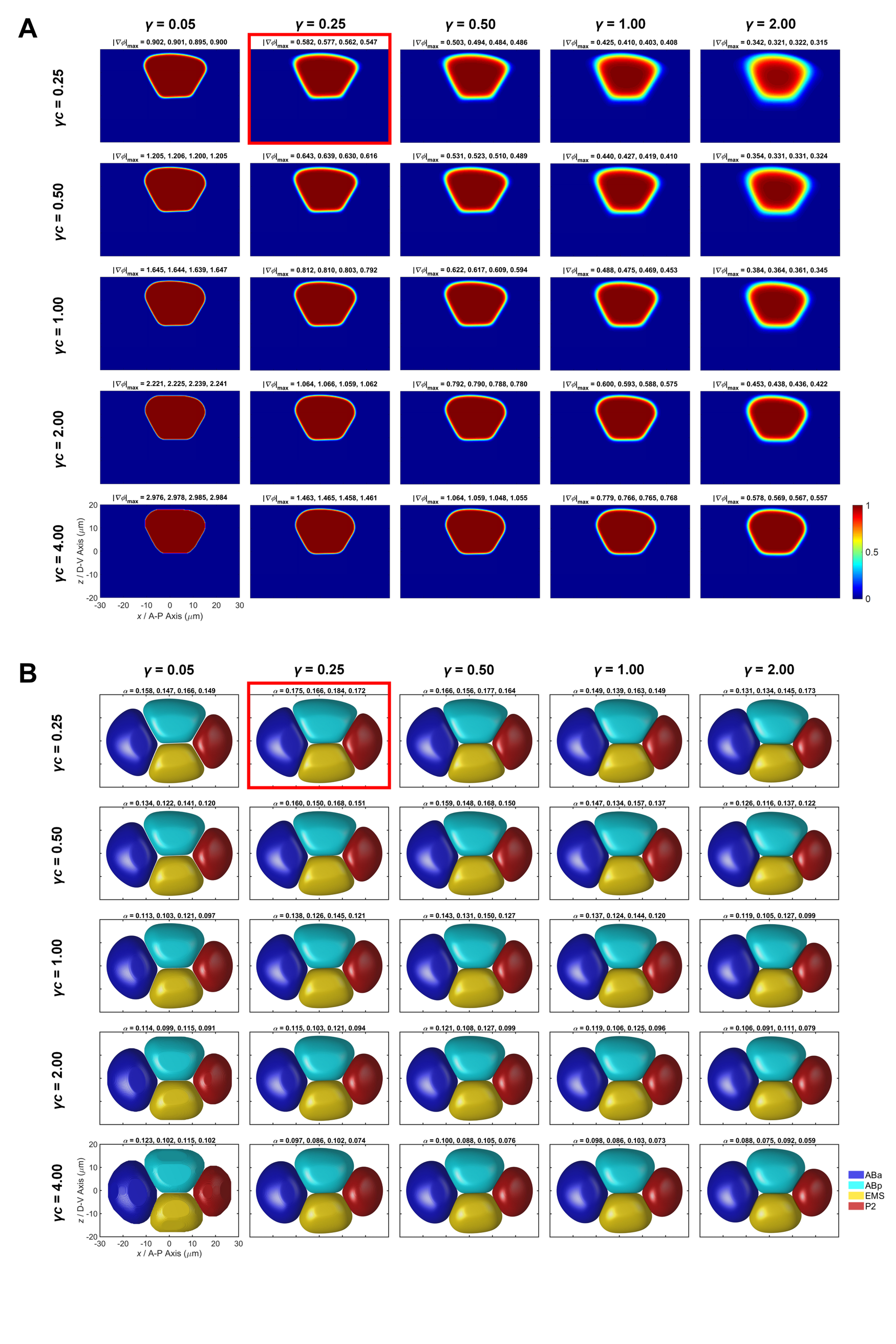

Supplement: S19 Fig — The optimum fitting result (γ = 0.25, c = 1.0) is highlighted by red rectangles. (A). Phase-field distribution at 4-cell stage, with interface transition quantified by maximum gradient |∇ϕ|max. (B). Embryo morphology at 4-cell stage, with cell deformation quantified by coefficient α (Eqs 12 and 13). (TIF) [file pcbi.1009755.s019.tif]

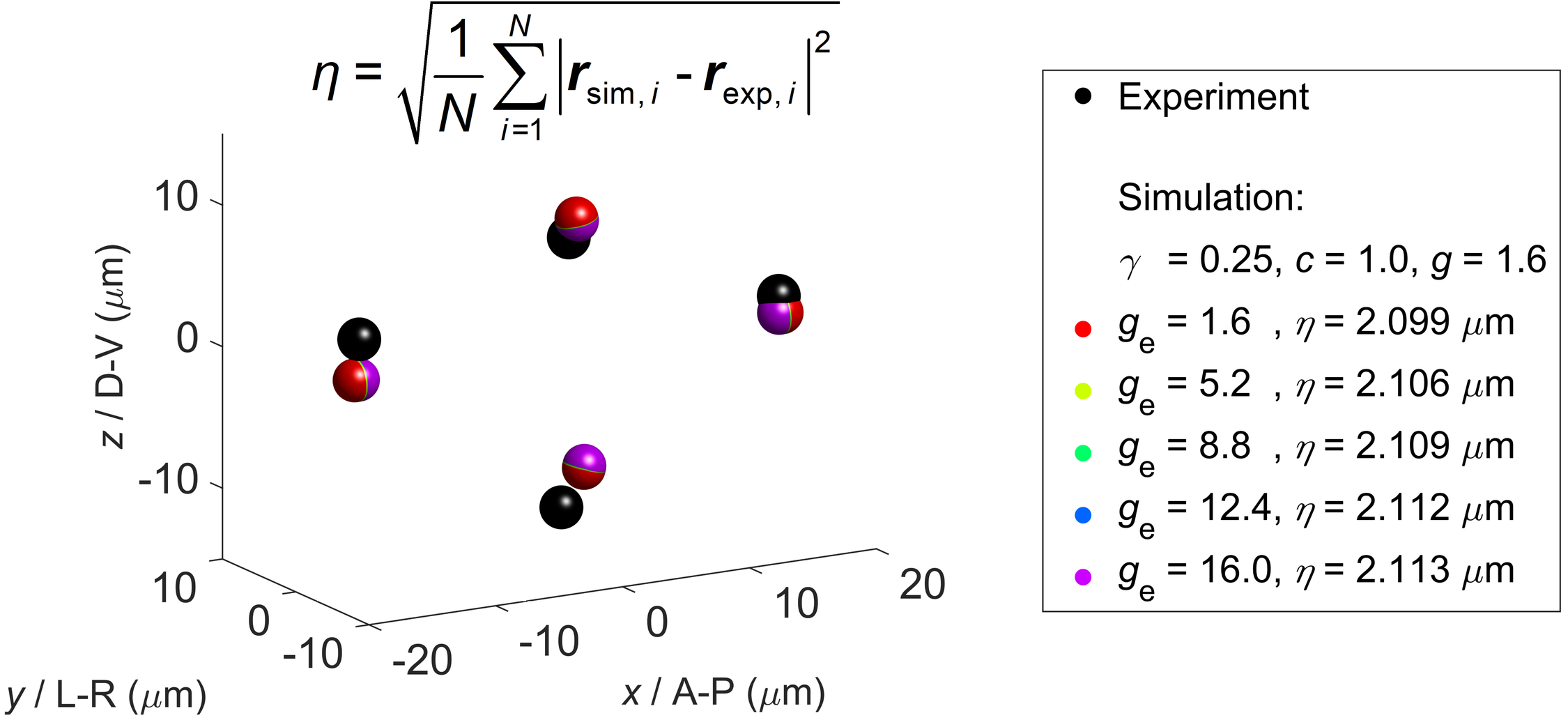

Supplement: S20 Fig — The average positional variation η is used to evaluate the deviation between structures in simulation and experiment (Eq 14). (TIF) [file pcbi.1009755.s020.tif]
