# Supplementary material for: Computable early Caenorhabditis elegans embryo with a phase field model
Source: PLoS Comput Biol. 2022 Jan 14;18(1):e1009755. doi: 10.1371/journal.pcbi.1009755 (PMC8794267; doi:10.1371/journal.pcbi.1009755)
Supplement: S1 Table — (DOCX) [file pcbi.1009755.s021.docx]

**S1 Table. Information of the embryos collected from datasets produced previously.**

| Group | Embryo Number | Original Reference of Strain | Original Reference of Dataset | Nucleus Marker | Membrane Marker | Usage |
| --- | --- | --- | --- | --- | --- | --- |
| 1 | 1 | [1] (RW10425) | [2] | √ | × | 1. Division Orientation of the Cells   before 4-Cell Stage (i.e., P0, AB, P1) |
| 2 | 13 | [1] (RW10112) | [3] | √ | × | 1. Division Orientation of the Cells   since 4-Cell Stage (i.e., ABa, ABp, EMS, P2) |
| 3 | 4 | [3] (ZZY0655) | [3] | √ | √ | 1. Division Orientation of the Cells   since 4-Cell Stage (i.e., ABa, ABp, EMS, P2)   1. Morphology of the Cells   since 4-Cell Stage  (i.e., ABa, ABp, EMS, P2,  ABal, ABar, ABpl, ABpr,  MS, E, C, P3) |

**Reference**

1. Murray JI, Boyle TJ, Preston E, Vafeados D, Mericle B, Weisdepp P, et al. Multidimensional regulation of gene expression in the *C. elegans* embryo. Genome Res. 2012, 22(7):1282-94. https://doi.org/10.1101/gr.131920.111, PMID: 22508763
2. Guan G, Wong MK, Ho VWS, An X, Chan LY, Tian B, et al. System-level quantification and phenotyping of early embryonic morphogenesis of *Caenorhabditis elegans*. bioRxiv. 2019, 776062. Preprint at https://www.biorxiv.org/content/10.1101/776062v1
3. Cao J, Guan G, Wong MK, Chan LY, Tang C, Zhao Z, et al. Establishment of morphological atlas of *Caenorhabditis elegans* embryo with cellular resolution using deep-learning-based 4D segmentation. bioRxiv. 2019, 797688. Preprint at https://www.biorxiv.org/content/10.1101/797688v1
