# Supplementary material for: Computable early Caenorhabditis elegans embryo with a phase field model
Source: PLoS Comput Biol. 2022 Jan 14;18(1):e1009755. doi: 10.1371/journal.pcbi.1009755 (PMC8794267; doi:10.1371/journal.pcbi.1009755)
Supplement: S2 Table — (DOCX) [file pcbi.1009755.s022.docx]

**S2 Table. Volume and division orientation of the cells up to 8-cell stage.**

| Cell Identity | Cell Volume (μm^3^) | Cell Division Orientation (Normalized, [*x*, *y*, *z*]) |
| --- | --- | --- |
| P0 | 20107.335 | [-0.992,-0.123,0.010] |
| AB | 11938.845 | [0.767,0.078,0.637] |
| P1 | 8168.490 | [0.954,0.040,0.297] |
| ABa | 6020.879 | [0.313,0.684,0.659] |
| ABp | 5917.966 | [0.376,0.913,0.160] |
| EMS | 4690.054 | [0.978,-0.196,-0.073] |
| P2 | 3478.436 | [0.255,0.155,-0.954] |
| ABal | 2879.988 | [0.424,0.412,-0.807] |
| ABar | 3010.672 | [0.478,-0.839,0.260] |
| ABpl | 3240.075 | [0.689,0.354,-0.632] |
| ABpr | 2642.929 | [0.349,0.123,-0.929] |
| MS | 2389.470 | [0.920,0.062,0.388] |
| E | 2080.275 | [0.938,-0.131,0.320] |
| C | 2365.737 | [0.976,0.103,0.191] |
| P3 | 1116.712 | [-0.266,0.201,-0.943] |

Note: For P0, AB and P1, cell volume is calculated with the ones of their progenies (i.e., ABa, ABp, EMS, P2). Cell division orientations of P0, AB and P1 are measured using a wild-type embryo imaged since 1-cell stage (S1 Table) [1]. For the others appearing since 4-cell stage, cell volumes are the averages obtained from 4 wild-type embryos with membrane marker, while cell division orientations are the averages obtained from all the 17 wild-type embryos with nucleus marker (S1 Table) [2].

**Reference**

1. Guan G, Wong MK, Ho VWS, An X, Chan LY, Tian B, et al. System-level quantification and phenotyping of early embryonic morphogenesis of *Caenorhabditis elegans*. bioRxiv. 2019, 776062. Preprint at https://www.biorxiv.org/content/10.1101/776062v1
2. Cao J, Guan G, Wong MK, Chan LY, Tang C, Zhao Z, et al. Establishment of morphological atlas of *Caenorhabditis elegans* embryo with cellular resolution using deep-learning-based 4D segmentation. bioRxiv. 2019, 797688. Preprint at https://www.biorxiv.org/content/10.1101/797688v1
