# Supplementary material for: Computable early Caenorhabditis elegans embryo with a phase field model
Source: PLoS Comput Biol. 2022 Jan 14;18(1):e1009755. doi: 10.1371/journal.pcbi.1009755 (PMC8794267; doi:10.1371/journal.pcbi.1009755)
Supplement: S3 Table — (DOCX) [file pcbi.1009755.s023.docx]

**S3 Table. Cell surface area, cell-cell contact relationship and area at 4-cell stage.**

| Cell  Identity |  | ABa | ABp | EMS | P2 |
| --- | --- | --- | --- | --- | --- |
|  | Surface Area | 30818 | 31141 | 31341 | 23667 |
|  |  | 32121 | 32105 | 32590 | 23450 |
|  |  | 29655 | 29935 | 29408 | 22699 |
|  |  | 29104 | 32103 | 28542 | 21670 |
| ABa | 30818 | 0 | 6591 | 5718 | 0 |
|  | 32121 | 0 | 6275 | 5075 | 0 |
|  | 29655 | 0 | 5859 | 4215 | 0 |
|  | 29104 | 0 | 5902 | 4692 | 0 |
| ABp | 31141 | 6591 | 0 | 4472 | 5421 |
|  | 32105 | 6275 | 0 | 5361 | 4957 |
|  | 29935 | 5859 | 0 | 4876 | 4316 |
|  | 32103 | 5902 | 0 | 4417 | 4523 |
| EMS | 31341 | 5718 | 4472 | 0 | 2620 |
|  | 32590 | 5075 | 5361 | 0 | 1845 |
|  | 29408 | 4215 | 4876 | 0 | 2372 |
|  | 28542 | 4692 | 4417 | 0 | 2385 |
| P2 | 23667 | 0 | 5421 | 2620 | 0 |
|  | 23450 | 0 | 4957 | 1845 | 0 |
|  | 22699 | 0 | 4316 | 2372 | 0 |
|  | 21670 | 0 | 4523 | 2385 | 0 |

Note: Cell surface area is quantified by the total number of pixels surrounding a cell, while cell-cell contact area is quantified by the total number of pixels adjacent to two cells (sample size = 4; spatial resolution ≈ 0.225 μm / pixel in three orthogonal coordinates). “0” means that the two independent cells don’t contact each other at all (S1 Table) [1].

**Reference**

1. Cao J, Guan G, Wong MK, Chan LY, Tang C, Zhao Z, et al. Establishment of morphological atlas of *Caenorhabditis elegans* embryo with cellular resolution using deep-learning-based 4D segmentation. bioRxiv. 2019, 797688. Preprint at https://www.biorxiv.org/content/10.1101/797688v1
