# Supplementary material for: Computable early Caenorhabditis elegans embryo with a phase field model
Source: PLoS Comput Biol. 2022 Jan 14;18(1):e1009755. doi: 10.1371/journal.pcbi.1009755 (PMC8794267; doi:10.1371/journal.pcbi.1009755)
Supplement: S4 Table — (DOCX) [file pcbi.1009755.s024.docx]

**S4 Table. Cell surface area, cell-cell contact relationship and area at 6-cell stage.**

| Cell  Identity |  | ABal | ABar | ABpl | ABpr | EMS | P2 |
| --- | --- | --- | --- | --- | --- | --- | --- |
|  | Surface  Area | 18213 | 21100 | 22378 | 17609 | 26983 | 23360 |
|  |  | NaN | NaN | NaN | NaN | 29589 | 21621 |
|  |  | 17076 | 20312 | 23168 | 17280 | 24715 | 21648 |
|  |  | 16279 | 18435 | 22993 | 17502 | 24186 | 19801 |
| ABal | 18213 | 0 | 893 | 2162 | 0 | 3830 | 0 |
|  | NaN | NaN | NaN | NaN | NaN | NaN | NaN |
|  | 17076 | 0 | 1107 | 1667 | 0 | 2716 | 0 |
|  | 16279 | 0 | 1553 | 986 | 0 | 2821 | 0 |
| ABar | 21100 | 893 | 0 | 4502 | 2761 | 2238 | 0 |
|  | NaN | NaN | NaN | NaN | NaN | NaN | NaN |
|  | 20312 | 1107 | 0 | 4702 | 1711 | 1729 | 0 |
|  | 18435 | 1553 | 0 | 4030 | 1188 | 1403 | 0 |
| ABpl | 22378 | 2162 | 4502 | 0 | 590 | 2528 | 2182 |
|  | NaN | NaN | NaN | NaN | NaN | NaN | NaN |
|  | 23168 | 1667 | 4702 | 0 | 906 | 3292 | 1181 |
|  | 22993 | 986 | 4030 | 0 | 2231 | 2584 | 1184 |
| ABpr | 17609 | 0 | 2761 | 590 | 0 | 1826 | 3604 |
|  | NaN | NaN | NaN | NaN | NaN | NaN | NaN |
|  | 17280 | 0 | 1711 | 906 | 0 | 1527 | 3122 |
|  | 17502 | 0 | 1188 | 2231 | 0 | 1420 | 2639 |
| EMS | 26983 | 3830 | 2238 | 2528 | 1826 | 0 | 3240 |
|  | 29589 | NaN | NaN | NaN | NaN | 0 | 2236 |
|  | 24715 | 2716 | 1729 | 3292 | 1527 | 0 | 3066 |
|  | 24186 | 2821 | 1403 | 2584 | 1420 | 0 | 2934 |
| P2 | 23360 | 0 | 0 | 2182 | 3604 | 3240 | 0 |
|  | 21621 | NaN | NaN | NaN | NaN | 2236 | 0 |
|  | 21648 | 0 | 0 | 1181 | 3122 | 3066 | 0 |
|  | 19801 | 0 | 0 | 1184 | 2639 | 2934 | 0 |

Note: Cell surface area is quantified by the total number of pixels surrounding a cell, while cell-cell contact area is quantified by the total number of pixels adjacent to two cells (sample size = 4; spatial resolution ≈ 0.225 μm / pixel in three orthogonal coordinates). “NaN” represents a cell that has its own nucleus but hasn’t finished cytokinesis with its sister, which together share the same membrane boundary. “0” means that the two independent cells don’t contact each other at all (S1 Table) [1].

**Reference**

1. Cao J, Guan G, Wong MK, Chan LY, Tang C, Zhao Z, et al. Establishment of morphological atlas of *Caenorhabditis elegans* embryo with cellular resolution using deep-learning-based 4D segmentation. bioRxiv. 2019, 797688. Preprint at https://www.biorxiv.org/content/10.1101/797688v1
