# Supplementary material for: Computable early Caenorhabditis elegans embryo with a phase field model
Source: PLoS Comput Biol. 2022 Jan 14;18(1):e1009755. doi: 10.1371/journal.pcbi.1009755 (PMC8794267; doi:10.1371/journal.pcbi.1009755)
Supplement: S6 Table — (DOCX) [file pcbi.1009755.s026.docx]

**S6 Table. Cell surface area, cell-cell contact relationship and area at 8-cell stage.**

| Cell Identity |  | ABal | ABar | ABpl | ABpr | MS | E | C | P3 |
| --- | --- | --- | --- | --- | --- | --- | --- | --- | --- |
|  | Surface  Area | 18405 | 19431 | 19462 | 16561 | 20681 | 18184 | 19945 | 9994 |
|  |  | 18315 | 19500 | 21850 | 17480 | 23034 | 15782 | 19032 | 10357 |
|  |  | 17130 | 18899 | 19348 | 16548 | 21096 | 14890 | 19749 | 9441 |
|  |  | 15950 | 17385 | 19825 | 16355 | 17064 | 15405 | 17163 | 9163 |
| ABal | 18405 | 0 | 1679 | 2795 | 0 | 2998 | 0 | 0 | 0 |
|  | 18315 | 0 | 1766 | 2692 | 0 | 2524 | 0 | 0 | 0 |
|  | 17130 | 0 | 1855 | 2642 | 0 | 1979 | 0 | 0 | 0 |
|  | 15950 | 0 | 1315 | 2297 | 0 | 2557 | 0 | 0 | 0 |
| ABar | 19431 | 1679 | 0 | 2170 | 2795 | 2367 | 14 | 760 | 0 |
|  | 19500 | 1766 | 0 | 2610 | 3192 | 1558 | 0 | 3 | 0 |
|  | 18899 | 1855 | 0 | 2448 | 3042 | 1340 | 0 | 202 | 0 |
|  | 17385 | 1315 | 0 | 2603 | 2719 | 861 | 0 | 0 | 0 |
| ABpl | 19462 | 2795 | 2170 | 0 | 0 | 2119 | 1770 | 2011 | 0 |
|  | 21850 | 2692 | 2610 | 0 | 195 | 4173 | 1664 | 1094 | 0 |
|  | 19348 | 2642 | 2448 | 0 | 0 | 3736 | 816 | 1323 | 0 |
|  | 19825 | 2297 | 2603 | 0 | 612 | 2633 | 1361 | 1547 | 0 |
| ABpr | 16561 | 0 | 2795 | 0 | 0 | 897 | 1646 | 2911 | 0 |
|  | 17480 | 0 | 3192 | 195 | 0 | 1224 | 1153 | 3760 | 0 |
|  | 16548 | 0 | 3042 | 0 | 0 | 1247 | 937 | 3439 | 0 |
|  | 16355 | 0 | 2719 | 612 | 0 | 0 | 855 | 3162 | 0 |
| MS | 20681 | 2998 | 2367 | 2119 | 897 | 0 | 2407 | 0 | 0 |
|  | 23034 | 2524 | 1558 | 4173 | 1224 | 0 | 1891 | 11 | 0 |
|  | 21096 | 1979 | 1340 | 3736 | 1247 | 0 | 2045 | 212 | 0 |
|  | 17064 | 2557 | 861 | 2633 | 0 | 0 | 898 | 0 | 0 |
| E | 18184 | 0 | 14 | 1770 | 1646 | 2407 | 0 | 2246 | 2408 |
|  | 15782 | 0 | 0 | 1664 | 1153 | 1891 | 0 | 1038 | 2206 |
|  | 14890 | 0 | 0 | 816 | 937 | 2045 | 0 | 2108 | 2037 |
|  | 15405 | 0 | 0 | 1361 | 855 | 898 | 0 | 1677 | 2099 |
| C | 19945 | 0 | 760 | 2011 | 2911 | 0 | 2246 | 0 | 1866 |
|  | 19032 | 0 | 3 | 1094 | 3760 | 11 | 1038 | 0 | 2291 |
|  | 19749 | 0 | 202 | 1323 | 3439 | 212 | 2108 | 0 | 1840 |
|  | 17163 | 0 | 0 | 1547 | 3162 | 0 | 1677 | 0 | 1688 |
| P3 | 9994 | 0 | 0 | 0 | 0 | 0 | 2408 | 1866 | 0 |
|  | 10357 | 0 | 0 | 0 | 0 | 0 | 2206 | 2291 | 0 |
|  | 9441 | 0 | 0 | 0 | 0 | 0 | 2037 | 1840 | 0 |
|  | 9163 | 0 | 0 | 0 | 0 | 0 | 2099 | 1688 | 0 |

Note: Cell surface area is quantified by the total number of pixels surrounding a cell, while cell-cell contact area is quantified by the total number of pixels adjacent to two cells (sample size = 4; spatial resolution ≈ 0.225 μm / pixel in three orthogonal coordinates). “0” means that the two independent cells don’t contact each other at all (S1 Table) [1].

**Reference**

1. Cao J, Guan G, Wong MK, Chan LY, Tang C, Zhao Z, et al. Establishment of morphological atlas of *Caenorhabditis elegans* embryo with cellular resolution using deep-learning-based 4D segmentation. bioRxiv. 2019, 797688. Preprint at https://www.biorxiv.org/content/10.1101/797688v1
