# Supplementary material for: Computable early Caenorhabditis elegans embryo with a phase field model
Source: PLoS Comput Biol. 2022 Jan 14;18(1):e1009755. doi: 10.1371/journal.pcbi.1009755 (PMC8794267; doi:10.1371/journal.pcbi.1009755)
Supplement: S7 Table — (DOCX) [file pcbi.1009755.s027.docx]

**S7 Table. Comparison between simulation (*σ* = 0.0, 0.3, 0.6, 0.9, 1.2, 1.5 for all contacts) and experiment**

**at 4-cell stage.**

| *σ* |  | Area in Simulation $s_{0}$  (Pixel Number*Spatial Resolution^2^, μm^2^) | | | | | $\delta=\left\vert\frac{s_{0}-s}{s} \right\vert$ | | | | | $\bar{\delta}$ |
| --- | --- | --- | --- | --- | --- | --- | --- | --- | --- | --- | --- | --- |
|  |  | Contact | | | | Surface | Contact | | | | Surface |  |
|  |  | ABa | ABp | EMS | P2 |  | ABa | ABp | EMS | P2 |  |  |
| 0.0 | ABa | 0.00 | 123.50 | 82.63 | 0.00 | 1494.62 | NaN | 0.625 | 0.686 | NaN | 0.030 | 0.3641 |
|  | ABp | 123.50 | 0.00 | 45.15 | 111.05 | 1546.50 | 0.625 | NaN | 0.823 | 0.568 | 0.025 |  |
|  | EMS | 82.63 | 45.15 | 0.00 | 93.25 | 1317.05 | 0.686 | 0.823 | NaN | 0.244 | 0.146 |  |
|  | P2 | 0.00 | 111.05 | 93.25 | 0.00 | 1006.85 | NaN | 0.568 | 0.244 | NaN | 0.130 |  |
| 0.3 | ABa | 0.00 | 244.67 | 188.39 | 0.00 | 1492.99 | NaN | 0.257 | 0.285 | NaN | 0.031 | 0.2054 |
|  | ABp | 244.67 | 0.00 | 158.08 | 206.00 | 1547.63 | 0.257 | NaN | 0.382 | 0.198 | 0.024 |  |
|  | EMS | 188.39 | 158.08 | 0.00 | 171.98 | 1316.48 | 0.285 | 0.382 | NaN | 0.395 | 0.147 |  |
|  | P2 | 0.00 | 206.00 | 171.98 | 0.00 | 1006.41 | NaN | 0.198 | 0.395 | NaN | 0.131 |  |
| 0.6 | ABa | 0.00 | 291.27 | 229.77 | 0.00 | 1494.37 | NaN | 0.115 | 0.128 | NaN | 0.030 | 0.1705 |
|  | ABp | 291.27 | 0.00 | 204.87 | 245.87 | 1550.96 | 0.115 | NaN | 0.199 | 0.043 | 0.022 |  |
|  | EMS | 229.77 | 204.87 | 0.00 | 212.92 | 1322.83 | 0.128 | 0.199 | NaN | 0.727 | 0.142 |  |
|  | P2 | 0.00 | 245.87 | 212.92 | 0.00 | 1008.24 | NaN | 0.043 | 0.727 | NaN | 0.129 |  |
| 0.9 | ABa | 0.00 | 323.21 | 257.94 | 0.00 | 1500.41 | NaN | 0.018 | 0.021 | NaN | 0.026 | 0.1559 |
|  | ABp | 323.21 | 0.00 | 233.54 | 269.95 | 1557.19 | 0.018 | NaN | 0.087 | 0.051 | 0.018 |  |
|  | EMS | 257.94 | 233.54 | 0.00 | 236.62 | 1329.69 | 0.021 | 0.087 | NaN | 0.919 | 0.138 |  |
|  | P2 | 0.00 | 269.95 | 236.62 | 0.00 | 1012.01 | NaN | 0.051 | 0.919 | NaN | 0.126 |  |
| 1.2 | ABa | 0.00 | 339.50 | 272.09 | 0.00 | 1503.74 | NaN | 0.031 | 0.033 | NaN | 0.024 | 0.1723 |
|  | ABp | 339.50 | 0.00 | 253.35 | 288.44 | 1562.03 | 0.031 | NaN | 0.009 | 0.123 | 0.015 |  |
|  | EMS | 272.09 | 253.35 | 0.00 | 253.22 | 1330.75 | 0.033 | 0.009 | NaN | 1.054 | 0.137 |  |
|  | P2 | 0.00 | 288.44 | 253.22 | 0.00 | 1013.20 | NaN | 0.123 | 1.054 | NaN | 0.125 |  |
| 1.5 | ABa | 0.00 | 353.58 | 286.05 | 0.00 | 1505.82 | NaN | 0.074 | 0.086 | NaN | 0.022 | 0.1932 |
|  | ABp | 353.58 | 0.00 | 260.77 | 294.60 | 1561.53 | 0.074 | NaN | 0.020 | 0.147 | 0.015 |  |
|  | EMS | 286.05 | 260.77 | 0.00 | 260.83 | 1333.08 | 0.086 | 0.020 | NaN | 1.115 | 0.136 |  |
|  | P2 | 0.00 | 294.60 | 260.83 | 0.00 | 1015.28 | NaN | 0.147 | 1.115 | NaN | 0.123 |  |
|  |  |  |  |  |  |  |  |  |  |  |  |  |
|  |  | Area in Experiment $s$  (Pixel Number*Spatial Resolution^2^, μm^2^) | | | | |  |  |  |  |  |  |
|  |  | Contact | | | | Surface |  |  |  |  |  |  |
|  |  | ABa | ABp | EMS | P2 |  |  |  |  |  |  |  |
|  | ABa | 0.00 | 329.25 | 263.36 | 0.00 | 1540.24 |  |  |  |  |  |  |
|  | ABp | 329.25 | 0.00 | 255.71 | 256.91 | 1585.63 |  |  |  |  |  |  |
|  | EMS | 263.36 | 255.71 | 0.00 | 123.30 | 1542.56 |  |  |  |  |  |  |
|  | P2 | 0.00 | 256.91 | 123.30 | 0.00 | 1157.87 |  |  |  |  |  |  |

Note: $\bar{\delta}$ is the average of $\delta$ of all the surfaces and interfaces.
