# Supplementary material for: Computable early Caenorhabditis elegans embryo with a phase field model
Source: PLoS Comput Biol. 2022 Jan 14;18(1):e1009755. doi: 10.1371/journal.pcbi.1009755 (PMC8794267; doi:10.1371/journal.pcbi.1009755)
Supplement: S8 Table — (DOCX) [file pcbi.1009755.s028.docx]

**S8 Table. Comparison between simulation (*σ* = 0.9 for ABa-ABp, ABa-EMS, ABp-EMS, ABp-P2 contacts,**

***σ*_EMS, P2_ = 0.0, 0.2, 0.4, 0.6, 0.8) and experiment at 4-cell stage.**

| *σ*_EMS, P2_ |  | Area in Simulation $s_{0}$  (Pixel Number*Spatial Resolution^2^, μm^2^) | | | | | $\delta=\left\vert\frac{s_{0}-s}{s} \right\vert$ | | | | | $\bar{\delta}$ |
| --- | --- | --- | --- | --- | --- | --- | --- | --- | --- | --- | --- | --- |
|  |  | Contact | | | | Surface | Contact | | | | Surface |  |
|  |  | ABa | ABp | EMS | P2 |  | ABa | ABp | EMS | P2 |  |  |
| 0.0 | ABa | 0.00 | 318.56 | 279.76 | 0.00 | 1502.48 | NaN | 0.032 | 0.062 | NaN | 0.025 | 0.1429 |
|  | ABp | 318.56 | 0.00 | 245.61 | 275.17 | 1571.34 | 0.032 | NaN | 0.039 | 0.071 | 0.009 |  |
|  | EMS | 279.76 | 245.61 | 0.00 | 27.42 | 1321.01 | 0.062 | 0.039 | NaN | 0.778 | 0.144 |  |
|  | P2 | 0.00 | 275.17 | 27.42 | 0.00 | 1011.63 | NaN | 0.071 | 0.778 | NaN | 0.126 |  |
| 0.2 | ABa | 0.00 | 323.52 | 277.43 | 0.00 | 1505.75 | NaN | 0.017 | 0.053 | NaN | 0.022 | 0.0629 |
|  | ABp | 323.52 | 0.00 | 237.88 | 275.73 | 1571.27 | 0.017 | NaN | 0.070 | 0.073 | 0.009 |  |
|  | EMS | 277.43 | 237.88 | 0.00 | 130.23 | 1326.73 | 0.053 | 0.070 | NaN | 0.056 | 0.140 |  |
|  | P2 | 0.00 | 275.73 | 130.23 | 0.00 | 1013.71 | NaN | 0.073 | 0.056 | NaN | 0.125 |  |
| 0.4 | ABa | 0.00 | 320.63 | 268.38 | 0.00 | 1501.60 | NaN | 0.026 | 0.019 | NaN | 0.025 | 0.0991 |
|  | ABp | 320.63 | 0.00 | 233.67 | 273.78 | 1564.36 | 0.026 | NaN | 0.086 | 0.066 | 0.013 |  |
|  | EMS | 268.38 | 233.67 | 0.00 | 171.04 | 1323.77 | 0.019 | 0.086 | NaN | 0.387 | 0.142 |  |
|  | P2 | 0.00 | 273.78 | 171.04 | 0.00 | 1010.88 | NaN | 0.066 | 0.387 | NaN | 0.127 |  |
| 0.6 | ABa | 0.00 | 320.25 | 263.22 | 0.00 | 1500.53 | NaN | 0.027 | 0.001 | NaN | 0.026 | 0.1286 |
|  | ABp | 320.25 | 0.00 | 232.16 | 273.47 | 1559.89 | 0.027 | NaN | 0.092 | 0.064 | 0.016 |  |
|  | EMS | 263.22 | 232.16 | 0.00 | 204.87 | 1322.64 | 0.001 | 0.092 | NaN | 0.662 | 0.143 |  |
|  | P2 | 0.00 | 273.47 | 204.87 | 0.00 | 1011.00 | NaN | 0.064 | 0.662 | NaN | 0.127 |  |
| 0.8 | ABa | 0.00 | 321.45 | 258.69 | 0.00 | 1500.85 | NaN | 0.024 | 0.018 | NaN | 0.026 | 0.1506 |
|  | ABp | 321.45 | 0.00 | 231.91 | 272.78 | 1557.32 | 0.024 | NaN | 0.093 | 0.062 | 0.018 |  |
|  | EMS | 258.69 | 231.91 | 0.00 | 228.01 | 1326.79 | 0.018 | 0.093 | NaN | 0.849 | 0.140 |  |
|  | P2 | 0.00 | 272.78 | 228.01 | 0.00 | 1011.38 | NaN | 0.062 | 0.849 | NaN | 0.127 |  |
|  |  |  |  |  |  |  |  |  |  |  |  |  |
|  |  | Area in Experiment $s$  (Pixel Number*Spatial Resolution^2^, μm^2^) | | | | |  |  |  |  |  |  |
|  |  | Contact | | | | Surface |  |  |  |  |  |  |
|  |  | ABa | ABp | EMS | P2 |  |  |  |  |  |  |  |
|  | ABa | 0.00 | 329.25 | 263.36 | 0.00 | 1540.24 |  |  |  |  |  |  |
|  | ABp | 329.25 | 0.00 | 255.71 | 256.91 | 1585.63 |  |  |  |  |  |  |
|  | EMS | 263.36 | 255.71 | 0.00 | 123.30 | 1542.56 |  |  |  |  |  |  |
|  | P2 | 0.00 | 256.91 | 123.30 | 0.00 | 1157.87 |  |  |  |  |  |  |

Note: $\bar{\delta}$ is the average of $\delta$ of all the surfaces and interfaces.
