# Supplementary material for: Computable early Caenorhabditis elegans embryo with a phase field model
Source: PLoS Comput Biol. 2022 Jan 14;18(1):e1009755. doi: 10.1371/journal.pcbi.1009755 (PMC8794267; doi:10.1371/journal.pcbi.1009755)
Supplement: S9 Table — (DOCX) [file pcbi.1009755.s029.docx]

**S9 Table. Preservation time of cell-cell contact map in simulation**

**with a single attraction motif added at 8-cell stage.**

| Attraction Motif at 8-Cell Stage | | Preservation Time of  Cell-Cell Contact Map (Time Step) | Corresponding Time  in Experiment (min) |
| --- | --- | --- | --- |
| Default | / | 38900 | 2.32 |
| ABal-ABar | (*σ*_ABal, ABar_ = 0.9) | 45300 | 2.71 |
| ABal-ABpl | (*σ*_ABal, ABpl_ = 0.2) | 40600 | 2.43 |
| ABal-MS | (*σ*_ABal, MS_ = 0.2) | 98900 | 5.91 |
| ABar-ABpl | (*σ*_ABar, ABpl_ = 0.2) | 53100 | 3.17 |
| ABar-ABpr | (*σ*_ABar, ABpr_ = 0.2) | 30800 | 1.84 |
| ABar-MS | (*σ*_ABar, MS_ = 0.2) | 11200 | 0.67 |
| ABpl-ABpr | (*σ*_ABpl, ABpr_ = 0.9) | 38200 | 2.28 |
| ABpl-MS | (*σ*_ABpl, MS_ = 0.2) | 20800 | 1.24 |
| ABpl-E | (*σ*_ABpl, E_ = 0.2) | 150500 | 8.99 |
| ABpl-C | (*σ*_ABpl, C_ = 0.2) | 3500 | 0.21 |
| ABpr-MS | (*σ*_ABpr, MS_ = 0.2) | 6900 | 0.41 |
| ABpr-E | (*σ*_ABpr, E_ = 0.2) | 24700 | 1.48 |
| ABpr-C | (*σ*_ABpr, C_ = 0.2) | 73200 | 4.37 |
| MS-E | (*σ*_MS, E_ = 0.9) | 46300 | 2.77 |
| E-P3 | (*σ*_E, P3_ = 0.2) | 35700 | 2.13 |
| C-E | (*σ*_C, E_ = 0.2) | 9200 | 0.55 |
| C-P3 | (*σ*_C, P3_ = 0.9) | 31800 | 1.90 |
