# Supplementary material for: Computable early Caenorhabditis elegans embryo with a phase field model
Source: PLoS Comput Biol. 2022 Jan 14;18(1):e1009755. doi: 10.1371/journal.pcbi.1009755 (PMC8794267; doi:10.1371/journal.pcbi.1009755)
Supplement: S10 Table — (DOCX) [file pcbi.1009755.s030.docx]

**S10 Table. Corresponding timing between simulations with**

***σ*_ABpl, E_ = 0.2, *σ*_ABpl, MS_ = 0.9 and *σ*_ABpl, E_ = 0.2, *σ*_ABpl, MS_ = 1.6 at 8-cell stage.**

| Attraction Motifs at 8-Cell Stage | *σ*_ABpl, E_ = 0.2, *σ*_ABpl, MS_ = 0.9 | *σ*_ABpl, E_ = 0.2, *σ*_ABpl, MS_ = 1.6 |
| --- | --- | --- |
| Corresponding Timing  between Two Simulations  (Time Step) | 192900 | 190500 |
|  | 209900 | 199800 |
|  | 226400 | 215000 |
|  | 233800 | 222300 |
|  | 243200 | 230200 |
|  | 248200 | 235400 |
|  | 271100 | 257700 |
|  | 284100 | 267300 |
|  | 294500 | 276000 |
|  | 310200 | 285000 |
|  | 326700 | 289300 |
|  | 335400 | 294700 |
|  | 336600 | 296000 |
|  | 338200 | 297700 |
|  | 345100 | 306500 |
|  | 348000 | 311300 |
